# Efficacy and safety of agents for IgA nephropathy: a network meta-analysis of randomized controlled trials

**DOI:** 10.3389/fmed.2025.1515723

**Published:** 2025-06-18

**Authors:** Bo Chen, Yan Zhu, Yang Yang, Gaosi Xu

**Affiliations:** ^1^Department of Nephrology, The Second Affiliated Hospital, Jiangxi Medical College, Nanchang University, Nanchang, China; ^2^Jiangxi Key Laboratory of Molecular Medicine, The Second Affiliated Hospital of Nanchang University, Nanchang, China

**Keywords:** IgA nephropathy, sodium-glucose cotransporter 2 inhibitors, telitacicept, nefecon, sparsentan

## Abstract

**Objective:**

IgA nephropathy (IgAN) is the leading cause of end-stage renal disease (ESRD) globally, with its pathological mechanisms closely related to mucosal immune abnormalities and complement activation. Currently, there is no curative treatment. This study aims to systematically evaluate the efficacy differences of existing treatment regimens on clinical remission (CR), 24-h urinary protein excretion (24-h UPE), ESRD or kidney damage (KD) and adverse events (AEs) in IgAN, providing evidence-based support for optimizing stratified treatment strategies.

**Methods:**

A systematic search was conducted in the PubMed, Web of Science, Embase, and Cochrane Library databases up to February 20, 2025, including 57 randomized controlled trials (RCTs) covering 19 interventions. Pairwise and network meta-analyses were employed to assess binary variable (CR, ESRD or KD, AEs) using risk ratios (RR) and continuous variable (24-h UPE) using standardized mean differences (SMD), with interventions ranked based on the area under the cumulative ranking curve.

**Results:**

Clinical remission (26 RCTs included in the analysis): The CR for tonsillectomy combined with steroids pulse therapy (TSP) (RR = 8.23, 95% CI 4.11–16.45), anti-APRIL monoclonal antibody sibeprenlimab (RR = 10.00, 1.34–74.48), and steroids combined with renin-angiotensin system inhibitors (STE + RASI) (RR = 5.03, 2.61–9.68) were significantly superior to placebo. Proteinuria control (36 studies assessing 24-h UPE): The BLyS/APRIL dual-target inhibitor telitacicept (SMD = −5.21, −7.55 to −2.87) and STE + RASI (SMD = −1.98, −3.15 to −0.82) significantly reduced 24-h UPE, outperforming the mycophenolate mofetil combined with steroids regimen (SMD = −0.97, −2.74 to 0.80). Renal endpoint events (26 studies analyzing ESKD or KD): STE + RASI reduced the risk of ESKD or KD by 98.1% (optimal SUCRA ranking), followed by the dual endothelin/angiotensin receptor antagonist sparsentan (82.6%). Safety (36 studies reporting adverse events): The complement inhibitor iptacopan (88.4%) and sodium-glucose co-transporter 2 inhibitors (SGLT2i) (85.4%) had the lowest incidence of adverse events, significantly better than immunosuppressive regimens.

**Conclusion:**

STE + RASI serves as a core therapeutic strategy for IgAN, significantly improving clinical remission rates, reducing the risk of ESRD or KD, and addressing proteinuria. Telitacicept, sparsentan, and TSP can be considered as enhanced options for specific phenotypic patients, while targeted ileal budesonide (Nefecon) has not demonstrated a significant renal protective advantage.

**Systematic review registration:**

CRD42023494801.

## 1 Introduction

IgA nephropathy (IgAN), the most prevalent primary glomerulonephritis worldwide, exhibits marked geographic heterogeneity in incidence. Asian populations, particularly in China and Japan, demonstrate a disproportionately high prevalence of IgAN, which ranks among the leading etiologies of glomerular diseases in these regions. The incidence in Asian cohorts significantly exceeds that observed in North America and Africa, a disparity strongly linked to interactions between genetic susceptibility (e.g., HLA-DQB1 polymorphisms) and environmental triggers (e.g., mucosal pathogen exposure) (1, 2). Notably, IgAN predominantly affects individuals under 40 years of age, emerging as a leading cause of kidney failure in young adults. Approximately 40% of patients progress to end-stage renal disease (ESRD) within 20–30 years, imposing substantial healthcare costs and socioeconomic burdens (3–5). Consequently, developing safe and effective disease-modifying therapies represents an urgent global priority.

Recent investigations have elucidated the autoimmune pathogenesis of IgAN. The central mechanism involves defective galactosylation of IgA1 as a key autoantigen (6, 7). Deficiencies in the expression of core 1β1,3-galactosyltransferase (C1GalT1) and its molecular chaperone, Cosmc, within patients’ B cells result in aberrant O-glycosylation of the IgA1 hinge region. The absence of galactose leads to the formation of pathogenic galactose-deficient IgA1 (Gd-IgA1) (8, 9). These abnormally glycosylated IgA1 molecules bind to anti-glycan autoantibodies (IgG/IgA), forming high-molecular-weight immune complexes. These immune complexes deposit in the glomeruli, activating the complement cascade and releasing inflammatory mediators. This process subsequently promotes mesangial cell proliferation and glomerular injury (10–12).

Given the aforementioned mechanisms, immunomodulatory therapies are considered potential intervention strategies, yet their clinical efficacy presents significant discrepancies. Large-scale paired meta-analyses indicate that conventional immunosuppressants, such as cyclophosphamide, although effective in reducing proteinuria, have not demonstrated the ability to decelerate the progression of renal dysfunction (13). Conversely, the TESTING study confirmed that glucocorticoids can reduce the risk of ESRD by 33%, but their long-term adverse effects limit their widespread application (14). The 2024 KDIGO guidelines propose a stratified management strategy, emphasizing the need for comprehensive interventions targeting multiple pathogenic pathways, including the reduction of circulating immune complex levels, inhibition of complement activation, and optimization of supportive care (e.g., the combined use of RASI and SGLT2i) (15). Novel therapies, such as dual endothelin-angiotensin receptor antagonists (sparsentan), have exhibited superior proteinuria-reducing effects compared to traditional RASIs, potentially offering new options for delaying the decline in renal function (16).

However, the current landscape of IgAN treatment still faces three major challenges. First, head-to-head comparisons are lacking for various novel drugs targeting Gd-IgA1 production, immune complex clearance, and complement regulation (e.g., APRIL/BLyS inhibitors, complement factor B antagonists). Secondly, the lack of standardized efficacy assessment criteria across studies, with current research often relying on proteinuria remission as a surrogate endpoint, necessitates further validation of its correlation with long-term renal outcomes. Finally, the optimal approach to precision medicine, based on individual differences such as biomarkers (Gd-IgA1 levels, genetic risk scores), pathological features, and lifestyle factors, remains undefined, leading to challenges in individualized treatment decision-making. Therefore, this study employs network meta-analysis (NMA) to systematically evaluate the efficacy and safety differences of various therapies, integrating both direct and indirect comparative evidence, with the aim of providing dynamically updated decision-making guidance for clinical practice.

## 2 Materials and methods

Our study followed PRISMA guidelines (17), detailed in the Supplementary materials S1, and is registered with PROSPERO (CRD42023494801).

### 2.1 Data sources and searches

We systematically searched databases such as PubMed, Web of Science, Embase, and Cochrane Library from inception to February 20, 2025, for randomized controlled trials (RCTs) evaluating the clinical efficacy of various agents in IgAN patients. There were no restrictions on language, publication years, or blinding methods. Our search strategy integrated MeSH terms and free text, including: [(Glomerulonephritis, IgA) OR (Berger’s Disease) OR (IgA Glomerulonephritis) OR (IgA Nephropathy)] AND [(Renin-Angiotensin System Inhibitors) OR (Steroids) OR (Telitacicept) OR (Sparsentan) OR (Mycophenolate Mofetil) OR (Budesonide/Nefecon) OR (Leflunomide) OR (Tacrolimus) OR (Hydroxychloroquine) OR (Tonsillectomy) OR (Rituximab) OR (Mizoribine) OR (Cyclosporin A) OR (Azathioprine) OR (Cyclophosphamide) OR (Atacicept) OR (Iptacopan) OR (Sodium-glucose cotransporter 2 inhibitors) OR (Tonsillectomy with steroid pulse therapy) OR (Sibeprenlimab)] AND (RCTs). We also manually reviewed the literature to ensure comprehensive coverage.

### 2.2 Selection criteria

The inclusion criteria (Supplementary materials S3) are as follows: (1) study type was RCTs; (2) the study participants were no less than 9 years old, no gender limit, renal biopsy confirmed IgAN; (3) the subjects with proteinuria or 24-h urinary protein excretion (24-h UPE) more than 0.5 g/d and eGFR ≥ 30 mL/min per 1.73 m^2^ or serum creatinine less than 3.5 mg/dL; (4) interventions for studies should include RASI (renin-angiotensin system inhibitors), STE (steroids), MMF (mycophenolate mofetil), AZA (azathioprine), CsA (Cyclosporin A), Tonsillectomy, RIT (rituximab), TAC (tacrolimus), HCQ (hydroxychloroquine), LEF (leflunomide), MZR (mizoribine), CTX (cyclophosphamide), TSP (tonsillectomy with steroid pulse therapy), nefecon, telitacicept, sparsentan, atacicept, iptacopan and sibeprenlimab; (5) each study reported at least one of the following four indicators: (1) clinical remission (CR; defined as achieving proteinuria <0.3 g/d or a ≥50% reduction in proteinuria), (2) 24-h UPE, (3) ESRD (defined by serum creatinine >707 μmol/L or requirement for maintenance dialysis or kidney transplantation) or kidney damage (KD; defined by either a ≥30% decrease in eGFR from baseline or a doubling of serum creatinine), (4) adverse events (AEs).

Exclusion criteria: (1) clinically confirmed IgAN secondary to systemic diseases such as systemic lupus erythematosus, and allergic purpura; (2) drugs included in the publication were not involved in our study, or publications compared to the same drug in terms of administration route or dosage; (3) articles that had no definitions on clinical remission or 24-h UPE or renal function.

### 2.3 Data extraction and quality evaluation

We used EndNote software to manage the retrieved literature. After screening the title and abstract, the articles meeting the inclusion criteria were obtained for evaluation and data extraction. In addition, three reviewers (BC, YZ, and YY) independently extracted data through Microsoft Excel. Any disagreements during data extraction were resolved by the fourth reviewer (GX). The data extraction contents included: basic characteristics of the included literature (country, publication year, and first author), study subject information (mean age, sample size, renal function, and baseline of UPE), interventions (different drugs and period of follow-up), and reported outcomes (CR, 24-h UPE, ESRD or KD, and AEs). For information that cannot be directly obtained, we made great efforts to contact the authors via email. The three reviewers (BC, YZ, and YY) independently assessed the risk of bias for all studies according to the Cochrane Risk of Bias tool [Cochrane Handbook for Systematic Reviews of Interventions, version 5.4.0] (18). Each domain can be evaluated as high, low, or unclear risk for the included studies. Any disagreements were resolved by the fourth reviewer (GX).

### 2.4 Statistical analysis

The data from studies reporting outcomes like clinical remission, ESRD or KD, 24-h UPE, and AEs were extracted. Using a frequentist framework, a random-effects model analyzed the data (19). We calculated the 95% confidence intervals (CI) and relative risk (RR) for dichotomous variables, along with 95% CI and standardized mean difference (SMD) for continuous variables to quantify effect sizes. Statistical analysis was conducted using STATA 17.0, with “mvmeta” and “network” packages for network plots, league tables, publication bias assessment, and treatment ranking probabilities. The Surface Under the Cumulative Ranking curve (SUCRA), which ranges from 0 (worst) to 1 (best) (20), evaluated treatment efficacy, with higher values indicating better strategies. R version 4.2.3, employing “ggplot2” and “gemtc” packages, generated forest plots and regression analyses. For initial values, we executed 50,000 simulations, discarding the first 20,000 as burn-in. Convergence was assessed with Brooks-Gelman-Rubin diagnostic plots. Statistical significance was noted when zero was excluded in the 95% CI for SMD or one for RR. Some heterogeneity was expected; thus, we employed a “design-by-treatment” model (21) for global assessment and node-splitting methods (22) for local analysis, separating direct and indirect comparisons. A *p*-value above 0.05 indicated no significant inconsistency (23). Following this, a consistency model was applied to analyze remaining statistical data. Heterogeneity was measured using *I*^2^, with *I*^2^ > 50% indicating significant diversity among RCTs. In our findings, *I*^2^ values for AEs, clinical remission, ESRD or KD, and 24-h UPE were all below 12%, reflecting low heterogeneity (Supplementary materials S5). Regression and sensitivity analyses were conducted with R and STATA to investigate potential heterogeneity sources.

### 2.5 General classification of drugs

We categorized interventions into four efficacy groups based on SUCRA rankings for AEs, clinical remission, ESRD or KD, and 24-h UPE: significant efficacy (SUCRA > 60% for two or more indicators), moderate efficacy (SUCRA 40–60% for two or more indicators), low efficacy (SUCRA < 40% for two or more indicators), and very low efficacy (lowest SUCRA rankings for two indicators), as shown in Supplementary materials S8 and Supplementary Figure 8.

## 3 Results

### 3.1 Literature screening process

**Figure 1:**
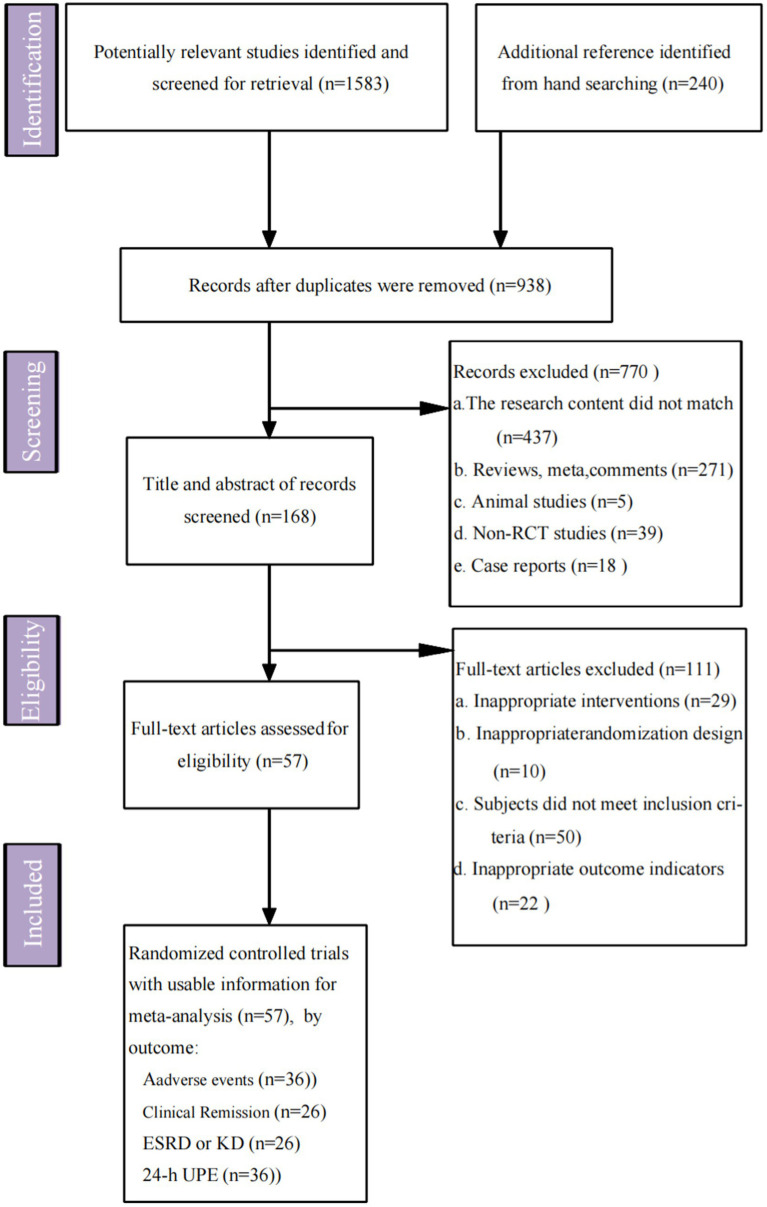
Flow chart of literature screening process and results. ESRD, end-stage renal disease; KD, kidney damage; 24-h UPE, 24-h urinary protein excretion; randomized controlled trials (RCTs).

A total of 1,823 articles were identified. After deduplication using EndNote, 938 articles remained. Excluding animal studies, reviews, case reports, and non-RCTs through title, keyword, and abstract screening, 168 articles were selected. Upon reviewing these 168 full texts, 111 were excluded for reasons such as study design, treatment protocols, subjects, and outcome measures not aligning with our criteria. Ultimately, 57 RCTs (24–80) (including one three-arm RCT and 56 two-arm RCTs) involving 5,123 patients were included. These trials investigated 19 different interventions (excluding combination therapies), such as TSP, MMF, STE, RASI, LEF, CsA, MZR, RIT, HCQ, AZA, TAC, SGLT2i, iptacopan, atacicept, telitacicept, sparsentan, sibeprenlimab, nefecon, and placebo. The literature screening process and results are illustrated in Figure 1.

### 3.2 Patient and baseline characteristics

In these 57 RCTs, TSP was used in 4 RCTs involving 221 patients (4 RCTs, 221 patients), MMF (10 RCTs, 2,331 patients), STE (28 RCTs, 1,753 patients), RASI (20 RCTs, 1,464 patients), LEF (4 RCTs, 143 patients), CsA (2 RCTs, 32 patients), MZR (4 RCTs, 141 patients), RIT (1 RCT, 17 patients), HCQ (2 RCTs, 120 patients), AZA (5 RCTs, 202 patients), TAC (1 RCT, 20 patients), SGLT2I (1 RCT, 137 patients), iptacopan (1 RCT, 26 patients), atacicept (1 RCT, 5 patients), telitacicept (1 RCT, 14 patients), sparsentan (1 RCT, 202 patients), sibeprenlimab (1 RCT, 38 patients), nefecon (1 RCT, 182 patients), and STE combined with RASI (3 RCTs, 101 patients). Among these, 26 RCTs reported detailed information regarding clinical remission and ESRD or KD, and 36 RCTs reported baseline and post-treatment 24-h UPE. Additionally, 36 RCTs mentioned different adverse reactions in both the treatment and control groups. The relevant baseline characteristics of the included 57 RCTs can be found in Supplementary materials S2. In this study, all but two RCTs included mentioned randomization. In addition, 18 RCTs (32% of all RCTS) described specific randomization methods, of which 5 RCTs used random number tables, 1 RCT used stratified random sampling, and 12 RCTs used computer-generated randomization, all of which were rated as “low risk” bias in the random sequence generation section. All RCTs that provided complete data and did not selectively report results were rated as “low risk” of bias in the areas of full outcome assessment and selective reporting. However, due to a lack of sufficient information, most RCTs were rated as “uncertain risk” in terms of implementation bias, measurement bias, and other biases. The risk of bias for the eligible studies is presented in Supplementary Figure 1.

### 3.3 Network structure, consistency, and heterogeneity

**Figure 2:**
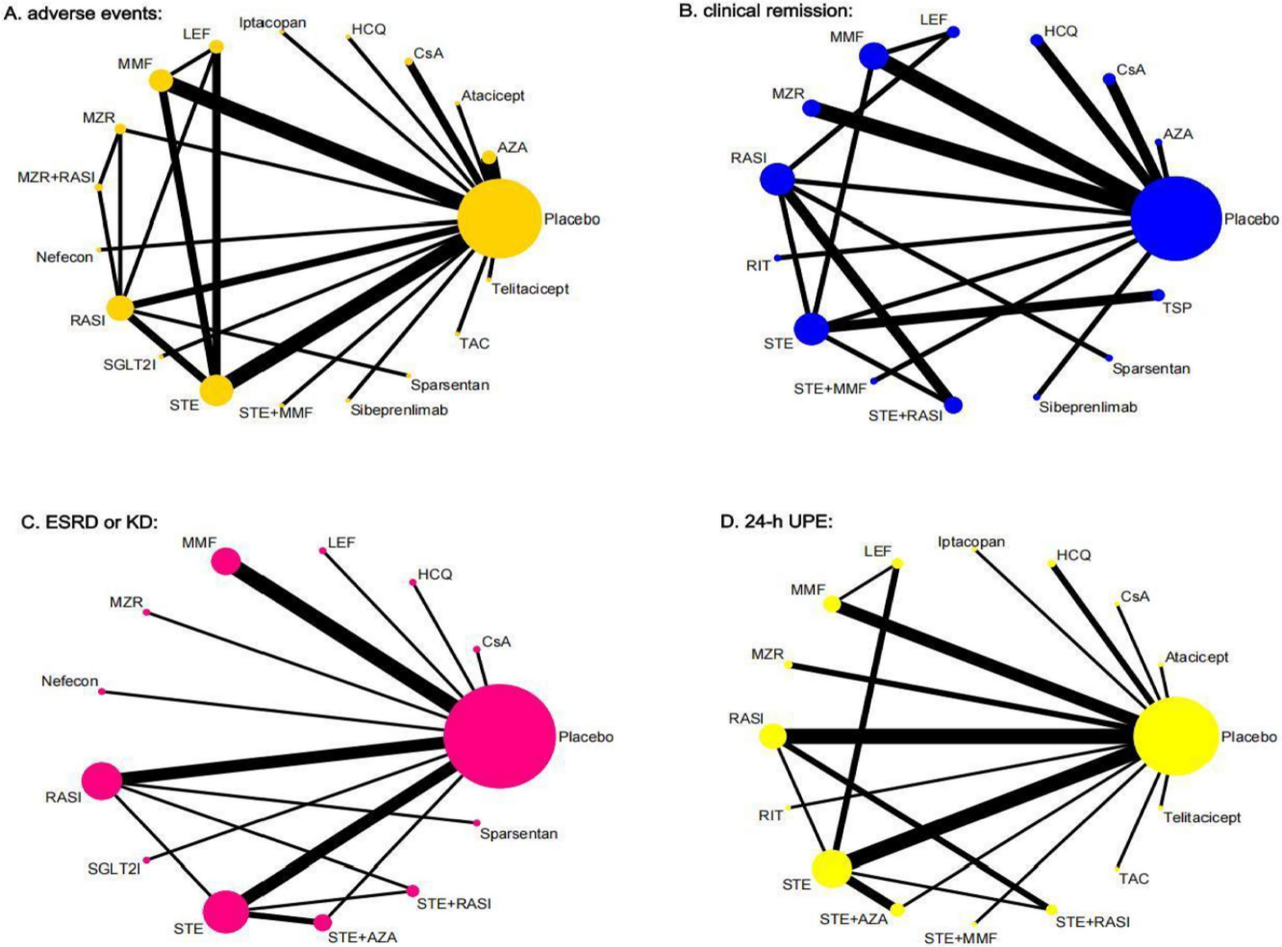
Network meta-analysis of eligible comparisons for **(A)** adverse events, **(B)** clinical remission, **(C)** ESRD or KD, **(D)** 24-h UPE. The width of the lines represents the number of each pairwise comparison. The size of each node is proportional to the number of randomly assigned participants (i.e., sample size). TSP, tonsillectomy with steroid pulse therapy; MMF, mycophenolate mofetil; STE, steroids; RASI, renin-angiotensin system inhibitors; LEF, leflunomide; CsA, Cyclosporin A; MZR, mizoribine; RIT, rituximab; HCQ, hydroxychloroquine; AZA, azathioprine; TAC, Tacrolimus; SGLT2i, sodium glucose cotransporter-2 inhibitor.

The network diagrams for various interventions are presented in Figure 2, illustrating 19 interventions for adverse events, 15 for clinical remission, 13 for ESRD or KD, and 16 for 24-h UPE. Node sizes reflect the sample sizes of the interventions, while line thickness indicates the number of direct comparisons between interventions. The sample sizes and number of RCTs differ across interventions. Diagnostic plots and trace plots confirm satisfactory convergence of this NMA, as detailed in Supplementary Figure 3. Consistency analysis via node-splitting methods (Supplementary Figure 5) shows all *p*-values exceeding 0.05, indicating strong consistency, except for comparisons involving STE and placebo, STE with RASI, and RASI, STE in ESRD or KD. Heterogeneity analysis (Supplementary Figure 4) reveals significant heterogeneity in comparisons: CsA vs. placebo; STE vs. placebo; RASI vs. placebo; RASI vs. STE; MMF vs. STE in AEs; RASI vs. placebo; STE vs. MMF, RASI, TSP in CR; MMF, STE, STE with AZA vs. placebo; STE vs. STE with AZA, RASI in ESRD or KD; HCQ, MMF, MZR, STE, AZA with AZA vs. placebo; STE vs. LEF, RASI, STE with AZA, STE with RASI; STE with RASI vs. RASI in 24-h UPE. Consequently, a Random-Effects model was chosen for the NMA, with potential heterogeneity sources investigated through regression and sensitivity analyses.

### 3.4 Pairwise meta-analysis

The pairwise meta-analysis results for various agents are in Supplementary materials S6.

### 3.5 Network meta-analysis results

#### 3.5.1 Adverse events

A total of 36 studies on adverse events involved 3,891 patients across 19 interventions: Placebo (26 RCTs, 1,294 patients), AZA (4 RCTs, 173 patients), atacicept (1 RCT, 5 patients), CsA (2 RCTs, 32 patients), HCQ (1 RCT, 30 patients), iptacopan (1 RCT, 26 patients), LEF (4 RCTs, 143 patients), MMF (7 RCTs, 209 patients), MZR (2 RCTs, 54 patients), MZR + RASI (1 RCT, 30 patients), nefecon (1 RCT, 182 patients), RASI (7 RCTs, 479 patients), SGLT2i (1 RCT, 137 patients), STE (10 RCTs, 425 patients), STE + MMF (1 RCT, 26 patients), sibeprenlimab (1 RCT, 38 patients), sparsentan (1 RCT, 202 patients), TAC (1 RCT, 20 patients), and telitacicept (1 RCT, 14 patients). The network diagram is shown in Figure 2A.

**Figure 3:**
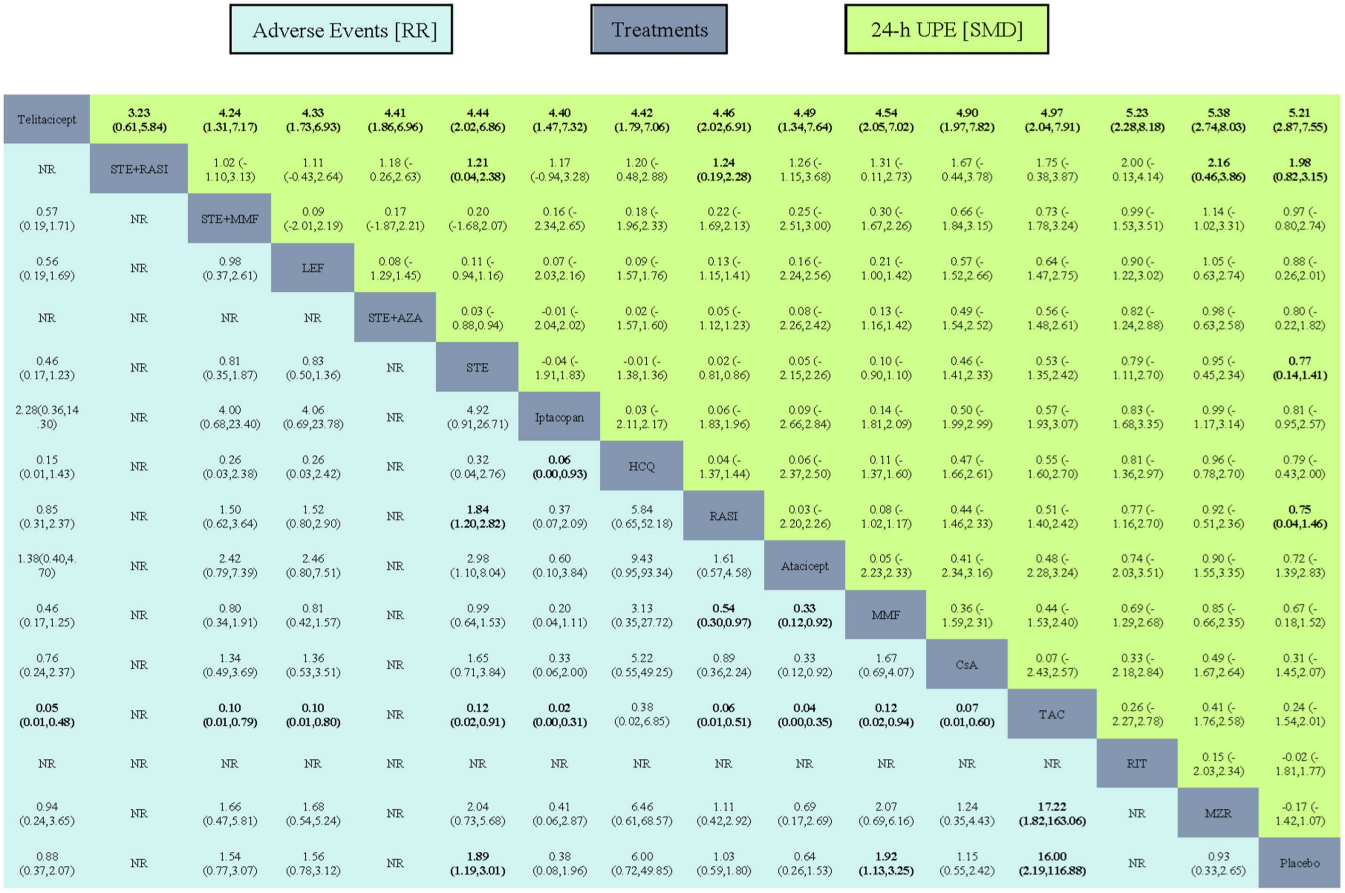
League table of all comparisons for adverse events and 24-h UPE. Data are RR (95% CI) for adverse events (lower-left quadrant) and SMD (95% CI) for 24-h UPE (upper-right quadrant) in the column-defining treatment compared with the row-defining treatment. RR lower than one favor the column-defining treatment and SMD higher than zero favor the row-defining treatment. Significant results are indicated in bold. SMD, standardized mean difference.

TAC had a higher incidence of adverse reactions compared with all other interventions. The RR of iptacopan, SGLT2i, atacicept, telitacicept, Placebo, MZR, RASI, sparsentan, CsA, sibeprenlimab, MZR + RASI, AZA, STE + MMF, LEF, nefecon, MMF, STE, and HCQ were 41.60 (95%CI: 3.19, 542.94), 25.47 (3.10, 209.60), 25.14 (2.86, 221.14), 18.29 (2.10, 159.52), 16.00 (2.19, 116.88), 17.22 (1.82, 163.06), 15.57 (1.97, 122.90), 14.85 (1.71, 128.87), 13.93 (1.67, 116.38), 13.94 (1.76, 110.19), 11.55 (1.19, 112.53), 10.96 (1.42, 84.44), 10.40 (1.27, 85.35), 10.24 (1.25, 84.06), 8.00 (0.89, 71.70), 8.34 (1.07, 65.25), 8.45 (1.10, 65.12), and 2.67 (0.15, 48.69), respectively (Figure 3).

**Figure 4:**
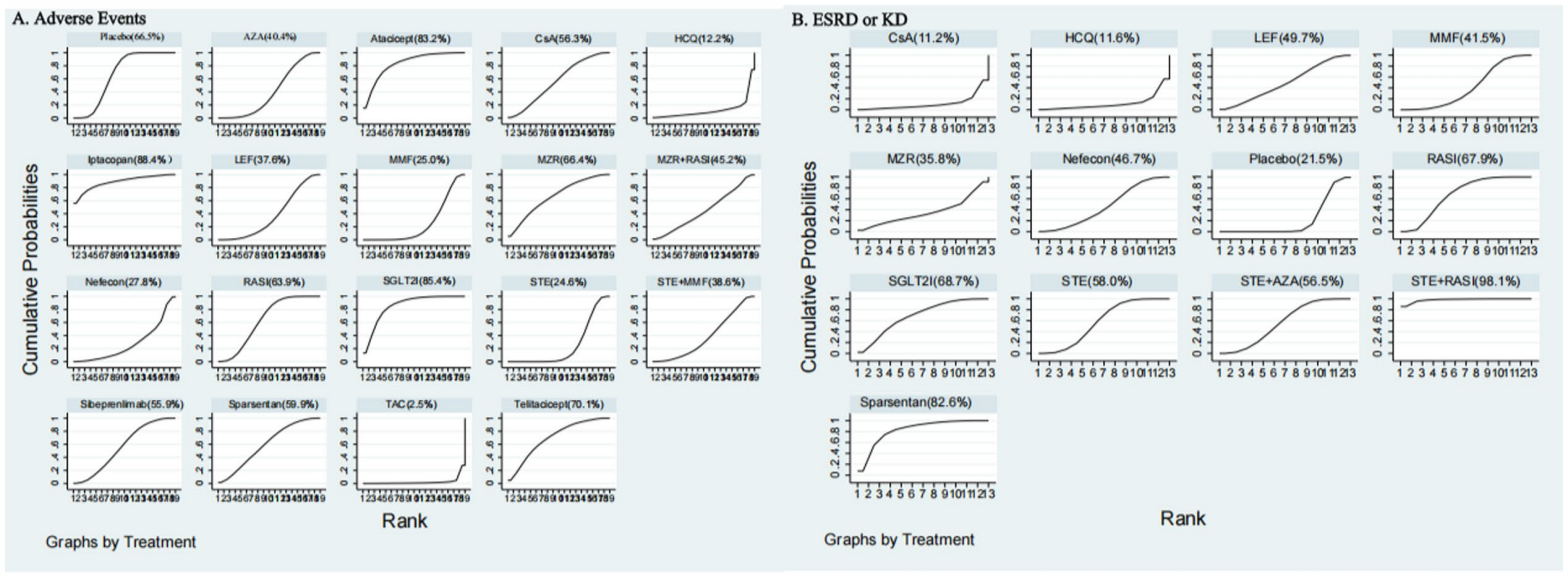
Rankings of SUCRA for **(A)** adverse events and **(B)** ESRD or KD. SUCRA, surface under the cumulative ranking curve.

Supplementary materials S4A displays SUCRA values for the interventions, where iptacopan scored 88.4%, SGLT2I 85.4%, Atacicept 83.2%, telitacicept 70.1%, Placebo 66.5%, MZR 66.4%, RASI 63.9%, sparsentan 59.9%, CsA 56.3%, sibeprenlimab 55.9%, MZR + RASI 45.2%, AZA 40.4%, STE + MMF 38.6%, LEF 37.6%, nefecon 27.8%, MMF 25.0%, STE 24.6%, HCQ 12.2%, and TAC 2.5%. Detailed statistical analysis results are illustrated in Figure 4A and Supplementary Figure 2A.

#### 3.5.2 Clinical remission

A total of 26 studies reported the clinical remission outcome, with 1,996 patients involved. The analysis included 15 interventions: placebo (16 RCTs, 466 patients), AZA (1 RCT, 40 patients), CsA (2 RCTs, 32 patients), HCQ (2 RCTs, 120 patients), LEF (3 RCTs, 84 patients), MMF (5 RCTs, 113 patients), MZR (3 RCTs, 72 patients), RASI (6 RCTs, 409 patients), RIT (1 RCT, 17 patients), STE (9 RCTs, 289 patients), STE + MMF (1 RCT, 26 patients), STE + RASI (3 RCTs, 135 patients), sibeprenlimab (1 RCT, 38 patients), sparsentan (1 RCT, 202 patients), and TSP (1 RCT, 49 patients). The network diagram is detailed in Figure 2B.

**Figure 5:**
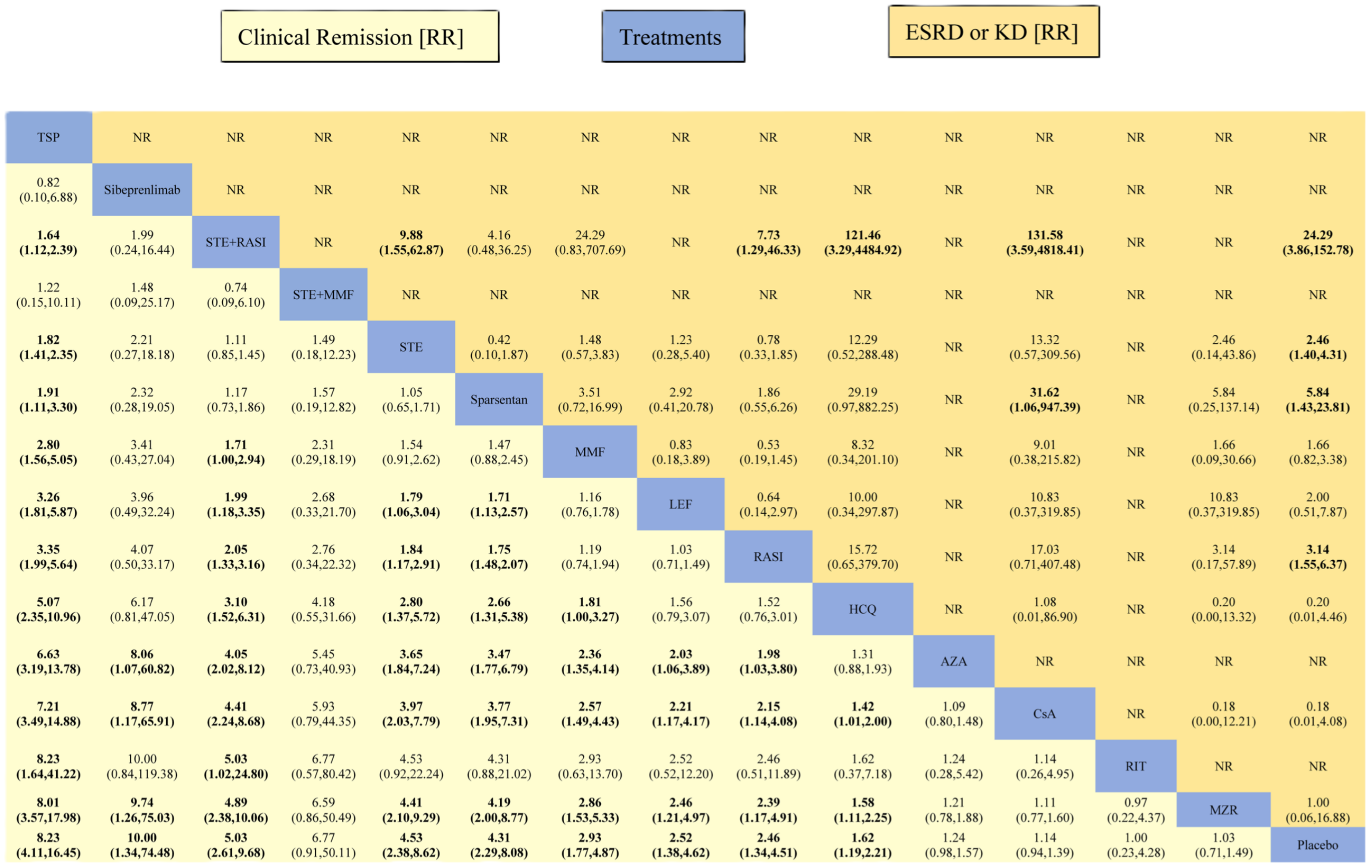
League table of all comparisons for clinical remission and ESRD or KD. Data are RR (95% CI) for clinical remission (lower-left quadrant) and ESRD or KD (upper-right quadrant) in the column-defining treatment compared with the row-defining treatment. RR higher than one favor the column-defining treatment (lower-left quadrant). RR higher than one favor the row-defining treatment (upper-right quadrant). Significant results are indicated in bold. RR, risk ratios; 95% CI, 95% confidence intervals.

Except for STE + MMF, AZA, CsA, RIT, and MZR, all other interventions demonstrated superior efficacy in achieving CR compared to placebo. The RR for TSP, sibeprenlimab, STE + RASI, STE, sparsentan, MMF, LEF, RASI, and HCQ are 8.23 (95% CI: 4.11, 16.45), 10.00 (1.34, 74.48), 5.03 (2.61, 9.68), 4.53 (2.38, 8.62), 4.31 (2.29, 8.08), 2.93 (1.77, 4.87), 2.52 (1.38, 4.62), 2.46 (1.34, 4.51), and 1.62 (1.19, 2.21), respectively (Figure 5).

**Figure 6:**
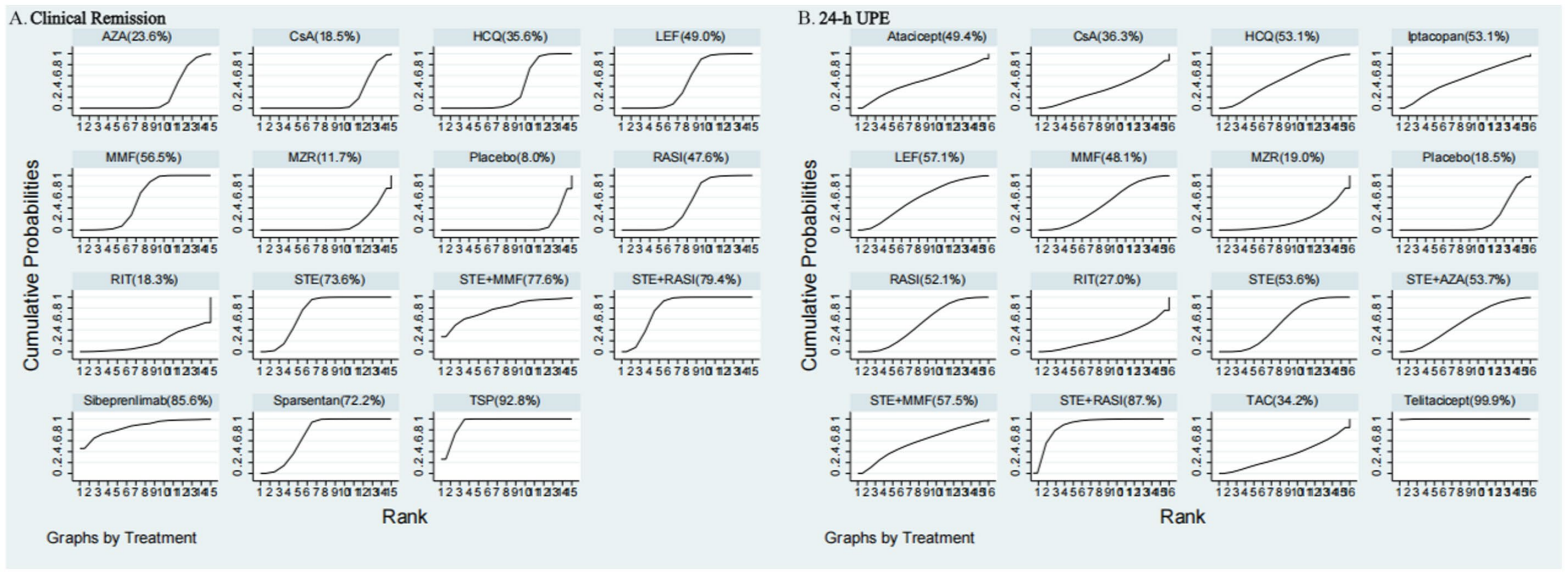
Rankings of SUCRA for **(A)** clinical remission and **(B)** 24-h UPE.

The Supplementary materials S4B presented SUCRA values for 15 interventions concerning CR: TSP 92.8%, sibeprenlimab 85.6%, STE + RASI 79.4%, STE + MMF 77.6%, STE 73.6%, sparsentan 72.2%, MMF 56.5%, LEF 49.0%, RASI 47.6%, HCQ 35.6%, AZA 23.6%, CsA 18.5%, RIT 18.3%, MZR 11.7%, and Placebo 8.0%. Detailed results are shown in Figure 6A and Supplementary Figure 2B.

#### 3.5.3 ESRD or KD

A total of 26 studies analyzed this indicator, involving 3,250 patients across 13 interventions: Placebo (20 RCTs, 1,188 patients), CsA (1 RCT, 23 patients), HCQ (1 RCT, 30 patients), LEF (2 RCTs, 80 patients), MMF (5 RCTs, 229 patients), MZR (1 RCT, 21 patients), nefecon (1 RCT, 182 patients), RASI (7 RCTs, 432 patients), SGLT2I (1 RCT, 137 patients), STE (11 RCTs, 662 patients), STE + AZA (3 RCTs, 140 patients), STE + RASI (2 RCTs, 53 patients), and sparsentan (1 RCT, 202 patients). The network visualization is shown in Figure 2C.

All interventions except LEF, nefecon, MMF, MZR, HCQ, and CsA had a lower incidence of ESRD or KD compared to Placebo. The RR for STE + RASI, sparsentan, SGLT2i, RASI, STE, and STE + AZA were recorded as 0.04 (0.01, 0.26), 0.17 (0.04, 0.70), 0.29 (0.09, 0.97), 0.32 (0.16, 0.64), 0.41 (0.23, 0.71), and 0.42 (0.20, 0.86), respectively (Figure 5).

Supplementary materials S4C provides the SUCRA values for 15 ESRD or KD interventions, showing percentages as follows: STE + RASI 98.1%, sparsentan 82.6%, SGLT2I 68.7%, RASI 67.9%, STE 58.0%, STE + AZA 56.5%, LEF 49.7%, nefecon 46.7%, MMF 41.5%, MZR 35.8%, Placebo 21.5%, HCQ 11.6%, CsA 11.2%. Detailed statistical results are in Figure 4B and Supplementary Figure 2C.

#### 3.5.4 24-h UPE

There are 36 studies involving 2,568 patients assessed 24-h UPE across 16 interventions: Placebo (26 RCTs, 911 patients), Atacicept (1 RCT, 5 patients), CsA (2 RCTs, 23 patients), HCQ (2 RCTs, 120 patients), iptacopan (1 RCT, 26 patients), LEF (3 RCTs, 119 patients), MMF (5 RCTs, 103 patients), MZR (2 RCTs, 51 patients), RASI (8 RCTs, 288 patients), RIT (1 RCT, 17 patients), STE (12 RCTs, 630 patients), STE + AZA (4 RCTs, 91 patients), STE + MMF (1 RCT, 26 patients), STE + RASI (3 RCTs, 124 patients), TAC (1 RCT, 20 patients), and telitacicept (1 RCT, 14 patients) (Figure 2D).

All interventions, except for telitacicept, exhibited lower effects on proteinuria reduction. The relative risk (Standardized Mean Difference, SMD) for STE + RASI, STE + MMF, LEF, STE + AZA, STE, iptacopan, HCQ, RASI, Atacicept, MMF, CsA, TAC, RIT, MZR, and Placebo were −3.23 (95% CI: −5.84, −0.61), −4.24 (−7.17, −1.31), −4.33 (−6.93, −1.73), −4.41 (−6.96, −1.86), −4.44 (−6.86, −2.02), −4.40 (−7.32, −1.47), −4.42 (−7.06, −1.79), −4.46 (−6.91, −2.02), −4.49 (−7.64, −1.34), −4.54 (−7.02, −2.05), −4.90 (−7.82, −1.97), −4.97 (−7.91, −2.04), −5.23 (−8.18, −2.28), −5.38 (−8.03, −2.74), and −5.21 (−7.55, −2.87), respectively (Figure 3).

Supplementary materials S4D presents the SUCRA values for the 16 interventions concerning the 24-h UPE, which were 99.9, 87.4, 57.5, 57.1, 53.7, 53.6, 53.1, 53.1, 52.1, 49.4, 48.1, 36.3, 34.2, 27.0, 19.0, and 18.5% for telitacicept, STE + RASI, STE + MMF, LEF, STE + AZA, STE, iptacopan, HCQ, RASI, Atacicept, MMF, CsA, TAC, RIT, MZR, and Placebo, respectively. Detailed statistical analysis results can be found in Figure 6B and Supplementary Figure 2D.

### 3.6 Meta-regression, publication bias and sensitivity analyses

We performed a subgroup analysis in IgA patients with proteinuria > 1 g/d, and there was a non-significant difference compared with the group with proteinuria > 0.5 g/d. Heterogeneity tests indicated significant differences between subgroups, necessitating further investigation into heterogeneity sources via regression analysis. We adjusted for publication year and sample size as univariate covariates regarding AEs, CR, ESRD or KD, and 24-h UPE. Results indicated that publication year and sample size correlated with heterogeneity in AEs, CR, and ESRD or KD but not in 24-h UPE (Supplementary materials S7). An adjusted funnel plot indicated no significant publication bias (Supplementary Figure 6). Sensitivity analyses showed excluding any single study did not significantly alter the overall effect size, confirming the robustness of our findings (Supplementary Figure 7).

## 4 Discussion

This conducted a stratified analysis of 19 interventions based on SUCRA, categorizing drug efficacy into four groups according to the comprehensive rankings of AEs, CR, ESRD or KD, and 24-h UPE. Firstly, the significant efficacy group (SUCRA >60%) includes SGLT2i, telitacicept, sparsentan, and STE + RASI. Their core mechanisms encompass metabolic regulation (SGLT2i), immune complex clearance (telitacicept) (81), and hemodynamic optimization (sparsentan), which significantly reduce the risk of ESRD and 24-h UPE. Secondly, the moderate efficacy group (SUCRA 40–60%) consists of the complement inhibitor iptacopan (C5a antagonist), the anti-APRIL monoclonal antibody sibeprenlimab, and traditional immunotherapy regimens (STE monotherapy/combined with mycophenolate mofetil/azathioprine), indicating that some targeted therapies require adjunctive support to enhance efficacy. Then, the low efficacy group includes nefecon and RIT, potentially related to heterogeneous responses in mucosal immune regulation. Finally, the very low efficacy group comprises HCQ and calcineurin inhibitors (TAC, CsA), reflecting the limitations of nonspecific immunosuppression in IgAN.

Based on KDIGO guidelines (15) and the latest study, treatment strategies for IgAN should prioritize a stratified selection that balances efficacy and safety. Our research demonstrates that STE + RASI, as a classic immunomodulatory regimen, significantly outperforms traditional immunosuppressive therapies (such as STE monotherapy, MMF, LEF, CsA, etc.) in terms of CR, ESRD or KD, and 24-h UPE, with mechanisms involving immune modulation (Th17/IL-23 pathway inhibition) and hemodynamic optimization (reduction of intraglomerular pressure), consistent with finding from Horita et al. (35). Furthermore, previous meta-analyses (82) indicate that immunosuppressive therapy can reduce the long-term risk of ESRD in IgAN patients, although it may increase the risk of long-term adverse events. However, strict monitoring of glucocorticoid-related adverse events is necessary, and it is recommended to use low-dose STE (0.4–0.6 mg/kg/d) for a limited duration (6–9 months), prioritizing high-risk patients with proteinuria ≥1 g/d and eGFR ≥60 mL/min/1.73 m^2^ (KDIGO 2024 2B recommendation) (15), aligning with the results of the low-dose group in the TESTING study (NEJM 2022) (59).

Although nefecon (targeting budesonide in the ileum) reduced Gd-IgA1 levels by 53% in phase III trials (NEFIGAN study) (62), this meta-analysis indicates its overall efficacy is relatively low (low efficacy group), possibly due to the inability to extract specific data regarding CR and 24-h UPE during the analysis. KDIGO 2024 recommends its use in subgroups with biopsy-confirmed active mesangial proliferation (M1) or C1/C2 (capillary wall lesions) and Gd-IgA1 ≥ 2.5 U/mL, rather than as a broad replacement for traditional regimens. Nevertheless, the unique advantage of nefecon in reducing Gd-IgA1 and IgA immune complex levels should not be overlooked, as it holds immense potential as a drug that can block the progression of IgAN at its source.

For high-risk patients, intensified treatment regimens should be considered, such as the combination of SGLT2i and RASI. In the KDIGO 2024 draft, this combination has been upgraded to first-line support therapy. This combination has been shown to reduce the risk of ESRD and cardiovascular events (83), independent of its hypoglycemic effects. This is particularly suitable for patients with progressive disease with eGFR ≥25 mL/min/1.73 m^2^. Although the safety of SGLT2 inhibitors in this study was good (SUCRA 85.4%), adverse reactions such as urinary tract infections, worsening renal injury, and blood volume reduction should be noted. On the one hand, our research results show that, compared to RASI, sparsentan, RASI + STE, iptacopan, telitacicept has advantages in reducing 24-h UPE, with fewer side effects and good safety. For patients with persistent nephropathy accompanied by significant proteinuria (UPCR ≥3.5 g/g), telitacicept (BLyS/APRIL inhibitor) (84) combined with RASI can additionally reduce proteinuria by 47% (SMD = −5.21%). On the other hand, this study shows that sparsentan (ETAR/AT1R antagonist) may be superior to traditional RASI in CR and the prevention of renal progression (16, 85). Therefore, sparsentan can be used in patients resistant to RASI, which can effectively slow the rate of eGFR decline, reaching 2.4 mL/min/year (PROTECT study).

Meanwhile, iptacopan (SUCRA 88.4%) performed best in terms of safety. For patients with high complement activation markers (elevated serum C3a/C5a), complement inhibitors (iptacopan) can be used as an alternative for those who are intolerant to hormones. This study also explored the role of tonsillectomy in the treatment of IgAN. The results show that, whether as an adjuvant therapy or as a standalone treatment, tonsillectomy can significantly improve the remission rate of proteinuria and hematuria. This finding is consistent with the results of the Japanese population (86), suggesting that the IgA1 secreted by the tonsil cells may be involved in the pathogenesis of IgAN (87, 88), which aligns with the conclusions drawn from the meta-analysis conducted by Wang (89), Liu (90), and others. Therefore, for patients with recurrent tonsil attacks, tonsillectomy may be an effective alternative or supplementary treatment.

Despite the inclusion of 57 RCTs and 5,123 participants in this study, certain limitations persist. Firstly, heterogeneity in patient baseline characteristics and treatment regimens across different studies may impact the generalizability of the findings. Secondly, long-term efficacy and safety data for some medications remain insufficient, necessitating further validation through larger, multi-center randomized controlled trials. Moreover, future research should strictly adhere to PRISMA guidelines, providing detailed baseline data to support more robust network meta-analyses.

In summary, while Yang (91) and Tan (92) have previously published network meta-analyses on various interventions for IgAN patients, this study provides crucial clinical evidence regarding the efficacy and safety of pharmacological treatments for IgAN. Emerging drugs such as nefecon, telitacicept, and sparsentan have demonstrated significant advantages in reducing proteinuria, preventing ESRD, and improving renal function recovery, while traditional medications like RASI and STE continue to hold an important position (82). However, clinical practice must integrate the Oxford classification (MEST-C), biomarkers (Gd-IgA1, complement activation products), patient comorbidities, cost, and accessibility to formulate optimal treatment strategies. Future research should continue to focus on the long-term efficacy and safety of these medications, providing a more solid evidence base for clinicians and patients.

## 5 Limitations

The limitations of this study include: (1) Among 57 studies, 39 (68.4%) lacked details on randomization, risking selection bias; (2) Heterogeneity among studies, despite regression and sensitivity analyses; (3) Missing baseline data like proteinuria and GFR changes; (4) Inadequate description of blinding and allocation concealment, risking information bias. Future high-quality, multicenter, large-sample RCTs are needed for more reliable clinical evidence.

## 6 Conclusion

STE + RASI, as a classic immunomodulatory regimen, demonstrates significant advantages in comprehensive clinical remission (79.4%), ESRD or KD (98.1%), and reduction of 24-h UPE (87.4%); however, its infection and metabolism-related adverse events require close monitoring. Compared to other treatment regimens, sparsentan (82.6%) shows potential superiority in preventing end-stage renal disease; Telitacicept (99.9%) excels in reducing 24-h UPE and may be suitable for patients with persistent proteinuria; iptacopan (88.4%) and SGLT2i (85.4%) provide additional advantages in terms of safety. Additionally, for IgAN patients with recurrent tonsillitis, TSP (92.8%) may be the best option for improving clinical remission rates. Nefecon, as a targeted therapy, has not yet been shown in our studies to be superior to traditional immunosuppressive regimens in delaying eGFR decline and overall safety.

## Data Availability

The original contributions presented in the study are included in the article/Supplementary material, further inquiries can be directed to the corresponding author.

## Glossary

AEs: Adverse events
APRIL: A proliferation inducing ligand
AZA: Azathioprine
BlyS: B lymphocyte stimulator
CI: Confidence intervals
CsA: Cyclosporin A
CR: Clinical remission
eGFR: Estimated glomerular filtration rate
ESRD: End-stage renal disease
Gd-IgA1: Galactose-deficient IgA1
HCQ: Hydroxychloroquine
IgAN: IgA nephropathy
KD: Kidney damage
KDIGO: Kidney Disease Improving Global Outcomes
LEF: Leflunomide
MMF: Mycophenolate mofetil
MZR: Mizoribine
NMA: Network meta-analysis
UPE: Urinary protein excretion
RASI: Renin-angiotensin system inhibitors
RCTs: Randomized controlled trials
RIT: Rituximab
RR: Risk ratios
SGLT2i: Sodium glucose cotransporter-2 inhibitor
SMD: Standardized mean difference
STE: Steroids
SUCRA: Surface Under the Cumulative Ranking curve
TAC: Tacrolimus
TSP: Tonsillectomy with steroid pulse therapy
