# Supplementary material for: Efficacy and safety of agents for IgA nephropathy: a network meta-analysis of randomized controlled trials
Source: Front Med (Lausanne). 2025 Jun 18;12:1515723. doi: 10.3389/fmed.2025.1515723 (PMC12213760; doi:10.3389/fmed.2025.1515723)
Supplement: Supplementary file 1 [file Data_Sheet_1.docx]

SUPPLEMENTARY MATERIALS

**Efficacy and safety of agents for IgA nephropathy: a network meta-analysis of randomized controlled trials**

Bo Chen^1,2,#^, Yan Zhu^1,#^, Yang Yang^1^, Gaosi Xu^1^*

^1^Department of Nephrology, the Second Affiliated Hospital, Jiangxi Medical College, Nanchang University

^2^Jiangxi Key Laboratory of Molecular Medicine, The Second Affiliated Hospital of Nanchang University

^#^These authors contributed equally to this work.

*Corresponding author.

**Professor Gaosi Xu (Ph.D. & M.D.)**

Address: No. 1, Minde Road, Nanchang, 330006, P.R. China.

E-mail: [gaosixu@163.com](mailto:gaosixu@163.com).

Tel: +86(0)791 86312770.

Fax: +86(0)791 86312770.

**List of Supplementary Materials**

S1: Table 1 PRISMA 2020 checklist

S2: Table 2 Characteristics of RCTs involved in the study

S3: Table 3 The selection criteria with a “PICOS” structure for the enrolled studies

S4: Table 4 SUCRA of the effects of various agents

S4-A SUCRA of the effects of various agents on adverse events

S4-B SUCRA of the effects of various agents on clinical remission

S4-C SUCRA of the effects of various agents on ESRD or KD

S4-D SUCRA of the effects of various agents on 24-h UPE

S5: Table 5 Comparison between inconsistency model and consistency model

Supplementary Figure 1: Risk of bias of included studies

Supplementary Figure 1-A: Risk of bias graph

Supplementary Figure 1-B: Risk of bias summary

Supplementary Figure 2: Forest plot of different drugs

Supplementary Figure 2-A: adverse events

Supplementary Figure 2-B: clinical remission

Supplementary Figure 2-C: ESRD or KD

Supplementary Figure 2-D: 24-h UPE

Supplementary Figure 3: Diagnostics and trace plots

Supplementary Figure 3-a: Diagnostic plot for adverse events

Supplementary Figure 3-b: Diagnostic plot for clinical remission

Supplementary Figure 3-c: Diagnostic plot for ESRD or KD

Supplementary Figure 3-d: Diagnostic plot for 24-h UPE

Supplementary Figure 3-A: Trace plots for adverse events

Supplementary Figure 3-B: Trace plots for clinical remission

Supplementary Figure 3-C: Trace plots for ESRD or KD

Supplementary Figure 3-D: Trace plots for 24-h UPE

Supplementary Figure 4: Heterogeneity analysis on end points

Supplementary Figure 4-A: adverse events

Supplementary Figure 4-B: clinical remission

Supplementary Figure 4-C: ESRD or KD

Supplementary Figure 4-D: 24-h UPE

Supplementary Figure 5: Inconsistency using node-splitting approach

Supplementary Figure 5-A: adverse events

Supplementary Figure 5-B: clinical remission

Supplementary Figure 5-C: ESRD or KD

Supplementary Figure 5-D: 24-h UPE

Supplementary Figure 6: Publication bias of funnel plot

Supplementary Figure 6-A: adverse events

Supplementary Figure 6-B: clinical remission

Supplementary Figure 6-C: ESRD or KD

Supplementary Figure 6-D: 24-h UPE

Supplementary Figure 7: Result of sensitivity analysis

Supplementary Figure 7-A: adverse events

Supplementary Figure 7-B: clinical remission

Supplementary Figure 7-C: ESRD or KD

Supplementary Figure 7-D: 24-h UPE

S6: Table 6 Results from pairwise meta-analyses

S6-A adverse events

S6-B clinical remission

S6-C ESRD or KD

S6-D 24-h UPE

S7: Table 7 Evaluation of meta-regression

S7-A adverse events

S7-B clinical remission

S7-C ESRD or KD

S7-D 24-h UPE

Supplementary Figure 8-a: Plot of SUCRA values coordinates combining CR and 24-h UPE.

Supplementary Figure 8-b: Plot of SUCRA values coordinates combining ESRD or KD and 24-hour AEs.

S8: Table 8 SUCRA value of adverse events, clinical remission, ESRD or KD and 24-h UPE

**S1: Table 1 PRISMA 2020 checklist**

| **Section and Topic** | **Item #** | **Checklist item** | **Location where item is reported** |
| --- | --- | --- | --- |
| **TITLE** | | |  |
| Title | 1 | Identify the report as a systematic review. | 1 |
| **ABSTRACT** | | |  |
| Abstract | 2 | See the PRISMA 2020 for Abstracts checklist. | 2-3 |
| **INTRODUCTION** | | |  |
| Rationale | 3 | Describe the rationale for the review in the context of existing knowledge. | 4-5 |
| Objectives | 4 | Provide an explicit statement of the objective(s) or question(s) the review addresses. | 5 |
| **METHODS** | | |  |
| Eligibility criteria | 5 | Specify the inclusion and exclusion criteria for the review and how studies were grouped for the syntheses. | 6-7 |
| Information sources | 6 | Specify all databases, registers, websites, organisations, reference lists and other sources searched or consulted to identify studies. Specify the date when each source was last searched or consulted. | 6 |
| Search strategy | 7 | Present the full search strategies for all databases, registers and websites, including any filters and limits used. | - |
| Selection process | 8 | Specify the methods used to decide whether a study met the inclusion criteria of the review, including how many reviewers screened each record and each report retrieved, whether they worked independently, and if applicable, details of automation tools used in the process. | 7-8 |
| Data collection process | 9 | Specify the methods used to collect data from reports, including how many reviewers collected data from each report, whether they worked independently, any processes for obtaining or confirming data from study investigators, and if applicable, details of automation tools used in the process. | 7-8 |
| Data items | 10a | List and define all outcomes for which data were sought. Specify whether all results that were compatible with each outcome domain in each study were sought (e.g. for all measures, time points, analyses), and if not, the methods used to decide which results to collect. | 7-8 |
|  | 10b | List and define all other variables for which data were sought (e.g. participant and intervention characteristics, funding sources). Describe any assumptions made about any missing or unclear information. | 10 |
| Study risk of bias assessment | 11 | Specify the methods used to assess risk of bias in the included studies, including details of the tool(s) used, how many reviewers assessed each study and whether they worked independently, and if applicable, details of automation tools used in the process. | 10 |
| Effect measures | 12 | Specify for each outcome the effect measure(s) (e.g. risk ratio, mean difference) used in the synthesis or presentation of results. | 8 (RR, SMD) |
| Synthesis methods | 13a | Describe the processes used to decide which studies were eligible for each synthesis (e.g. tabulating the study intervention characteristics and comparing against the planned groups for each synthesis (item #5)). | 10 |
|  | 13b | Describe any methods required to prepare the data for presentation or synthesis, such as handling of missing summary statistics, or data conversions. | 8-9 |
|  | 13c | Describe any methods used to tabulate or visually display results of individual studies and syntheses. | 8-9,11 |
|  | 13d | Describe any methods used to synthesize results and provide a rationale for the choice(s). If meta-analysis was performed, describe the model(s), method(s) to identify the presence and extent of statistical heterogeneity, and software package(s) used. | 8-9 |
|  | 13e | Describe any methods used to explore possible causes of heterogeneity among study results (e.g. subgroup analysis, meta-regression). | 8-9 |
|  | 13f | Describe any sensitivity analyses conducted to assess robustness of the synthesized results. | 8-9 |
| Reporting bias assessment | 14 | Describe any methods used to assess risk of bias due to missing results in a synthesis (arising from reporting biases). | 8-9 |
| Certainty assessment | 15 | Describe any methods used to assess certainty (or confidence) in the body of evidence for an outcome. | 9 |
| **RESULTS** | | |  |
| Study selection | 16a | Describe the results of the search and selection process, from the number of records identified in the search to the number of studies included in the review, ideally using a flow diagram. | 6 and Figure 1 |
|  | 16b | Cite studies that might appear to meet the inclusion criteria, but which were excluded, and explain why they were excluded. | 7 and Figure 1 |
| Study characteristics | 17 | Cite each included study and present its characteristics. | 10 and S2 |
| Risk of bias in studies | 18 | Present assessments of risk of bias for each included study. | 7-8 and Supplementary Figure 1 |
| Results of individual studies | 19 | For all outcomes, present, for each study: (a) summary statistics for each group (where appropriate) and (b) an effect estimate and its precision (e.g. confidence/credible interval), ideally using structured tables or plots. | 10, and  Figure 3,4 |
| Results of syntheses | 20a | For each synthesis, briefly summarise the characteristics and risk of bias among contributing studies. | 8-9 |
|  | 20b | Present results of all statistical syntheses conducted. If meta-analysis was done, present for each the summary estimate and its precision (e.g. confidence/credible interval) and measures of statistical heterogeneity. If comparing groups, describe the direction of the effect. | 11-15 and  Figure 2,3,4,5,6 |
|  | 20c | Present results of all investigations of possible causes of heterogeneity among study results. | 15, and S7 |
|  | 20d | Present results of all sensitivity analyses conducted to assess the robustness of the synthesized results. | 15, and Supplementary Figure 7 |
| Reporting biases | 21 | Present assessments of risk of bias due to missing results (arising from reporting biases) for each synthesis assessed. | 14, Supplementary Figure 6 |
| Certainty of evidence | 22 | Present assessments of certainty (or confidence) in the body of evidence for each outcome assessed. | - |
| **DISCUSSION** | | |  |
| Discussion | 23a | Provide a general interpretation of the results in the context of other evidence. | 15-19 |
|  | 23b | Discuss any limitations of the evidence included in the review. | 18, 19 |
|  | 23c | Discuss any limitations of the review processes used. | 19 |
|  | 23d | Discuss implications of the results for practice, policy, and future research. | 19 |
| **OTHER INFORMATION** | | |  |
| Registration and protocol | 24a | Provide registration information for the review, including register name and registration number, or state that the review was not registered. | 6 |
|  | 24b | Indicate where the review protocol can be accessed, or state that a protocol was not prepared. | - |
|  | 24c | Describe and explain any amendments to information provided at registration or in the protocol. | - |
| Support | 25 | Describe sources of financial or non-financial support for the review, and the role of the funders or sponsors in the review. | 21-22 |
| Competing interests | 26 | Declare any competing interests of review authors. | 22 |
| Availability of data, code and other materials | 27 | Report which of the following are publicly available and where they can be found: template data collection forms; data extracted from included studies; data used for all analyses; analytic code; any other materials used in the review. | - |

**S2: Table 2 Characteristics of RCTs involved in the study**

| **Author** | **Country** | **Patients** | **average age** | **Renal function** | **Proteinuria UPE(g/d)** | **Sample size(T/C)** | **Treatment** | **Control** | **Follow up** | **Outcome** |
| --- | --- | --- | --- | --- | --- | --- | --- | --- | --- | --- |
| Pozzi 1999 | Italy | UPE 1.0-3.5 g/d, Scr ≤ 1.5 mg/dL | T: 38  C: 40 | Ccr (mL/min) T: 93 (70-111) C: 87 (72-112) | T: 2.0(1.6-2.4) C: 1.8(1.4-2.4) | 86(43/43) | STE | Placebo | 72 | KD, AEs |
| Locatelli 2001 | Italy | UPE 1.0-3.5 g/d, Scr ≤ 1.5 mg/dL | NA | NA | T: 2.0 ± 0.60  C: 1.9 ± 0.70 | 86(43/43) | STE | Placebo | 72 | 24-h UPE |
| Chen 2002 | China | UPE > 2.0 g/d Scr < 4.0 mg/dL | 28 ± 10 | NA | T: 3.2 ± 1.7  C: 2.9 ± 1.5 | 62(31/31) | MMF | STE | 24 | CR, AEs |
| Praga 2003 | Spain | UPE > 0.5 g/d, Scr ≤ 1.5 mg/dL | T: 28 ± 12  C: 30 ± 12 | Scr (mg/dL) T: 1.0 ± 0.2 C: 0.9 ± 0.2 | T: 2.0 ± 1.3 C: 1.7 ± 0.8 | 44(23/21) | RASI | Placebo | 120 | KD, 24-h UPE |
| Maes 2004 | Belgium | UPE > 1g/d，20 < eGFR < 70 mL/min/1.73 m^2^ | >18 | Scr (mg/dL) T: 1.46 ± 0.08 C: 1.39 ± 0.10 | T: 1.9 ± 0.3 C: 1.3 ± 0.4 | 34(21/13) | MMF | Placebo | 36 | KD, 24-h UPE, AEs |
| Pozzi 2004 | Italy | UPE 1-3.5 g/d, Scr ≤ 1.5 mg/dL | NA | Scr (mg/dL)  T: 1.10(0.90-1.30)  C: 0.98(0.81-1.27) | T: 2.0(1.6-2.4) C: 1.8(1.4-2.4) | 86(43/43) | STE | RASI | 120 | CR |
| Frisch 2005 | USA | UPE > 1g/d，20 < eGFR < 80 mL/min/1.73 m^2^ | 18-75 | Scr (mg/dL) T: 2.6(1.2) C: 2.2(0.72) | T: 2.7(1.6) C: 2.7(1.4) | 32(17/15) | MMF | Placebo | 12 | KD, CR, 24-h UPE, AEs |
| Tang 2005 | China | UPE ≥ 1g/d, Scr ≤ 3.5 mg/dL | T: 42 ± 3 C: 43 ± 3 | Scr (mg/dL) T: 1.53 ± 0.17 C: 1.65 ± 0.23 | T: 1.80 ± 0.21 C: 1.87 ± 0.28 | 40(20/20) | MMF | Placebo | 18 | CR, 24-h UPE |
| Li 2006 | China | UPE > 1g/d, Scr < 2.8 mg/dL | ≥18 | Scr (mg/dL) T:1.11 ± 0.48 C:1.29 ± 0.54 | T: 1.80 ± 1.24 C: 2.35 ± 1.71 | 109(54/55) | RASI | Placebo | 26 | KD, 24-h UPE, AEs |
| Lou 2006 | China | UPE 1-3 g/d, Scr < 3 mg/dL | 18-65 | GFR (mL/min) T: 77.1 ± 23.7 C: 67.0 ± 28.2 | T: 1.66 ± 0.42 C: 2.04 ± 0.64 | 46(24/22) | LEF | RASI | 6 | CR, AEs |
| Horita 2007 | Japan | UPE 1-2.6 g/d, Ccr > 50 mL/min/1.73m^2^ | 33 ± 11 | Scr (mg/dL) T: 0.8 ± 0.2 C: 0.7 ± 0.1 | T: 1.6 ± 0.6 C: 1.6 ± 0.4 | 38(20/18) | STE+RASI | STE | 24 | ESRD, CR, 24-h UPE |
| Woo 2007 | Singapore | UPE > 1g/d, Scr > 1.6 mg/dL | T: 36 ± 11 C: 34 ± 11 | Scr (mg/dL) T: 1.6 ± 0.4 C: 1.5 ± 0.4 | T: 2.1 ± 0.8 C: 2.3 ± 1.6 | 75(37/38) | RASI | Placebo | 60 | ESRD, 24-h UPE |
| Xie 2011 | China | UPE 0.5-5 g/d, Scr < 3 mg/dL | 14-70 | GFR (mL/min) T1: 95.63 ± 28.31 T2: 91.50 ± 29.83 C: 97.85 ± 32.87 | T1: 1.35 ± 0.74 T2: 1.21 ± 0.56 C: 1.12 ± 0.54 | 99(35/34/30) | MZR/MZR+RASI | RASI | 12 | AEs |
| Lv 2009 | China | UPE 1-5 g/d, eGFR > 30 mL/min/1.73 m^2^ | 18-65 | Scr (mg/dL) T: 1.1 ± 0.3 C: 1.1 ± 0.3 | T: 2.5 ± 0.9 C: 2.0 ± 0.8 | 63(33/30) | STE+RASI | RASI | 48 | KD, CR,  24-h UPE, AEs |
| Liu 2010 | China | UPE >3.5 g/d Scr < 5 mg/dL | 16-61 | Scr (mg/dL) T: 1.09 ± 0.27 C: 1.04 ± 0.29 | T: 4.82 ± 2.63 C: 4.91 ± 2.42 | 40(20/20) | STE+LEF | STE+MMF | 6 | CR, 24-h UPE, AEs |
| Pozzi 2010 | Italy | UPE ≥ 1.0 g/d, Scr ≤ 2.0 mg/dL | T: 34  C: 40 | GFR (mL/min) T: 72 (53-88) C: 63 (44-85) | T: 2.1(1.5-3.5) C: 2.0(1.5-2.7) | 207(101/10) | STE+AZA | STE | 84 | ESRD, AEs |
| Tang 2010 | China | UPE ≥ 1g/d, Scr ≤ 3.5 mg/dL | T: 42 ± 3 C: 43 ± 3 | GFR (mL/min) T: 52.5 ± 4.40 C: 50.0 ± 4.51 | T: 1.80 ± 0.21 C: 1.87 ± 0.28 | 40(20/20) | MMF | Placebo | 72 | ESRD, AEs |
| Stangou 2011 | Japan | UPE ≥ 1 g/d,eGFR ≥ 30 mL/min/1.73 m^2^ | T: 47 ± 12 C: 51 ± 9 | GFR (mL/min) T: 57.4 ± 28.7 C: 52.0 ± 26.7 | T: 2.4 ± 1.0 C: 2.4 ± 0.9 | 22(12/10) | STE+AZA | STE | T: 48 C: 56 | 24-h UPE, AEs |
| Kim 2013 | Korea | 0.3 ≤ UACR< 3.0 g/g creatinine, eGFR ≥ 45 mL/min/1.73 m^2^ | 18-70 | Scr (mg/dL) T: 1.06 ± 0.30 C: 0.98 ± 0.26 | T: 1.0 ± 0.6 C: 0.9 ± 0.4 | 40(20/20) | TAC | Placebo | 4 | 24-h UPE, AEs |
| Pozzi 2013 | Italy | UPE ≥1.0 g/d, Scr > 2.0 mg/dL | T: 43 C: 37 | Scr (mg/dl) T: 2.06(2.37-3.04) C: 2.85(2.38-3.55) | T: 3.20(1.74-5.54)  C: 2.00(1.50-3.23) | 46(20/26) | STE+AZA | STE | 72 | ESRD, AEs, 24-h UPE |
| Kawamura 2014 | Japan | UPE 1.0-3.5 g/d, Scr ≤ 1.5 mg/dL | 10-69 | GFR (mL/min) T: 75(24) C: 69 (22) | T: 1.6(0.5) C: 1.6(0.6) | 72(33/39) | TSP | SP | 12 | CR |
| Liu 2014 | China | UPE > 1g/d,eGFR > 30 mL/min/1.73 m^2^ | 18-69 | Scr (mg/dL) T: 1.02 ± 0.28 C: 1.01 ± 0.26 | T: 3.17 ± 3.25 C: 2.60 ± 2.03 | 48(23/25) | STE+CsA | STE | 12 | ESRD, CR, 24-h UPE, AEs |
| Masutani 2016 | Japan | UPE > 0.5g/d | T: 44 ± 11 C: 36 ± 13 | Scr (mg/dL) T: 0.96 ± 0.35 C: 0.90 ± 0.38 | T: 1.01(0.50-1.85) C: 0.98(0.56-1.91) | 40(20/20) | SP+MZR | SP | 25 | CR, 24-h UPE, AEs |
| Kaneko 2015 | Japan | UPE > 0.5g/d, eGFR > 20 mL/min/1.73 m^2^ | T: 38 ± 13 C: 35 ± 14 | Scr (mg/dL) T: 0.93 ± 0.38 C: 0.94 ± 0.37 | T: 1.48 ± 0.94 C: 1.27 ± 0.86 | 62(31/31) | TSP+MZR | TSP | 12 | 24-h UPE, CR |
| Rauen 2015 | Germany | UPE 0.75-3.5 g/d, eGFR >30 mL/min/1.73 m^2^ | T: 46 ± 13 C: 43 ± 13 | Scr (mg/dL) T: 1.6 ± 0.6 C: 1.6 ± 0.7 | T: 1.6 ± 0.7 C: 1.8 ± 0.8 | 162(80/82) | Supportive Care | Supportive Care plus  Immunosuppression | 36 | CR, AEs |
| Lafayette 2017 | USA | UPE >1g/d,eGFR < 90 mL/min/1.73 m^2^ | 40 (21–63) | Scr (mg/dL) T: 1.7(0.8-2.3) C: 1.3(0.8-2.4) | T: 2.6(0.9-5.3) C: 1.7(0.6-4.0) | 34(17/17) | RIT | Placebo | 12 | 24-h UPE, CR |
| Yang 2016 | China | UPE 1.0-3.5 g/d, Scr ≤ 1.5 mg/dL | 18-69 | Scr (μmol/L)) T: 81.33 ± 19.45 C: 92.82 ± 15.03 | T: 0.94 ± 1.03 C: 1.36 ± 1.05 | 98(49/49) | Tonsillectomy +Tripterygium or STE | Tripterygium or STE | 48 | CR |
| Hirai 2017 | Japan | UPE > 0.5 g/d | >16 | GFR (mL/min) T: 67.5 ± 30.4 C: 67.4 ± 33.6 | T:0.90 ± 0.82 C:1.11 ± 1.36 | 42(21/21) | MZR + standard  treatment | standard  treatment | 36 | ESRD, CR |
| Hou 2017 | China | UPE ≥ 1.0 g/d, eGFR ≥ 30 mL/min/1.73 m^2^ | 18-65 | GFR (mL/min)  T: 90.2(64.4-109.6)  C: 94.3(72.2-111.4) | T: 2.37 ± 1.23 C: 2.47 ± 2.01 | 174(86/88) | MMF+STE | STE 0.8 to 1.0 mg/kg/d (Full-Dose) | 12 | ESRD, AEs |
| Rauen 2018 | Germany | UPE ≥ 0.75 g/d, eGFR ≥ 60 mL/min/1.73 m^2^ | T: 46 ± 12 C: 42 ± 13 | GFR (mL/min) T: 88.2 ± 28.6 C: 94.2 ± 32.2 | T: 1.6±0.7 C: 1.6±0.8 | 109(54/55) | Supportive Care | STE | 36 | 24-h UPE, ESRD, AEs |
| Kohagura 2018 | Japan | UPE ≥ 0.5 g/d, Scr ≤ 1.5 mg/dL | 15-70 | GFR (mL/min) T: 85 (29) C: 84 (24) | T: 0.9(0.7-1.2) C: 1.0(0.6-1.5) | 77(37/40) | TSP | TSP+RASI | 24 | CR |
| Liu 2019 | China | UPE 0.75-3.5 g/d, eGFR > 30 mL/min/1.73 m^2^ | 18-75 | GFR (mL/min) T: 52.1 ± 19.7 C: 55.5 ± 18.7 | T: 1.6(1.1, 2.2) C: 1.9(1.3, 2.6) | 60(30/30) | RASI+HCQ | RASI | 6 | CR, 24-h UPE, KD, AEs |
| Ni 2021 | China | UPE > 1.0 g/d, eGFR < 60 mL/min/1.73 m^2^ | 18-65 | GFR (mL/min) T: 83.1 ± 39.6 C: 84.6 ± 38.5 | T: 1.8(1.3-3.5) C: 1.9(1.2-2.9) | 108(59/49) | STE（low-dose）+LEF | STE | 24 | AEs, 24-h UPE |
| Barratt 2022 | UK | UPE > 0.75 g/d, eGFR > 25 mL/min/1.73 m^2^ | >18 | GFR (mL/min) T: 55 (52, 92) C: 49 (48, 54) | T: 1.4(1.3, 1.7) C: 1.6(1.5, 1.6) | 10(5/5) | Atacicept 75 mg | Placebo | 18 | 24-h UPE, AEs |
| Han 2022 | Korea | UPCR ≥ 0.75mg/mgC, eGFR 20-50 mL/min/1.73 m^2^ | 19-65 | GFR (mL/min) T: 36.3 ± 9.4 C: 33.0 ± 7.7 | T: 1.7 ± 0.6 C: 2.2 ± 1.0 | 48(26/22) | STE+MMF | Placebo | 12 | CR, 24-h UPE, AEs |
| Lv 2022 | China | UPE > 0.75 g/d, eGFR 20-120 mL/min/1.73 m^2^ | T:36 C:37 | GFR (mL/min) T:56.1(43.2-75.0) C:59.0(42.0-77.6) | T: 1.99(1.36-3.09) C: 1.93(1.38-2.88) | 503(257/246) | STE | Placebo | 72 | KD, 24-h UPE, AEs |
| Heerspink 2023 | Australia | UPE > 0.75 g/d, eGFR ≥ 30 mL/min/1.73 m^2^ | ≥18 | GFR (mL/min) T:56.9(24.4) C:57.1(23.6) | T:1.8(1.2–2.8) C:1.8(1.3–2.6) | 404(202/202) | Sparsentan | RASI | 9 | ESRD, CR, AEs |
| Hou 2023 | China | UPE 0.75-3.5 g/d, eGFR 30-60 mL/min/1.73 m^2^ | 18-70 | GFR (mL/min) T: 50.9(18.2) C: 49.3(17.7) | T:2.1(1.9) C:1.7(1.3) | 170(85/85) | MMF | Placebo | 36 | KD, AEs |
| Lafayette 2023 | USA | UPE ≥1 g/d, eGFR 35-90 mL/min/1.73 m2 | ≥18 | GFR (mL/min) T: 56 (45-71) C: 55 (46-68) | T:2.71(1.73) C:2.71(2.20) | 364(182/182) | Nefecon 16mg/d | Placebo | 15 | KD, AEs |
| Lv 2023 | China | UPE ≥ 0.75 g/d, eGFR > 35 mL/min/1.73 m^2^ | T: 37(9) C: 38(7) | GFR (mL/min) T: 75.7 ± 6.4 C: 85.0 ± 6.7 | T: 1.6 ± 0.2 C: 1.9 ± 0.2 | 28(14/14) | Telitacicept 240mg | Placebo | 6 | 24-h UPE, AEs |
| Lai 1987 | China | UPE ≥1.5 g/d, Ccr > 50 mL/min/1.73 m^2^ | 24-58 | Ccr(mL/min) T: 73.1(6.5) C: 71.7(3.3) | T: 4.2(1.1) C: 2.5(0.3) | 19(9/10) | CsA 5mg/kg/day | Placebo | 3 | CR, AEs |
| Mathur 2024 | UK | UPE ≥ 1 g/d, eGFR ≥ 30 mL/min/1.73 m^2^ | ≥18 | GFR (mL/min) T: 56.0(34.0–109.0) C: 68.5(33.0–116.0) | T: 1.90(0.76–12.44)  C: 2.13(0.76–8.48) | 76(38/38) | Sibeprenlimab 8mg/kg/mon | Placebo | 16 | CR, AEs |
| Zhang 2024 | China | UPE ≥ 0.75 g/d, eGFR ≥ 30 mL/min/1.73 m^2^ | 36-43 | GFR (mL/min) T: 57.9 (28.9) C: 65.7 (32.6) | T: 1.3 (1.0) C: 1.3 (0.6) | 51(26/25) | Iptacopan 200mg bid | Placebo | 6 | 24-h UPE, AEs |
| Sun 2023 | China | UPE ≥ 1 g/d, eGFR ≥ 45 mL/min/1.73 m^2^ | 18-55 | GFR (mL/min) T: 84.2 ± 32.2 C: 86.42±33.93 | T: 1.8 ± 0.7 C: 1.9 ± 0.4 | 142(71/71) | Fluticasone+RASI | RASI | 9 | 24-h UPE |
| David 2021 | UK | eGFR 25-75 mL/min/1.73 m^2^ | ≥18 | GFR (mL/min) T: 44.3 (12.4) C: 43.2 (12.0) | T: 0. 9(0.6-1.4) C: 0.9(0.5-1.6) | 270(137/133) | SGLT2i | Placebo | 25 | KD, AEs |
| Manno 2009 | NA | UPE ≥ 1.0 g/d, eGFR ≥ 50 mL/min/1.73 m^2^ | T: 32 ± 11 C: 35 ± 11 | GFR (mL/min) T: 100.4 ± 26.1 C: 97.5 ± 27.7 | T: 1.7(1.2–2.5) C: 1.5(1.4–2.3) | 97(48/49) | STE+RASI | RASI | 96 | KD |
| Coppo 2007 | Italy | UPE 1-3.5 g/d, CrcL ≥ 50 mL/min/1.73 m^2^ | 9-35 | Ccr (mL/min) T: 116.0 ± 24.3 C: 109.2 ± 18.0 | T: 1.61 ± 0.70 C: 1.87 ± 0.74 | 66(32/34) | RASI | Placebo | 38 | KD, CR, AEs |
| Yang 2018 | China | UPE 0.75-3.5 g/d, eGFR > 30 mL/min/1.73 m^2^ | T: 37 ± 9 C: 38 ± 11 | GFR (mL/min) T: 51.2 ± 21.7 C: 51.7 ± 18.9 | T: 1.5(1.2, 2.1) C: 1.5(1.2, 1.9) | 180(90/90) | RASI+HCQ | RASI | 6 | CR, 24-h UPE |
| Nakamura 2000 | Japan | UPE 1-3.0 g/d, Ccr > 80 mL/min/1.73 m^2^ | 18-54 | Ccr (mg/dL) T: 0.8 ± 0.2 C: 0.8 ± 0.2 | T: 1.7 ± 0.7 C: 1.6 ± 0.6 | 16(8/8) | RASI | Placebo | 3 | 24-h UPE |
| Yoshikawa  2006 | Japan | NA | T: 12  C: 11 | Scr (mg/dL) T: 49(19) C: 43(14) | T: 1.29(1.19) C: 1.16(1.13) | 80(40/40) | STE+AZA | STE | 24 | CR, 24-h UPE, AEs |
| Ballardie 2002 | Britain | NA | 18-54 | NA | T: 3.9 ± 0.2 C: 4.6 ± 0.4 | 38(19/19) | STE+AZA | Placebo | 60 | ESRD, 24-h UPE, |
| Hogg 2015 | USA | eGFR > 40 mL/min/1.73 m^2^ | T: 32 ± 12 C: 32 ± 13 | GFR (mL/min) T: 95.3 ± 36.5 C: 105.6 ± 49.0 | T: 1.59 ± 0.90 C: 1.40 ± 0.56 | 52(25/27) | MMF | Placebo | 12 | CR, 24-h UPE |
| Horita 2004 | Japan | UPE 0.4-1.6 g/d, Ccr > 50 mL/min/1.73 m^2^ | T: 40 ± 10 C: 40 ± 11 | GFR (mL/min) T: 91.5 ± 24.6 C: 92.5 ± 17.2 | T: 0.75 ± 0.30 C: 0.73 ± 0.36 | 21(11/10) | RASI | Placebo | 6 | 24-h UPE |
| Katafuchi 2003 | Japan | NA | T: 34 ± 13 C: 33 ± 11 | Ccr (mg/dL) T: 90.8 ± 27.3 C: 90.5 ± 26.1 | T: 2.27 ± 2.19 C: 1.05 ± 0.83 | 100(43/47) | STE | Placebo | 60 | 24-h UPE |
| Kobayashi 1996 | Japan | UPE 1-2 g/d, Ccr > 70 mL/min/1.73m^2^ | T: 30 ± 7 C: 33 ± 10 | Ccr (mg/dL) T: 85 ± 14 C: 88 ± 13 | T: 1.4 ± 0.4 C: 1.3 ± 0.3 | 46(20/26) | STE | Placebo | 120 | 24-h UPE, CR, ESRD, AEs |
| Koike 2008 | Japan | NA | T: 38 ± 10 C: 38 ± 13 | Scr (mg/dL) T: 0.92±0.26 C: 1.15±0.35 | T: 0.97±0.75 C: 0.89±0.49 | 48(24/24) | STE | Placebo | 24 | 24-h UPE |
| Min 2017 | China | UPE ≥ 1.0 g/d, eGFR ≥ 30 mL/min/1.73 m^2^ | T: 37 ± 11 C: 7 ± 12 | GFR (mL/min) T: 84.10±25.55 C: 84.26±29.05 | T: 1.91(1.18, 2.88) C: 1.78(1.31, 3.49) | 85(40/45) | STE（low-dose）+LEF | STE | 88 | KD, 24-h UPE, AEs |

Abbreviations: UPE, urinary protein excretion; Scr, serum creatinine; T, treatment group; C, control group; Ccr, creatinine clearance rate; UPCR, urinary protein-creatinine ratio; Crcl, creatinine clearance; GFR, glomerular filtration rate; UACR, urine albumin creatine rate; eGFR, estimated glomerular filtration rate; AEs, adverse events; CR, clinical remission; ESRD, end-stage renal disease; 24-h UPE, 24-hour urinary protein excretion; KD, kidney dysfunction; TSP, tonsillectomy with steroid pulse therapy; SP, Steroid pulse; MMF, mycophenolate mofetil; STE, steroids; RASI, renin-angiotensin system inhibitors; LEF, leflunomide; CsA, Cyclosporin A; MZR, mizoribine; RIT, rituximab; HCQ, hydroxychloroquine; AZA, azathioprine; TAC, Tacrolimus; SGLT2i, sodium glucose cotransporter 2 inhibitor.

.

**S3: Table 3 The selection criteria with a “PICOS” structure for the enrolled studies**

| Items | Specific Criteria |
| --- | --- |
| Patients | Patients with biopsy proven IgAN and nephrotic range proteinuria (urinary protein excretion >0.5g/24h) and all included participants had a study-duration of at least 3 months. |
| Interventions/Comparisons | Interventions/Comparisons included TSP, MMF, STE, RASI, LEF, CsA, MZR, RIT, HCQ, AZA, TAC, SGLT2i, Iptacopan, Atacicept, Telitacicept, Sparsentan, Sibeprenlimab, Nefecon, Placebo |
| Outcomes | Outcomes were clinical remission (CR), ESRD or KD, 24 hours urinary protein excretion (24-h UPE) and adverse events (AEs). |
| Study designs | Studies were randomized controlled trials (RCTs) |

Abbreviations: IgAN, IgA nephropathy; AEs, adverse events; CR, clinical remission; ESRD, end-stage renal disease; and 24-h UPE, 24-hour urinary protein excretion; RCTs, Randomized controlled trials; TSP, tonsillectomy with steroid pulse therapy; MMF, mycophenolate mofetil; STE, steroids; RASI, renin-angiotensin system inhibitors; LEF, leflunomide; CsA, Cyclosporin A; MZR, mizoribine; RIT, rituximab; HCQ, hydroxychloroquine; AZA, azathioprine; TAC, Tacrolimus; SGLT2i, sodium glucose cotransporter 2 inhibitor.

**S4: Table 4 SUCRA of the effects of various agents**

S4-A SUCRA of the effects of various agents on adverse events

| **Treatment** | **SUCRA** | **PrBest** | **MeanRank** |
| --- | --- | --- | --- |
| **Iptacopan** | 88.4 | 56.1 | 3.1 |
| **SGLT2i** | 85.4 | 13.2 | 3.6 |
| **Atacicept** | 83.2 | 15.5 | 4 |
| **Telitacicept** | 70.1 | 4.8 | 6.4 |
| **Placebo** | 66.5 | 0.0 | 7 |
| **MZR** | 66.4 | 5.6 | 7 |
| **RASI** | 63.9 | 0.2 | 7.5 |
| **Sparsentan** | 59.9 | 1.6 | 8.2 |
| **CsA** | 56.3 | 0.7 | 8.9 |
| **Sibeprenlimab** | 55.9 | 0.2 | 8.9 |
| **MZR+RASI** | 45.2 | 1.1 | 10.9 |
| **AZA** | 40.4 | 0.0 | 11.7 |
| **STE+MMF** | 38.6 | 0.2 | 12.0 |
| **LEF** | 37.6 | 0.1 | 12.2 |
| **Nefecon** | 27.8 | 0.1 | 14.0 |
| **MMF** | 25.0 | 0.0 | 14.5 |
| **STE** | 24.6 | 0.0 | 14.6 |
| **HCQ** | 12.2 | 0.6 | 16.8 |
| **TAC** | 2.5 | 0.0 | 18.6 |

Abbreviations: MMF, mycophenolate mofetil; STE, steroids; RASI, renin-angiotensin system inhibitors; LEF, leflunomide; CsA, Cyclosporin A; MZR, mizoribine; HCQ, hydroxychloroquine; AZA, azathioprine; TAC, Tacrolimus; SGLT2i, sodium glucose cotransporter 2 inhibitor; SUCRA; surface under the cumulative ranking curve.

S4-B SUCRA of the effects of various agents on clinical remission

| **Treatment** | **SUCRA** | **PrBest** | **MeanRank** |
| --- | --- | --- | --- |
| **TSP** | 92.8 | 26.3 | 2 |
| **Sibeprenlimab** | 85.6 | 45.9 | 3 |
| **STE+RASI** | 79.4 | 0.1 | 3.9 |
| **STE+MMF** | 77.6 | 27.5 | 4.1 |
| **STE** | 73.6 | 0.0 | 4.7 |
| **Sparsentan** | 72.2 | 0.1 | 4.9 |
| **MMF** | 56.5 | 0.0 | 7.1 |
| **LEF** | 49.0 | 0.0 | 8.1 |
| **RASI** | 47.6 | 0.0 | 8.3 |
| **HCQ** | 35.6 | 0.0 | 10.0 |
| **AZA** | 23.6 | 0.0 | 11.7 |
| **CsA** | 18.5 | 0.0 | 12.4 |
| **RIT** | 18.3 | 0.0 | 12.4 |
| **MZR** | 11.7 | 0.0 | 13.4 |
| **Placebo** | 8.0 | 0.0 | 13.9 |

Abbreviations: TSP, tonsillectomy with steroid pulse therapy; MMF, mycophenolate mofetil; STE, steroids; RASI, renin-angiotensin system inhibitors; LEF, leflunomide; CsA, Cyclosporin A; MZR, mizoribine; RIT, rituximab; HCQ, hydroxychloroquine; AZA, azathioprine; SUCRA; surface under the cumulative ranking curve.

S4-C SUCRA of the effects of various agents on ESRD or KD

| **Treatment** | **SUCRA** | **PrBest** | **MeanRank** |
| --- | --- | --- | --- |
| **STE+RASI** | 98.1 | 86 | 1.2 |
| **Sparsentan** | 82.6 | 7.8 | 3.1 |
| **SGLT2i** | 68.7 | 2.1 | 4.8 |
| **RASI** | 67.9 | 0 | 4.8 |
| **STE** | 58.0 | 0.0 | 6.0 |
| **STE+AZA** | 56.5 | 0.1 | 6.2 |
| **LEF** | 49.7 | 0.8 | 7.0 |
| **Nefecon** | 46.7 | 0.1 | 7.4 |
| **MMF** | 41.5 | 0.0 | 8.0 |
| **MZR** | 35.8 | 2;6 | 8.7 |
| **Placebo** | 21.5 | 0.0 | 10.4 |
| **HCQ** | 11.6 | 0.3 | 11.6 |
| **CsA** | 11.2 | 0.2 | 11.7 |

Abbreviations: ESRD, end-

stage renal disease; MMF, mycophenolate mofetil; STE, steroids; RASI, renin-angiotensin system inhibitors; LEF, leflunomide; CsA, Cyclosporin A; MZR, mizoribine; HCQ, hydroxychloroquine; AZA, azathioprine; SGLT2i, sodium glucose cotransporter 2 inhibitor; SUCRA; surface under the cumulative ranking curve.

S4-D SUCRA of the effects of various agents on 24-h UPE

| **Treatment** | **SUCRA** | **PrBest** | **MeanRank** |
| --- | --- | --- | --- |
| **Telitacicept** | 99.9 | 98.7 | 1 |
| **STE+RASI** | 87.4 | 0.7 | 2.9 |
| **STE+MMF** | 57.5 | 0.2 | 7.4 |
| **LEF** | 57.1 | 0 | 7.4 |
| **STE+AZA** | 53.7 | 0.0 | 7.9 |
| **STE** | 53.6 | 0.0 | 8.6 |
| **Iptacopan** | 53.1 | 0.1 | 8.0 |
| **HCQ** | 53.1 | 0.0 | 8.0 |
| **RASI** | 52.1 | 0.0 | 8.2 |
| **Atacicept** | 49.4 | 0.2 | 8.6 |
| **MMF** | 48.1 | 0.0 | 8.8 |
| **CsA** | 36.3 | 0.0 | 10.6 |
| **TAC** | 34.2 | 0.0 | 10.9 |
| **RIT** | 27.0 | 0.0 | 11.9 |
| **MZR** | 19.0 | 0.0 | 13.2 |
| **Placebo** | 18.5 | 0.0 | 13.2 |

Abbreviations: 24-h UPE, 24-hours urinary protein excretion; MMF, mycophenolate mofetil; STE, steroids; RASI, renin-angiotensin system inhibitors; LEF, leflunomide; CsA, Cyclosporin A; MZR, mizoribine; RIT, rituximab; HCQ, hydroxychloroquine; AZA, azathioprine; CsA, Cyclosporin A; SUCRA, surface under the cumulative ranking curve.

| **S5: Table 5 Comparison between inconsistency model and consistency model** | | | | | |
| --- | --- | --- | --- | --- | --- |
| **Outcomes** | **Dbar** | **Data points** | **Pd** | **DIC** | **I^2^** |
| Clinical Remission (Consistency model) | 53.33 | 52 | 40.86 | 94.19 | 4% |
| Clinical Remission (Inconsistency model) | 50.12 | 52 | 42.90 | 93.01 | 0% |
| ESRD or KD (Consistency model) | 52.40 | 52 | 43.61 | 96.01 | 3% |
| ESRD or KD (Inconsistency model) | 51.58 | 52 | 44.52 | 96.10 | 1% |
| 24-h UPE (Consistency model) | 37.74 | 72 | 33.97 | 71.71 | 7% |
| 24-h UPE (Inconsistency model) | 37.44 | 72 | 34.60 | 72.04 | 7% |
| Adverse Events (Consistency model) | 82.22 | 73 | 62.04 | 144.25 | 12% |
| Adverse Events (Inconsistency model) | 80.06 | 73 | 66.04 | 146.10 | 10% |

**Supplementary Figure 1: Risk of bias of included studies**


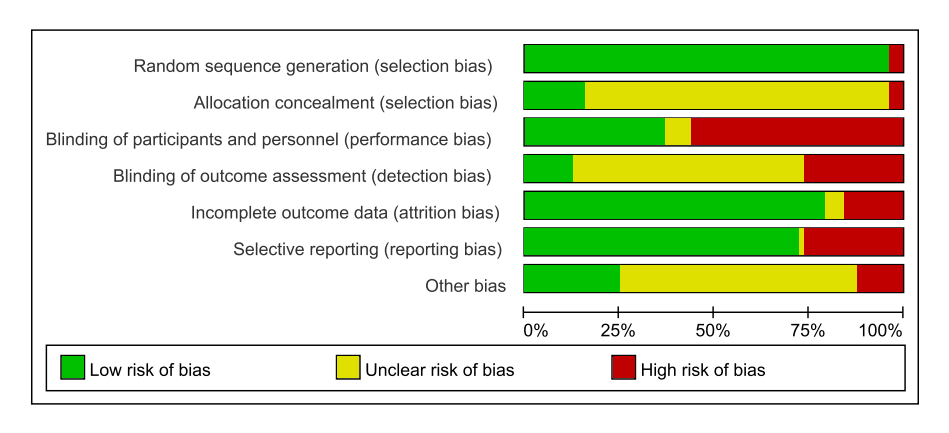


Supplementary Figure 1-A: Risk of bias graph


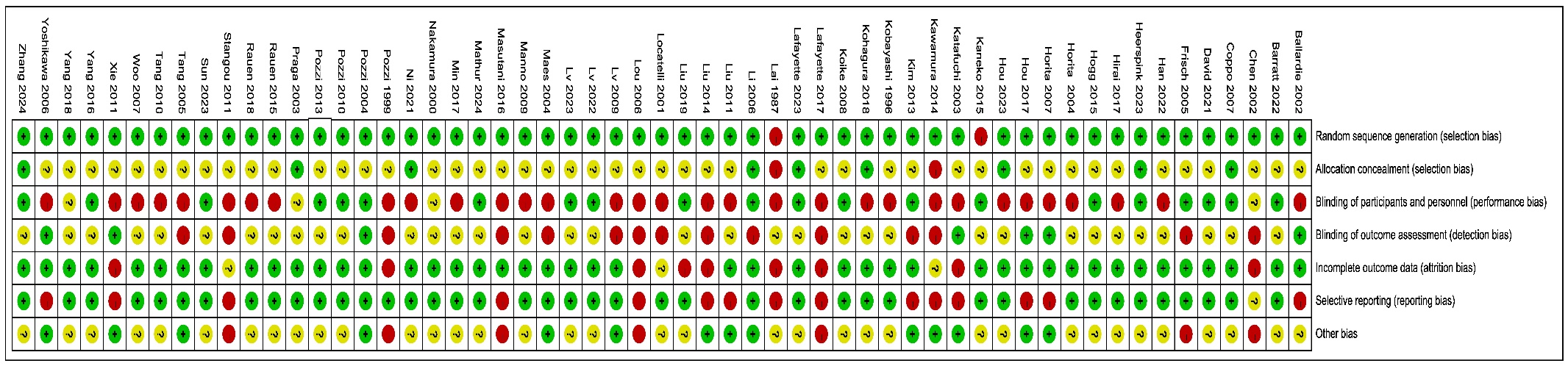


Supplementary Figure 1-B: Risk of bias summary

**Supplementary Figure 2: Forest plot of different drugs**


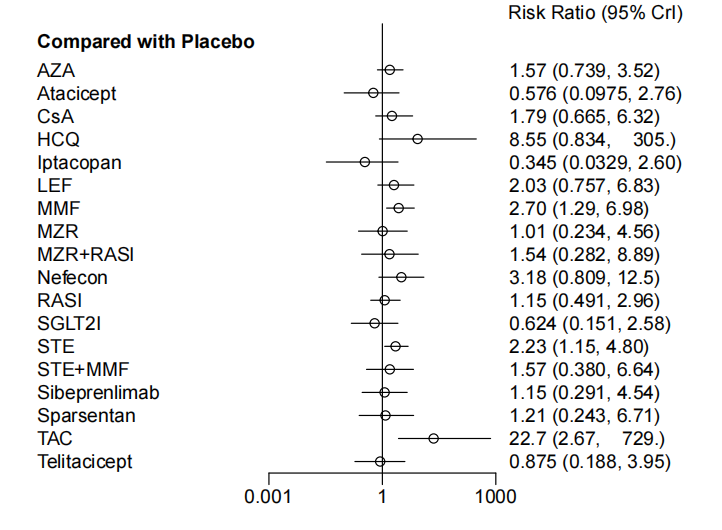


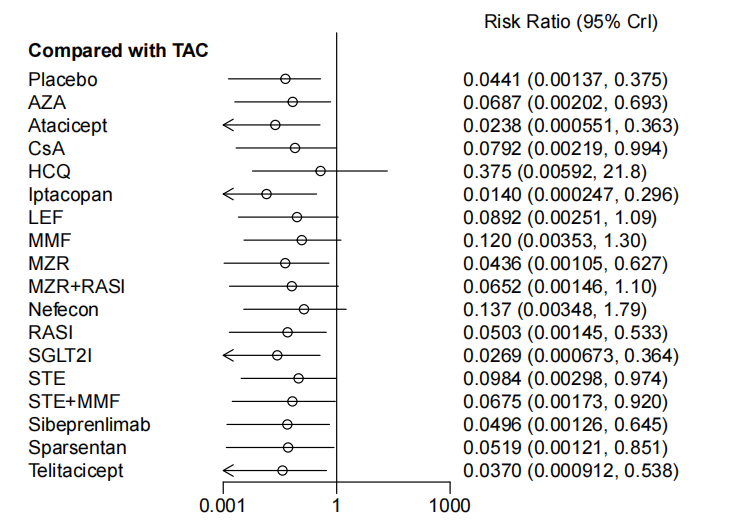


Supplementary Figure 2-A: adverse events


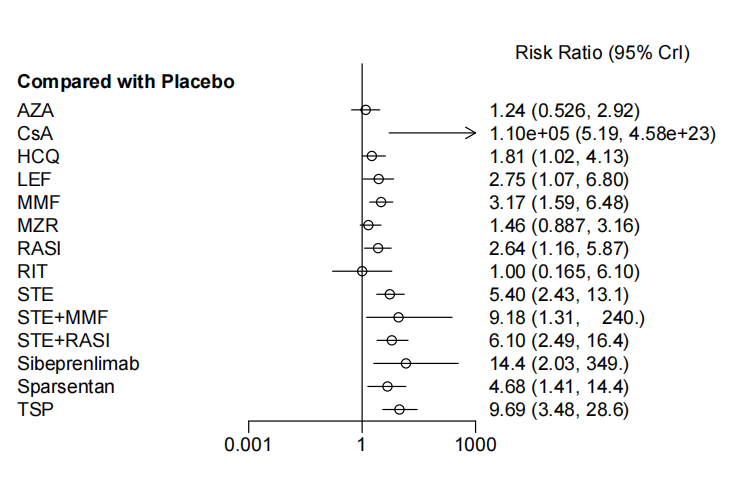


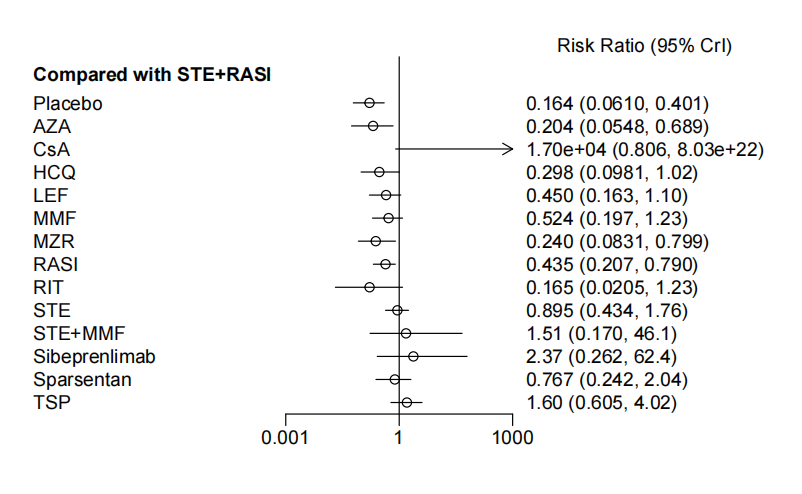


Supplementary Figure 2-B: clinical remission


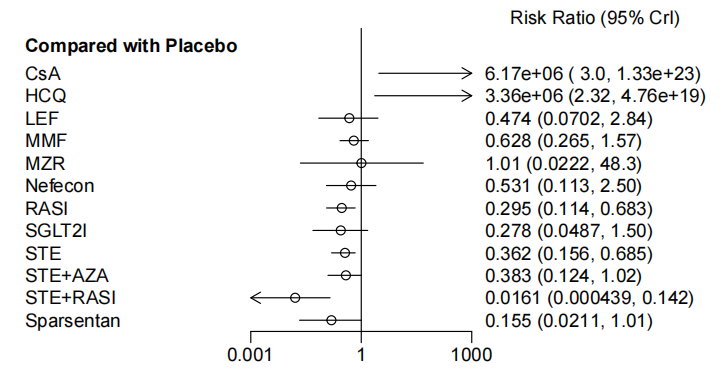


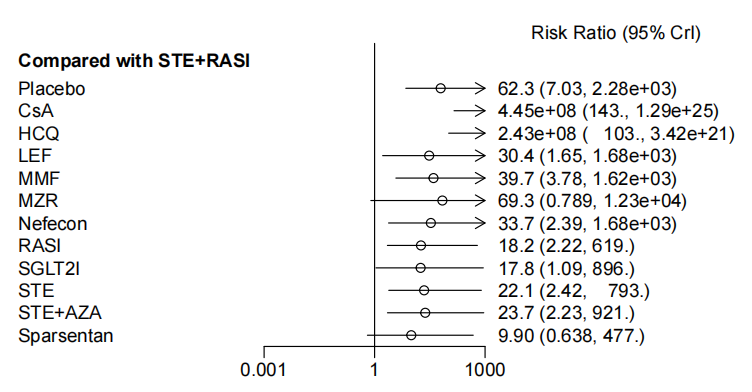


Supplementary Figure 2-C: ESRD or KD


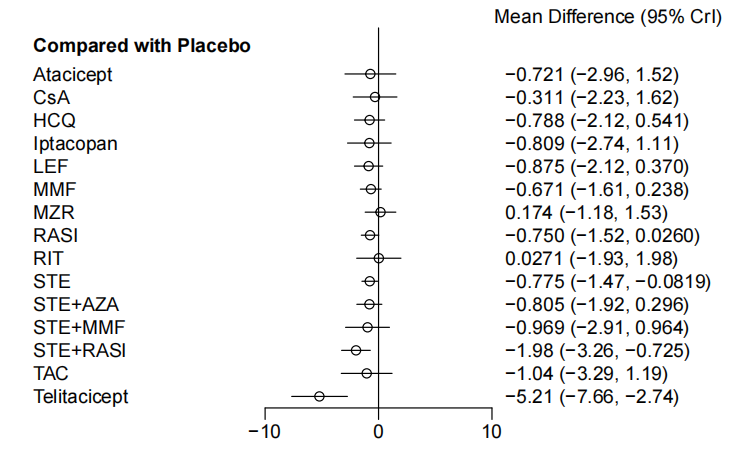


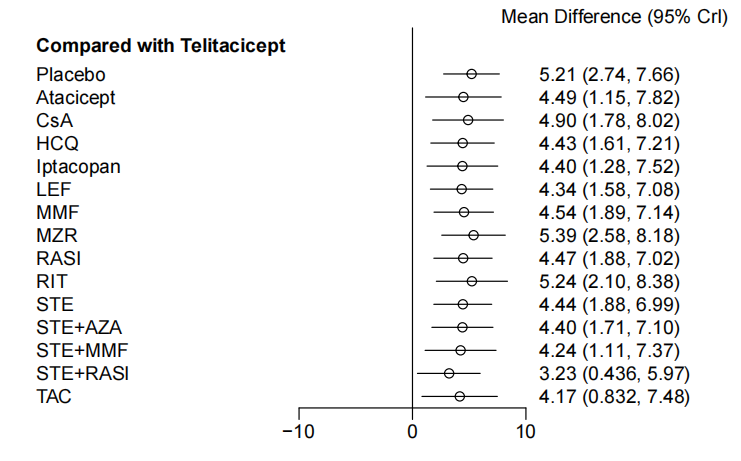


Supplementary Figure 2-D: 24-h UPE

**Supplementary Figure 3: Diagnostics and trace plots**


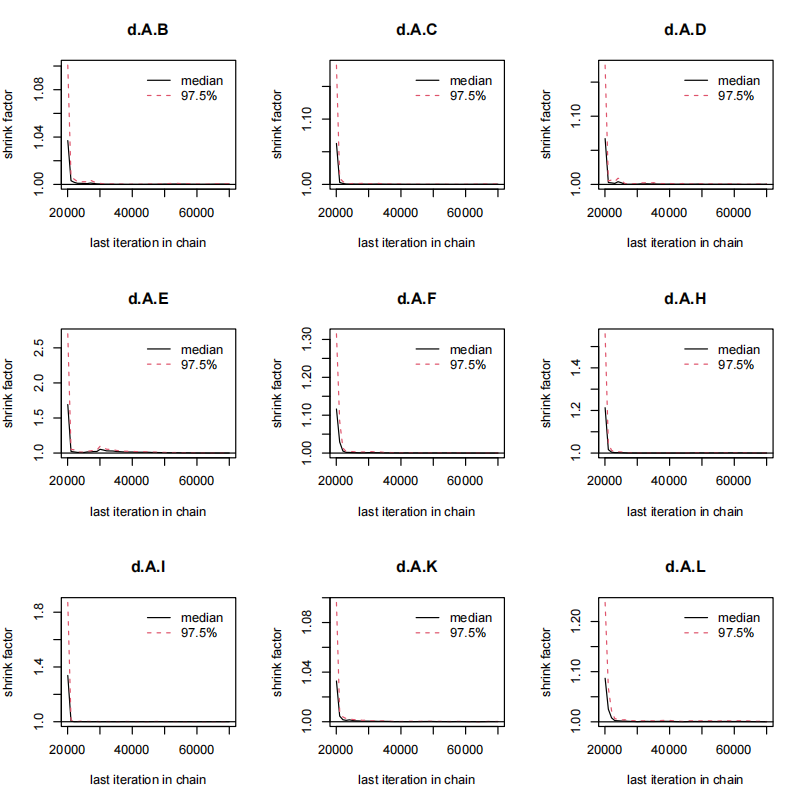


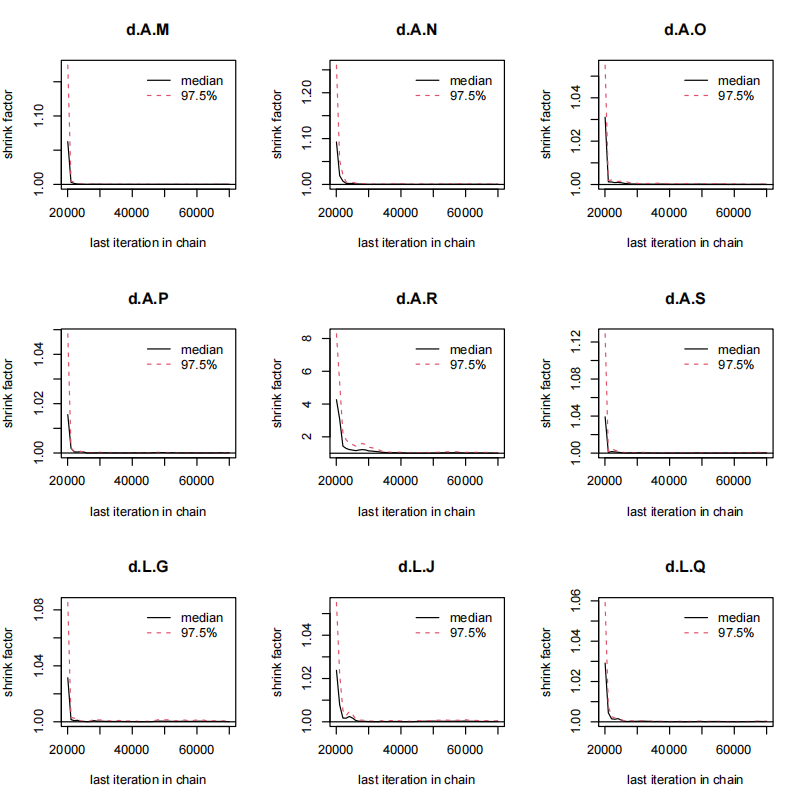


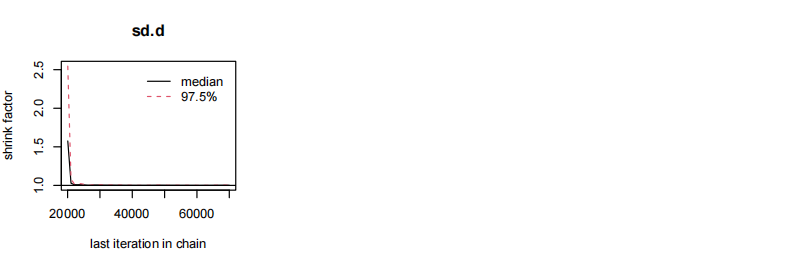


Supplementary Figure 3-a: Diagnostic plot for adverse events

Abbreviations: A, placebo; B, AZA; C, Atacicept; D, CsA; E, HCQ; F, Iptacopan; G, LEF; H, MMF; I, MZR; J, MZR+RASI; K, Nefecon; L, RASI; M, SGLT2I; N, STE; O, STE+MMF; P, Sibeprenlimab; Q, Sparsentan; R, TAC; S, Telitacicept.


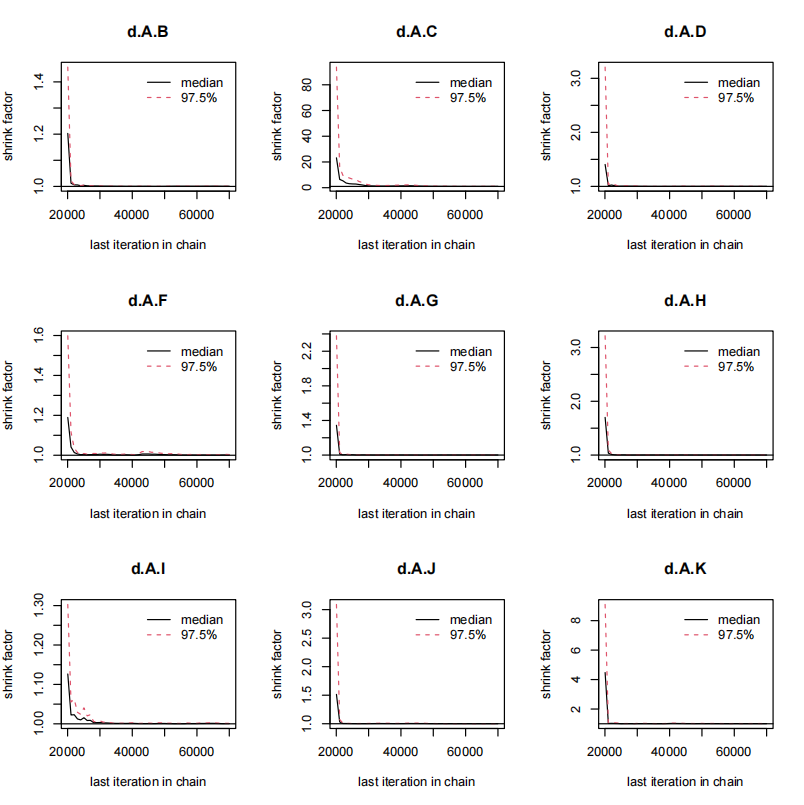


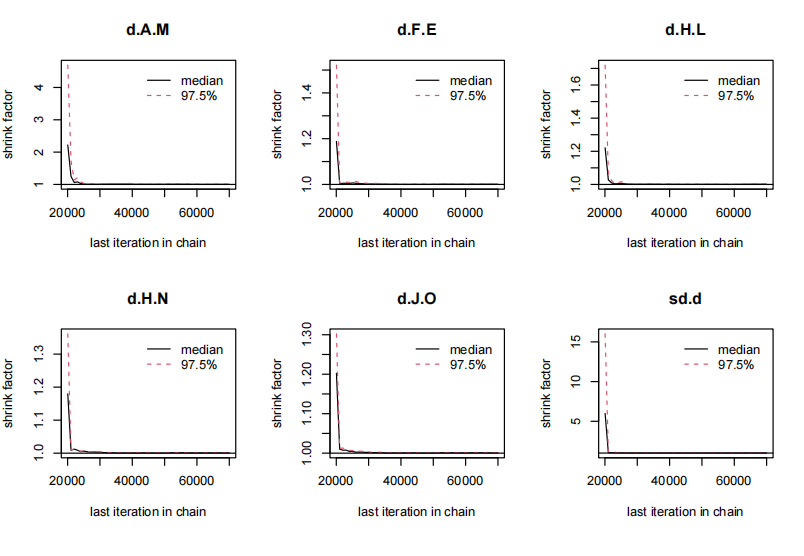


Supplementary Figure 3-b: Diagnostic plot for clinical remission

Abbreviations: A, Placebo; B, AZA; C, CsA; D, HCQ; E, LEF; F, MMF; G, MZR; H, RASI; I, RIT; J, STE; K, STE+MMF; L, STE+RASI; M, Sibeprenlimab; N, Sparsentan; O, TSP.


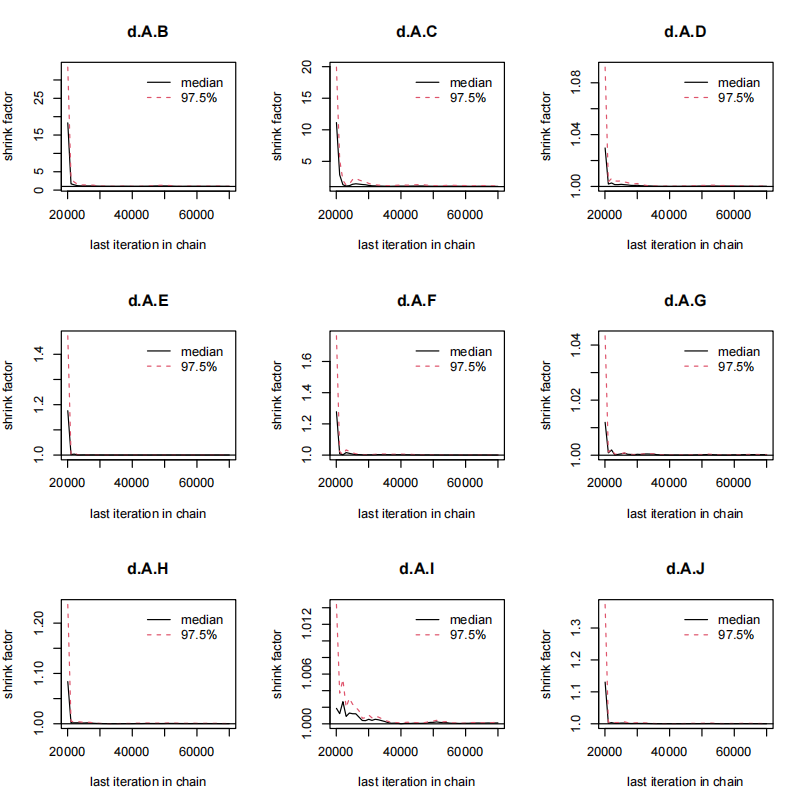


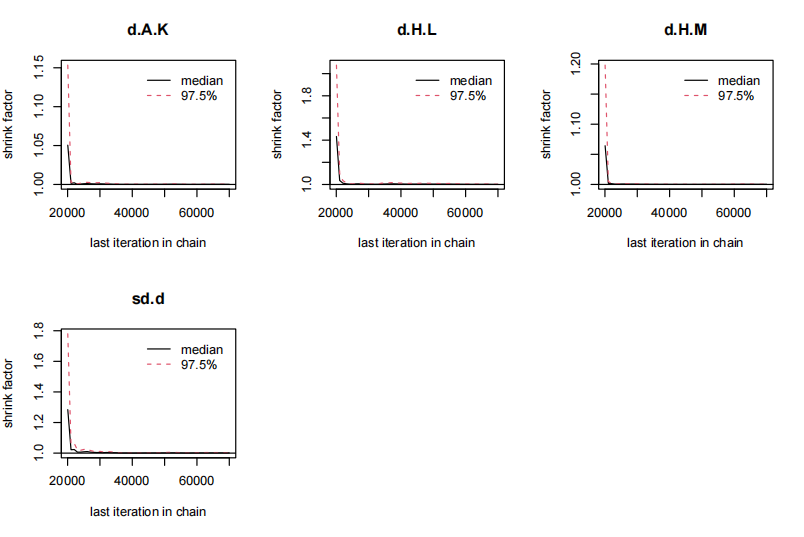


Supplementary Figure 3-c: Diagnostic plot for ESRD or KD

Abbreviations: A, Placebo; B, CsA; C, HCQ; D, LEF; E, MMF; F, MZR; G, Nefecon; H, RASI; I, SGLT2I; J, STE; K, STE+AZA; L, STE+RASI; M, Sparsentan.


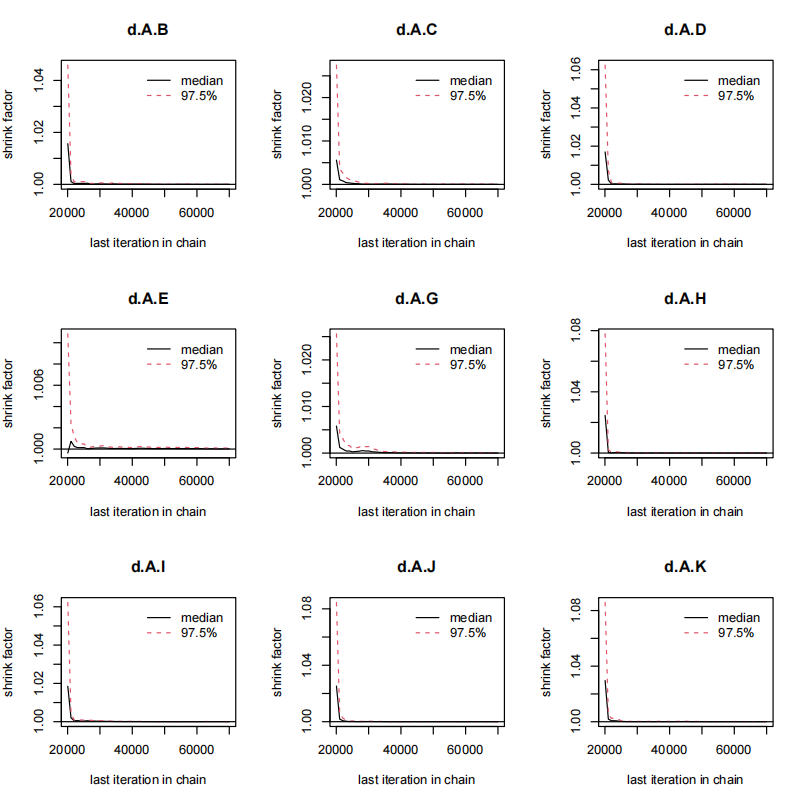


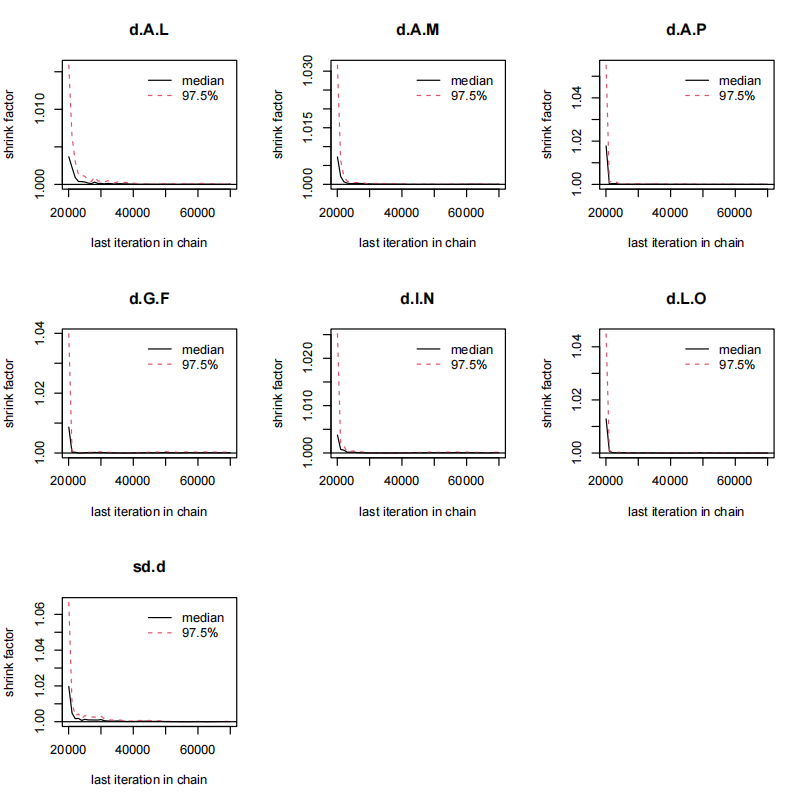


Supplementary Figure 3-d: Diagnostic plot for 24-h UPE

Abbreviations: A, Placebo; B, Atacicept; C, CsA; D, HCQ; E, Iptacopan; F, LEF; G, MMF; H, MZR; I, RASI; J, RIT; K, STE; L, STE+AZA; M, STE+MMF; N, STE+RASI; O, TAC; P, Telitacicept.


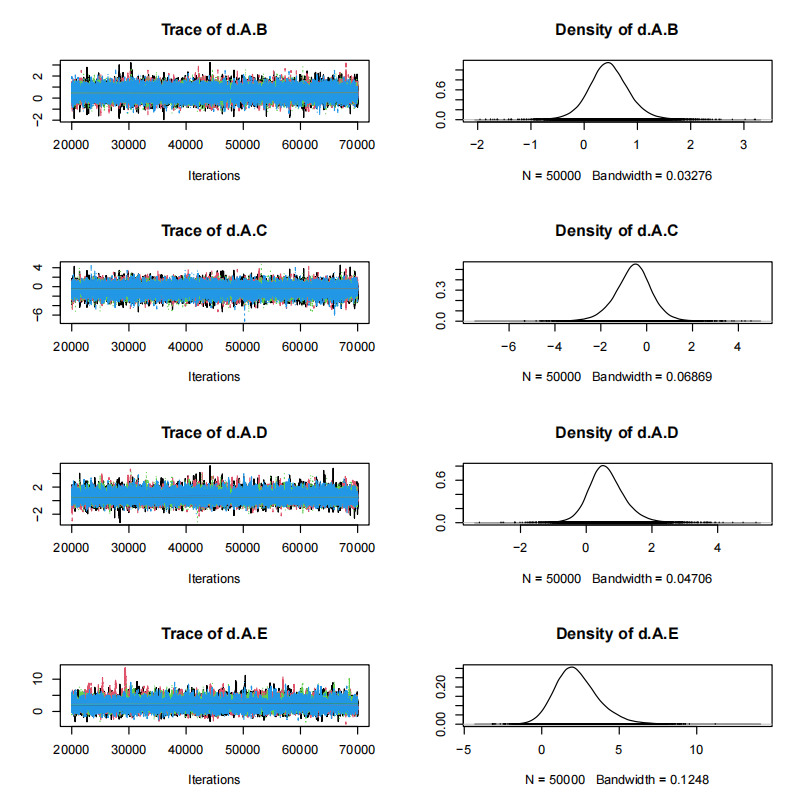


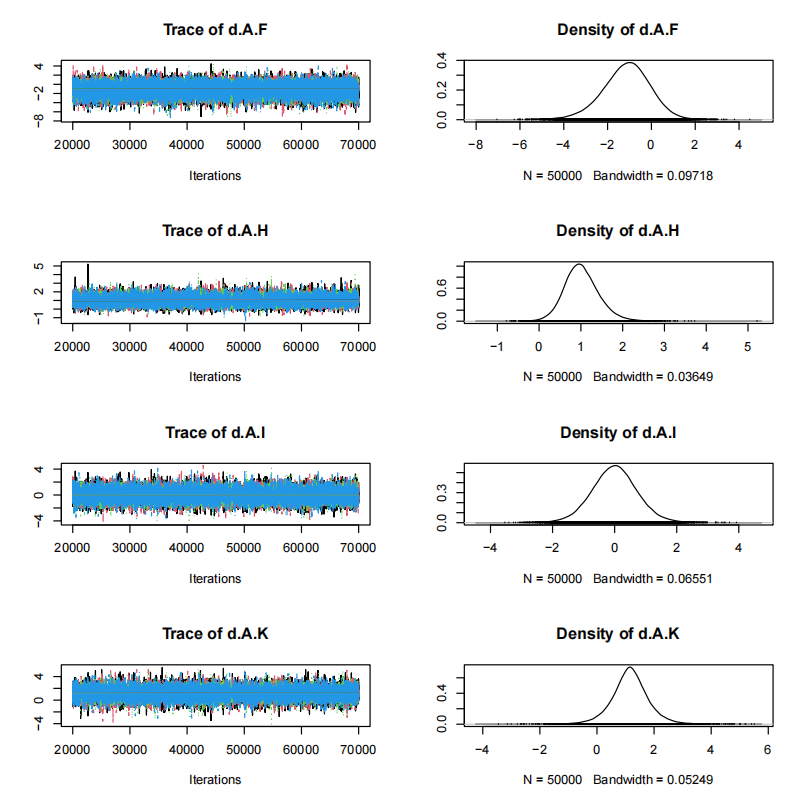


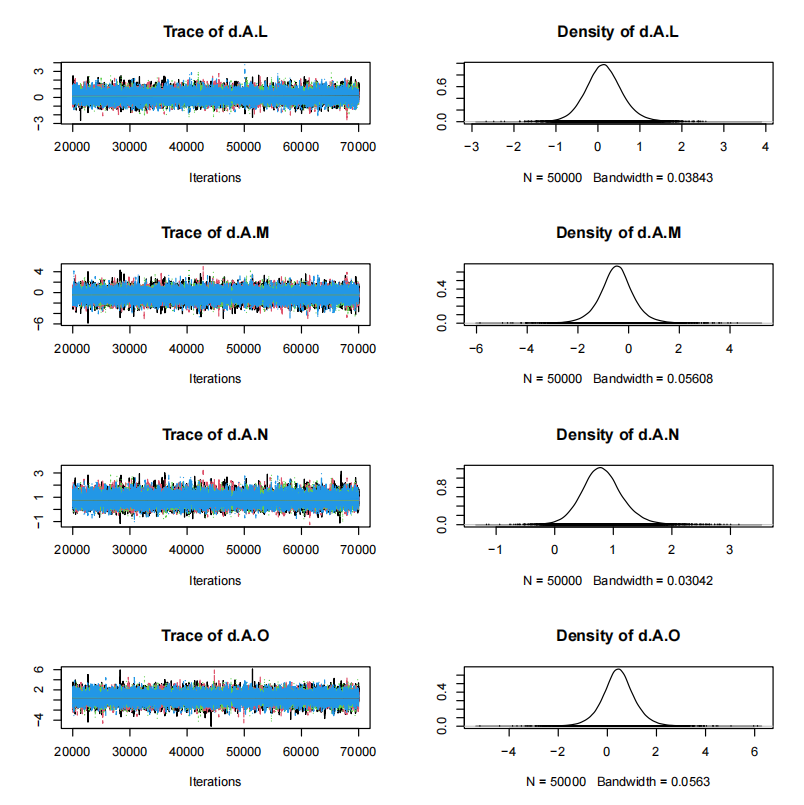


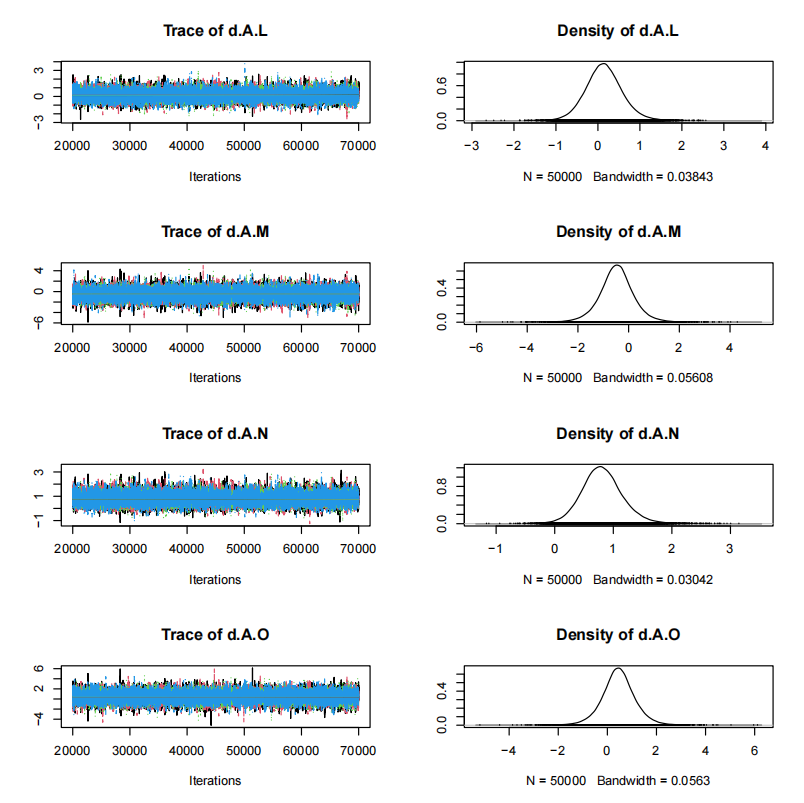


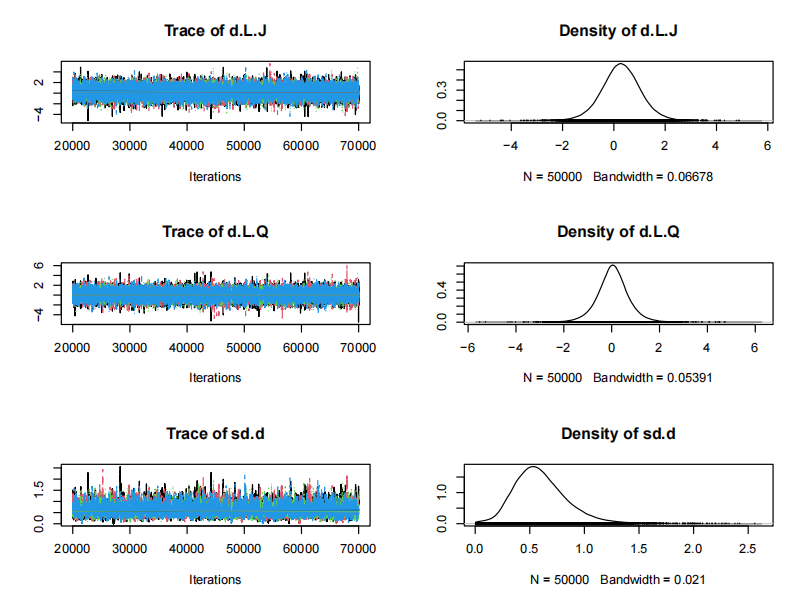


Supplementary Figure 3-A: Trace plots for adverse events

Abbreviations: A, placebo; B, AZA; C, Atacicept; D, CsA; E, HCQ; F, Iptacopan; G, LEF; H, MMF; I, MZR; J, MZR+RASI; K, Nefecon; L, RASI; M, SGLT2I; N, STE; O, STE+MMF; P, Sibeprenlimab; Q, Sparsentan; R, TAC; S, Telitacicept.


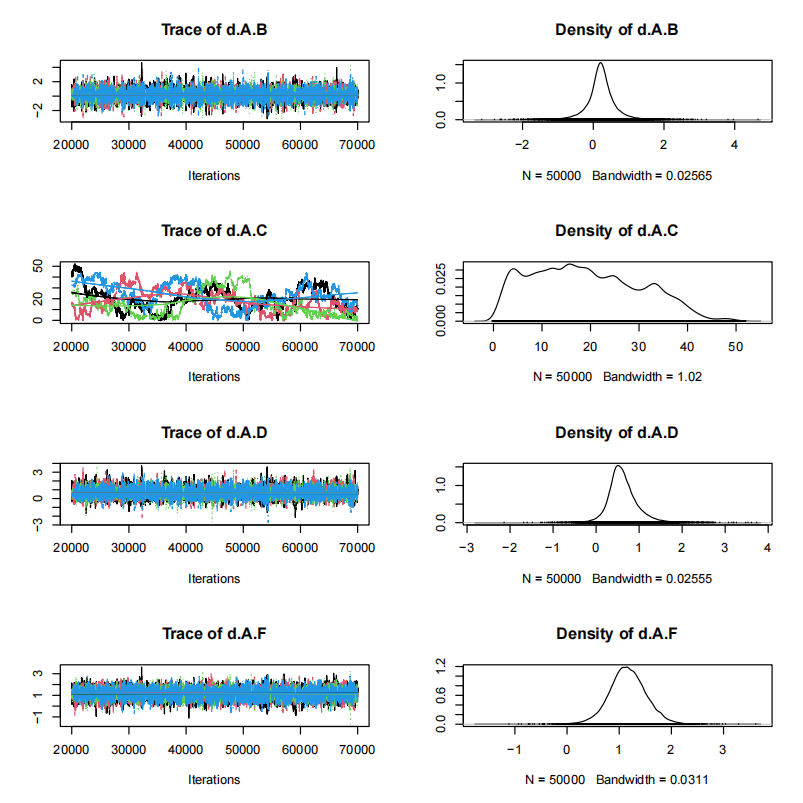


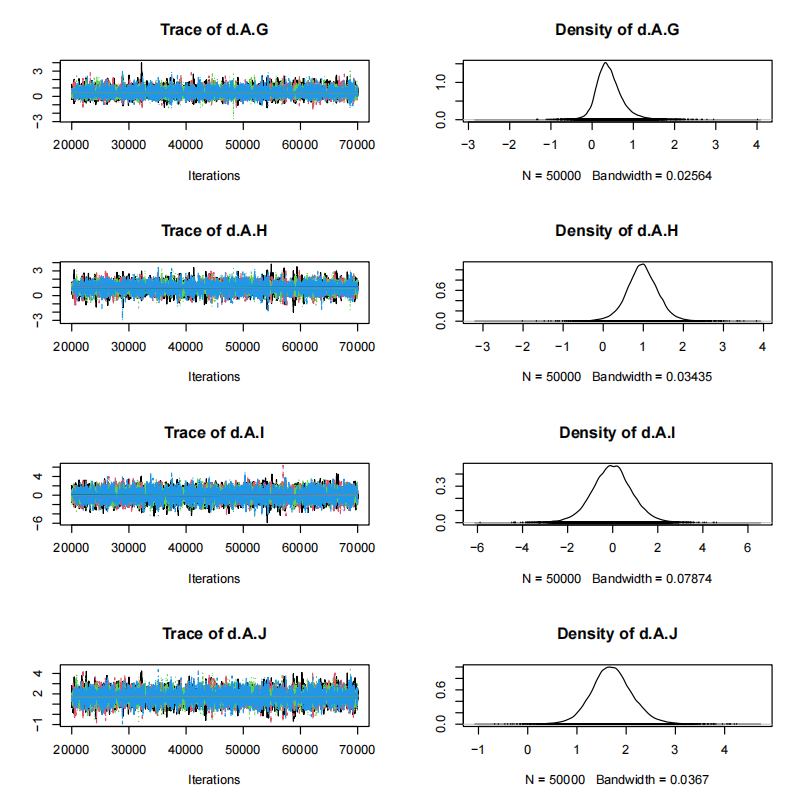


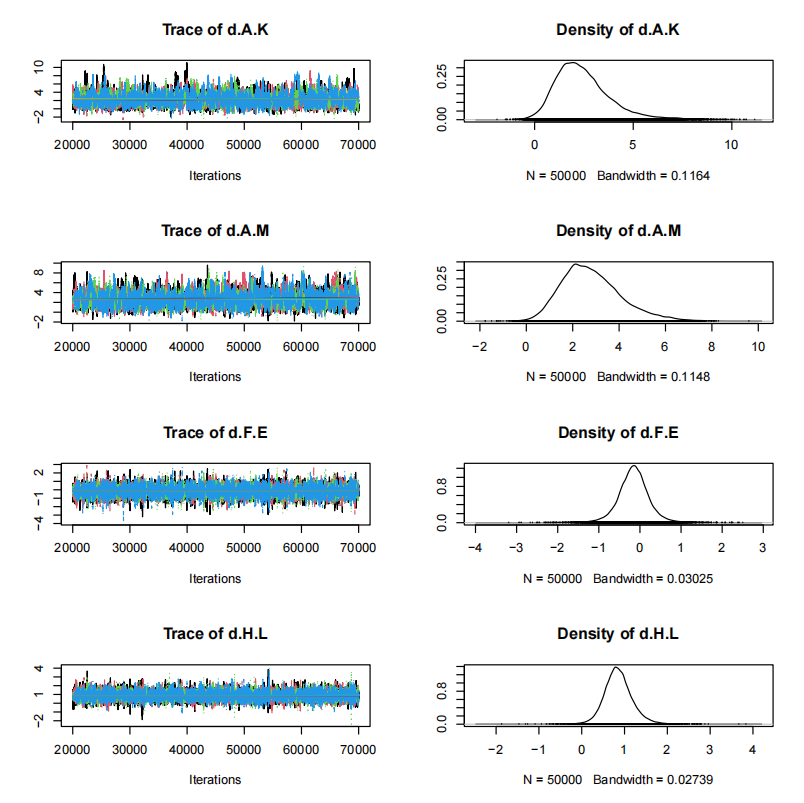


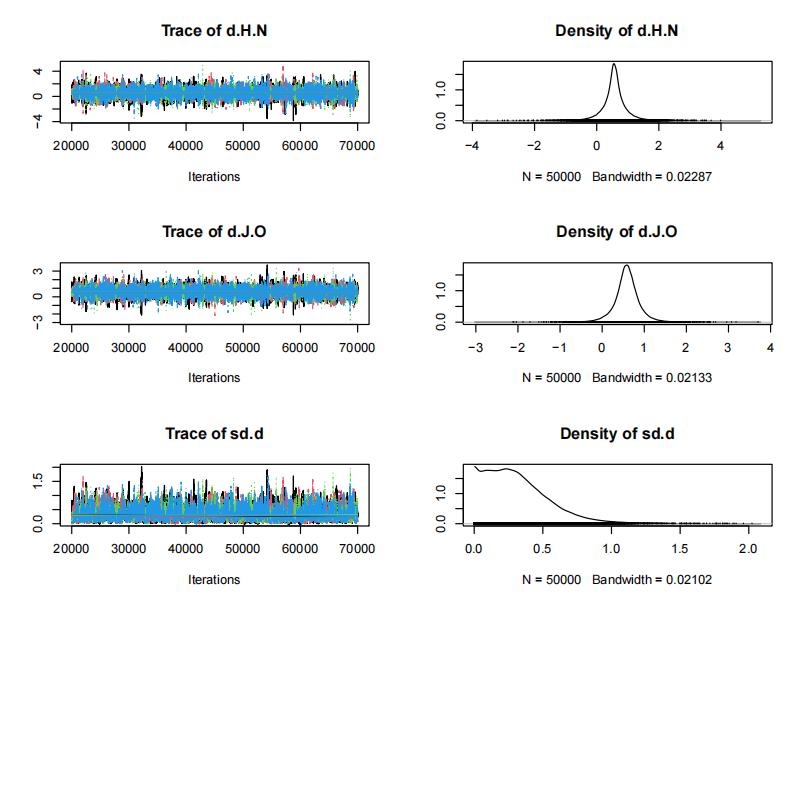


Supplementary Figure 3-B: Trace plots for clinical remission

Abbreviations: Abbreviations: A, Placebo; B, AZA; C, CsA; D, HCQ; E, LEF; F, MMF; G, MZR; H, RASI; I, RIT; J, STE; K, STE+MMF; L, STE+RASI; M, Sibeprenlimab; N, Sparsentan; O, TSP.


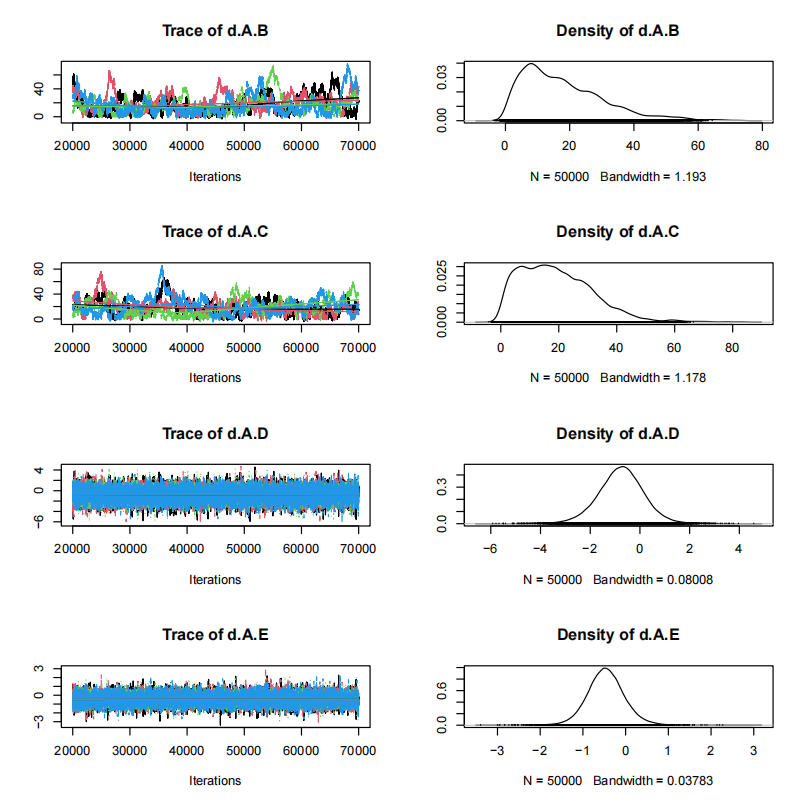


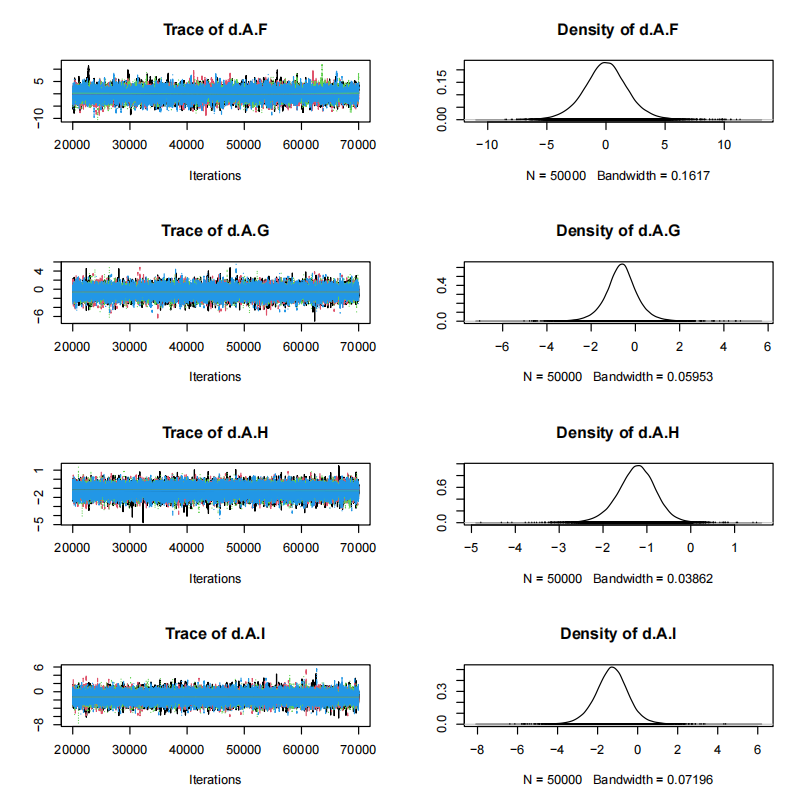


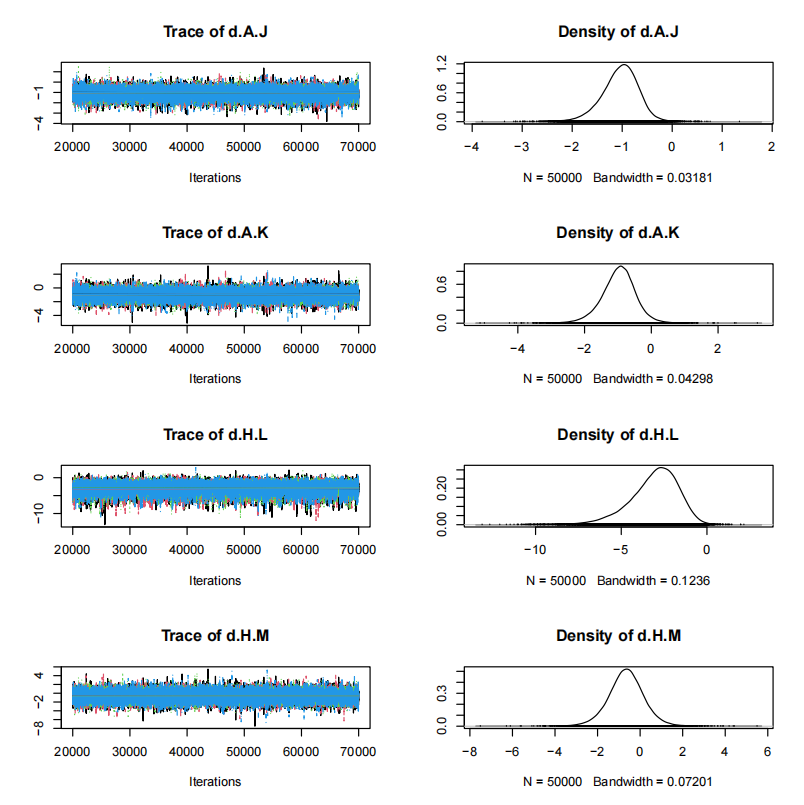


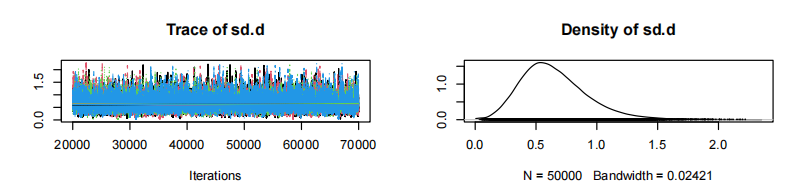


Supplementary Figure 3-C: Trace plots for ESRD or KD

Abbreviations: A, Placebo; B, CsA; C, HCQ; D, LEF; E, MMF; F, MZR; G, Nefecon; H, RASI; I, SGLT2I; J, STE; K, STE+AZA; L, STE+RASI; M, Sparsentan.


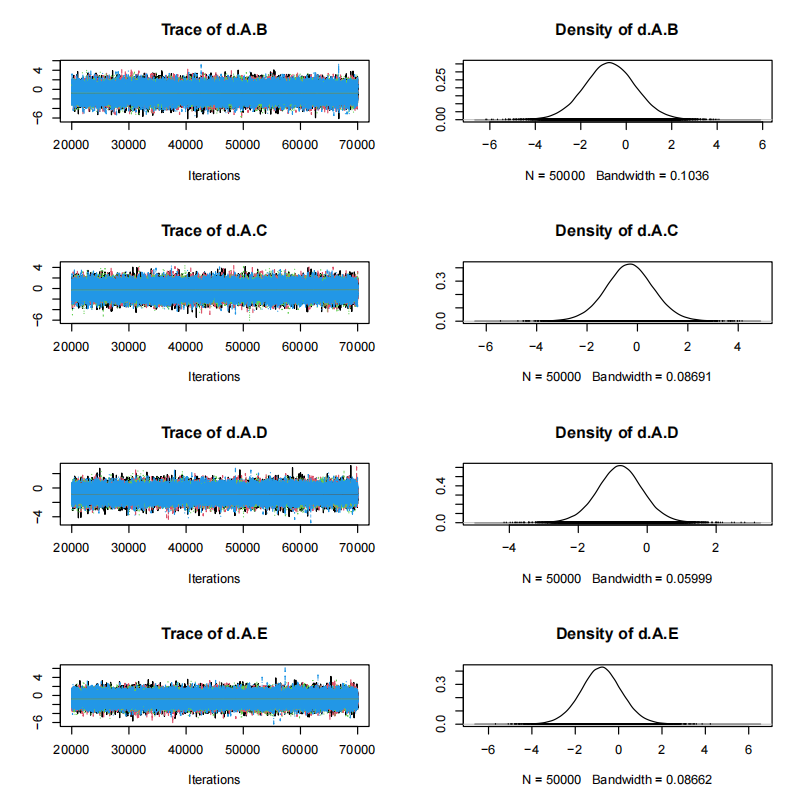


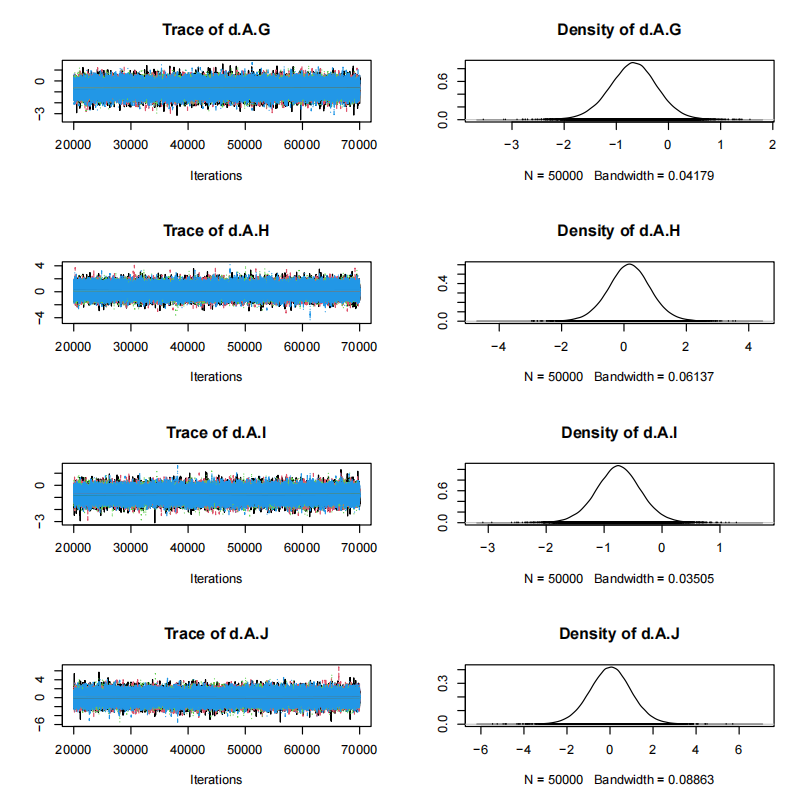


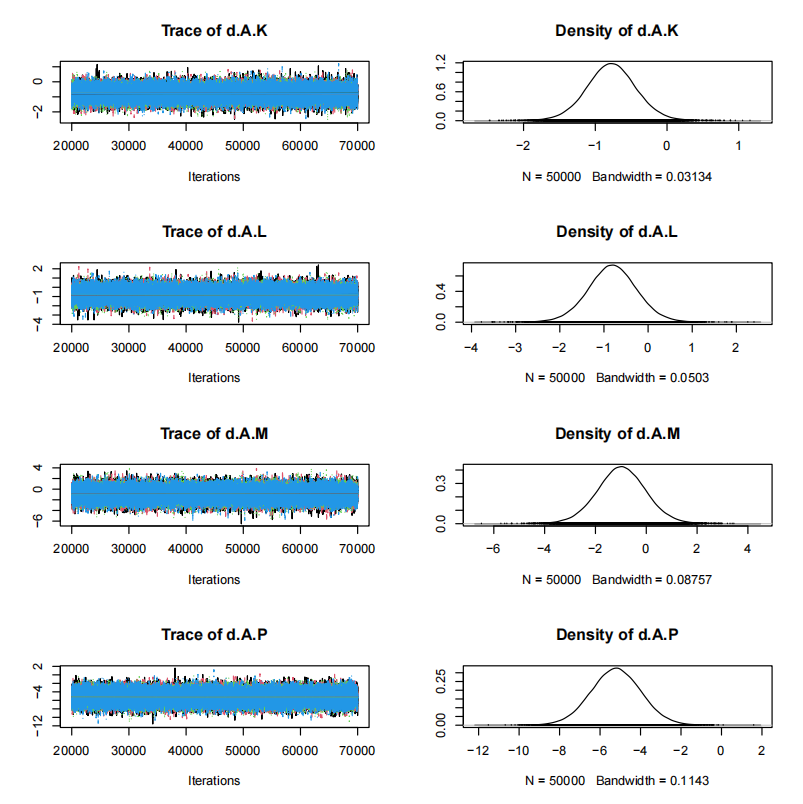


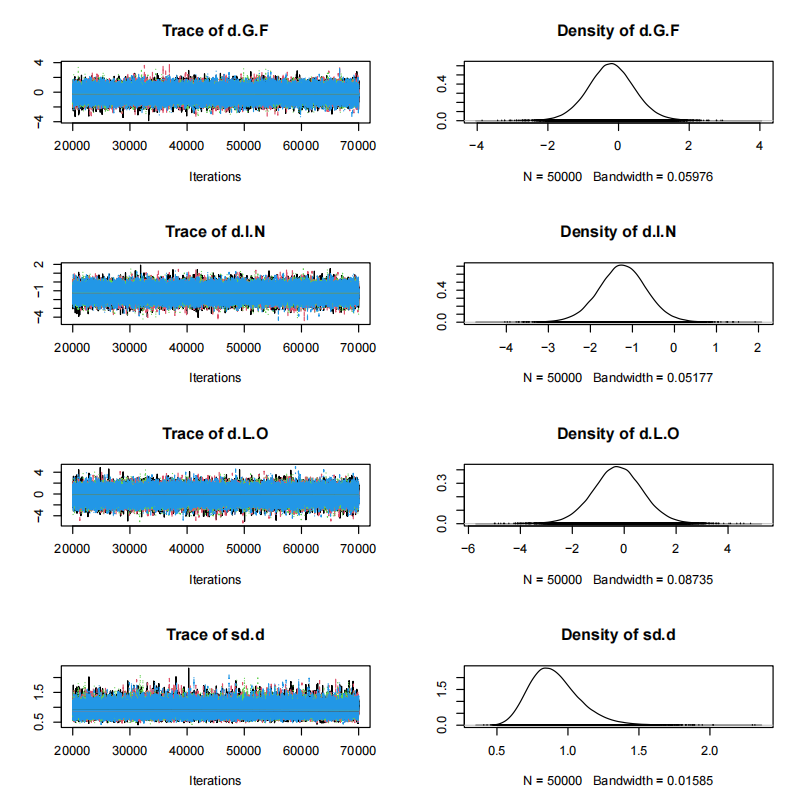


Supplementary Figure 3-D: Trace plots for 24h UPE

Abbreviations: A, Placebo; B, Atacicept; C, CsA; D, HCQ; E, Iptacopan; F, LEF; G, MMF; H, MZR; I, RASI; J, RIT; K, STE; L, STE+AZA; M, STE+MMF; N, STE+RASI; O, TAC; P, Telitacicept.

**Supplementary Figure 4: Heterogeneity analysis on end points**


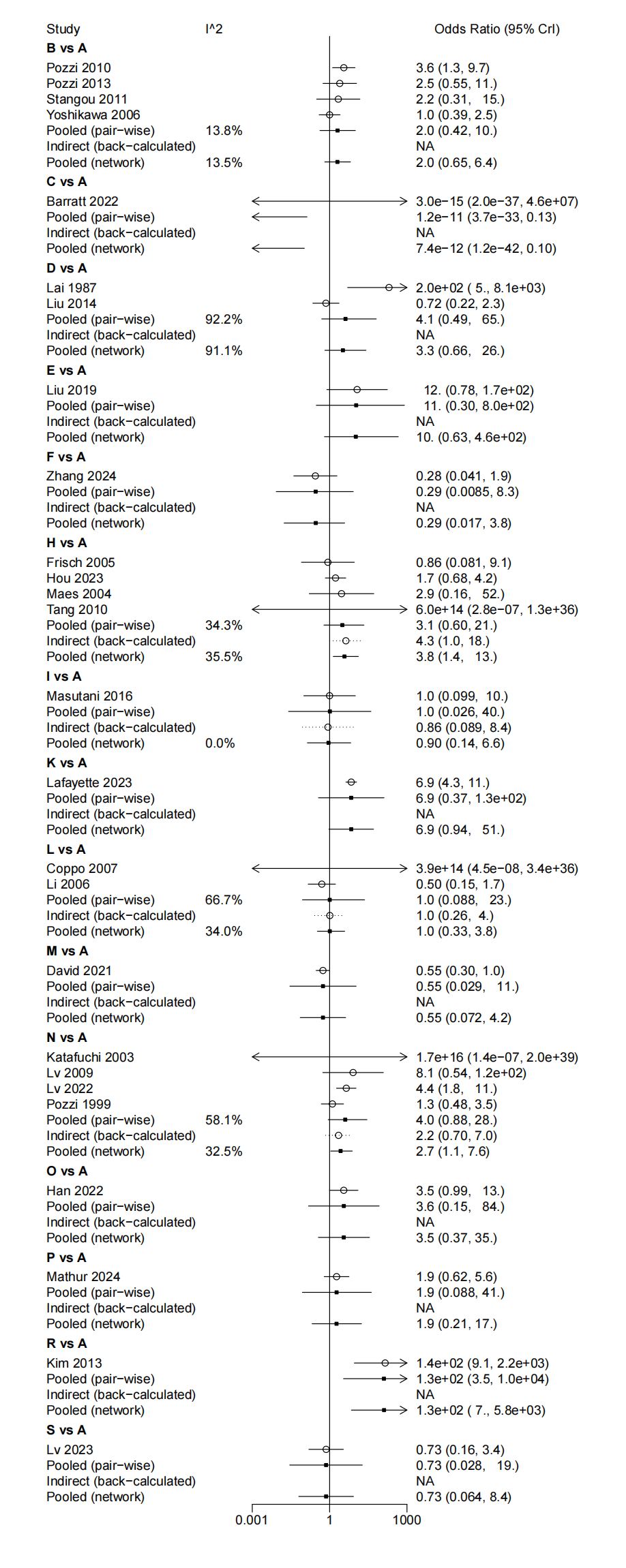


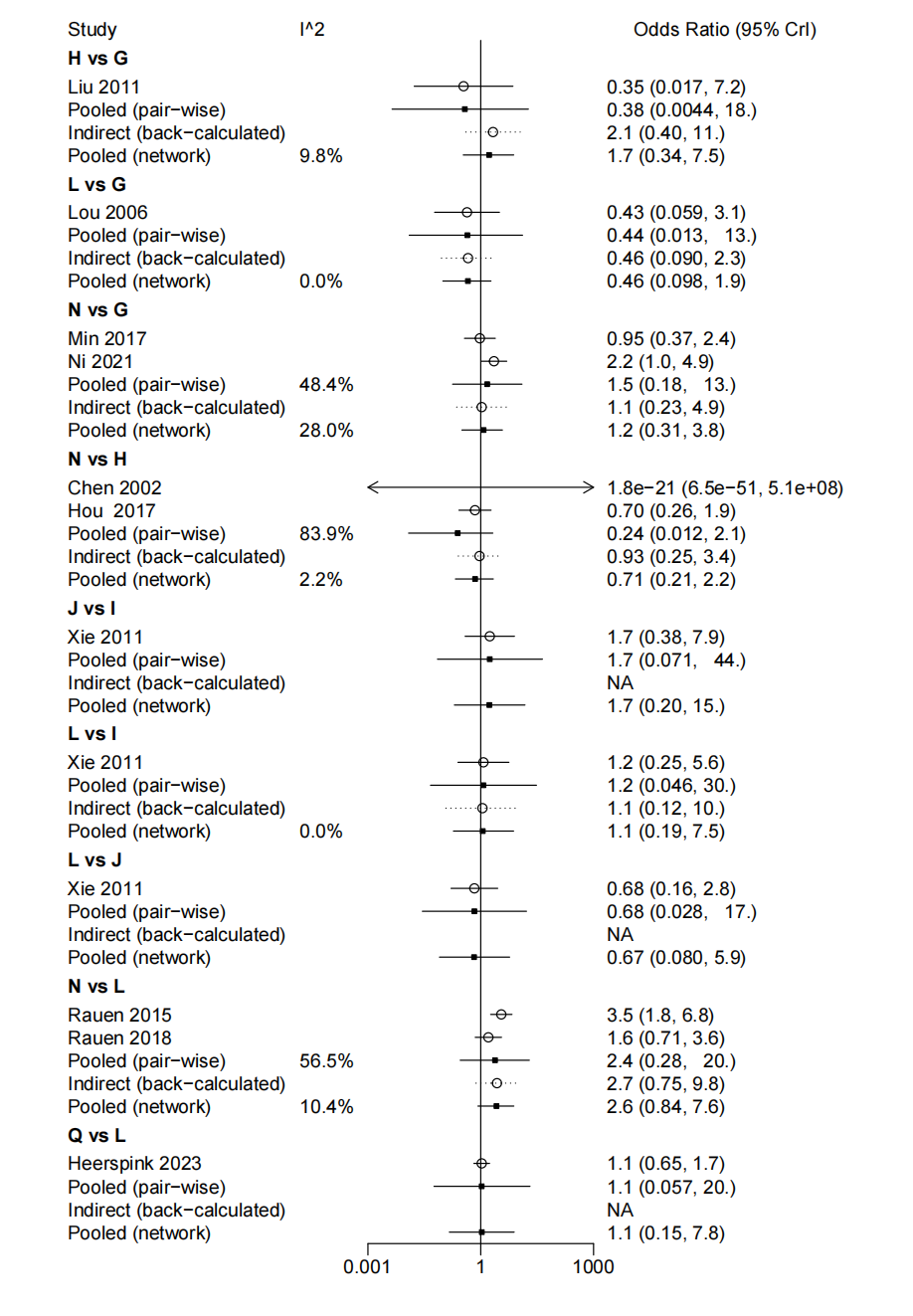


Supplementary Figure 4-A: adverse events

Abbreviations: A, placebo; B, AZA; C, Atacicept; D, CsA; E, HCQ; F, Iptacopan; G, LEF; H, MMF; I, MZR; J, MZR+RASI; K, Nefecon; L, RASI; M, SGLT2I; N, STE; O, STE+MMF; P, Sibeprenlimab; Q, Sparsentan; R, TAC; S, Telitacicept


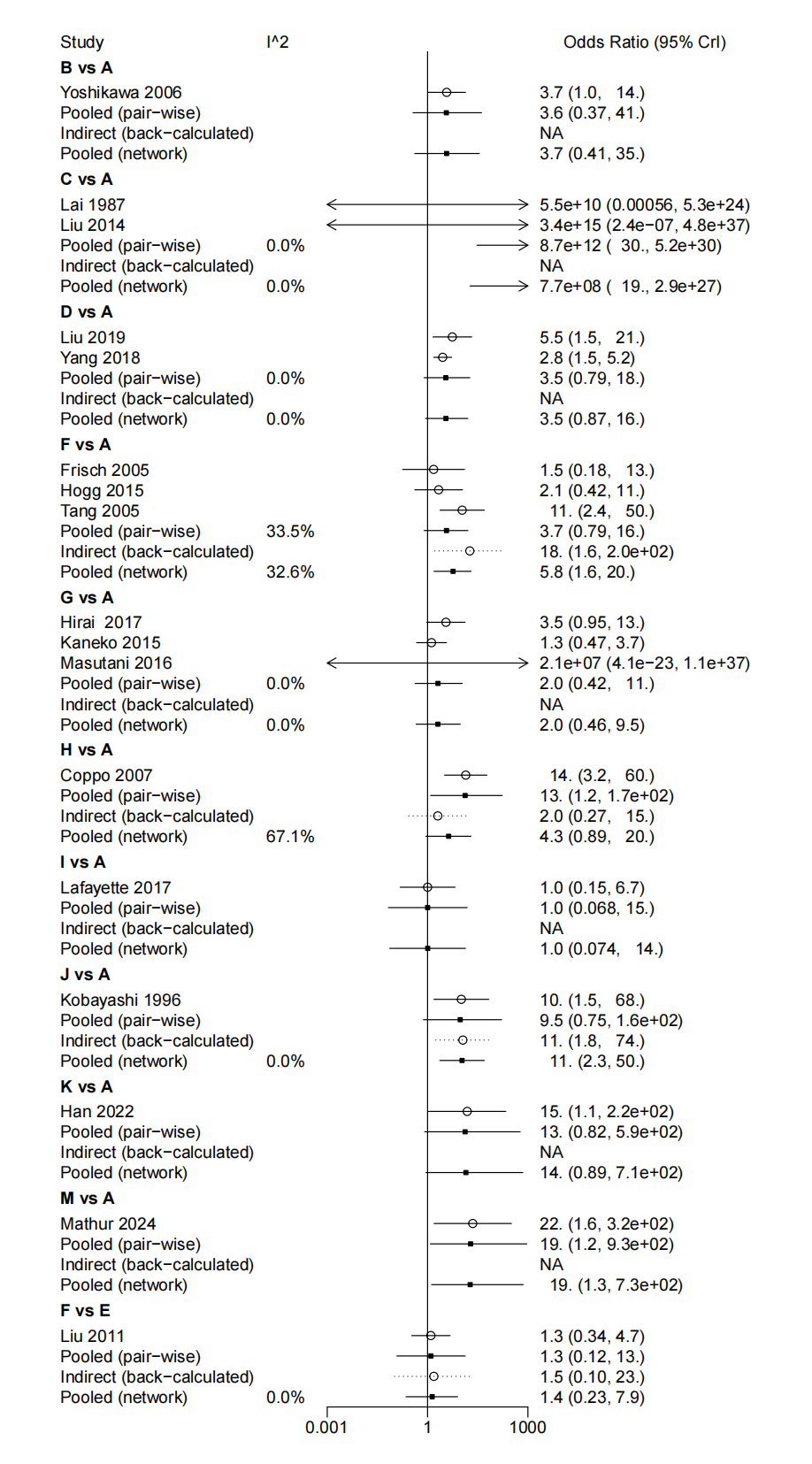


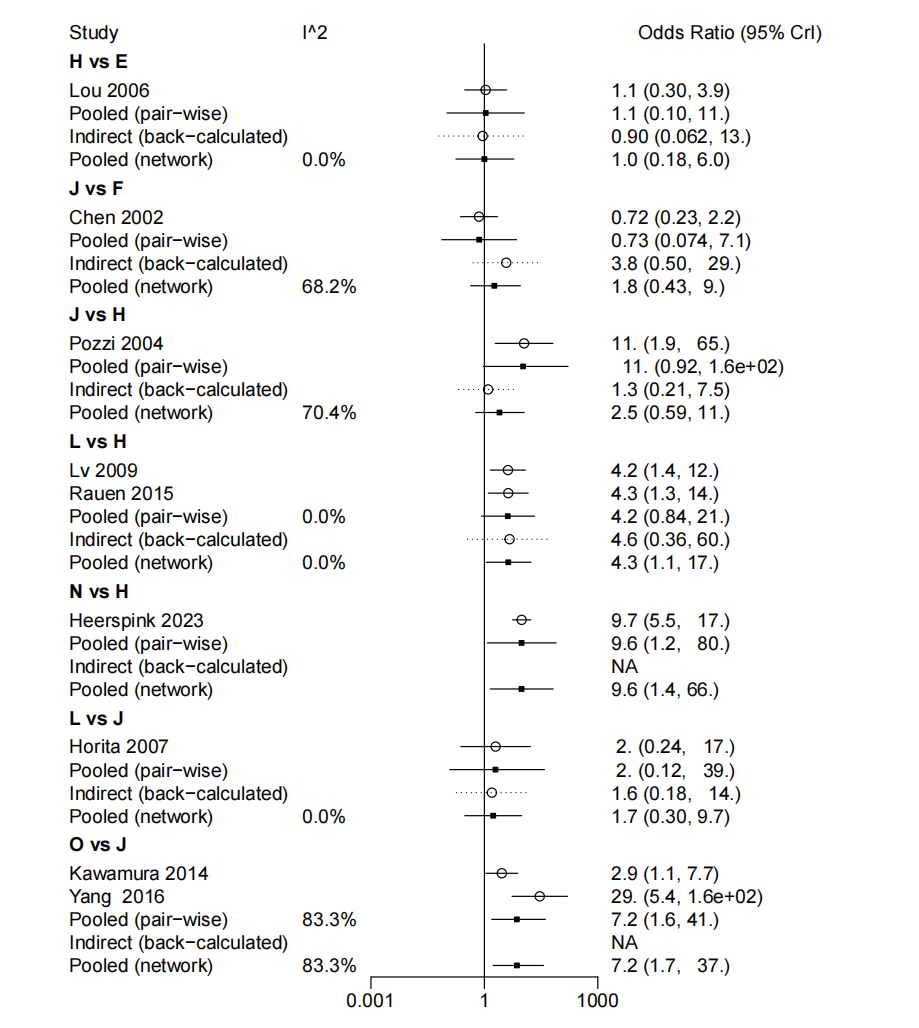


Supplementary Figure 4-B: clinical remission

Abbreviations: Abbreviations: A, Placebo; B, AZA; C, CsA; D, HCQ; E, LEF; F, MMF; G, MZR; H, RASI; I, RIT; J, STE; K, STE+MMF; L, STE+RASI; M, Sibeprenlimab; N, Sparsentan; O, TSP.


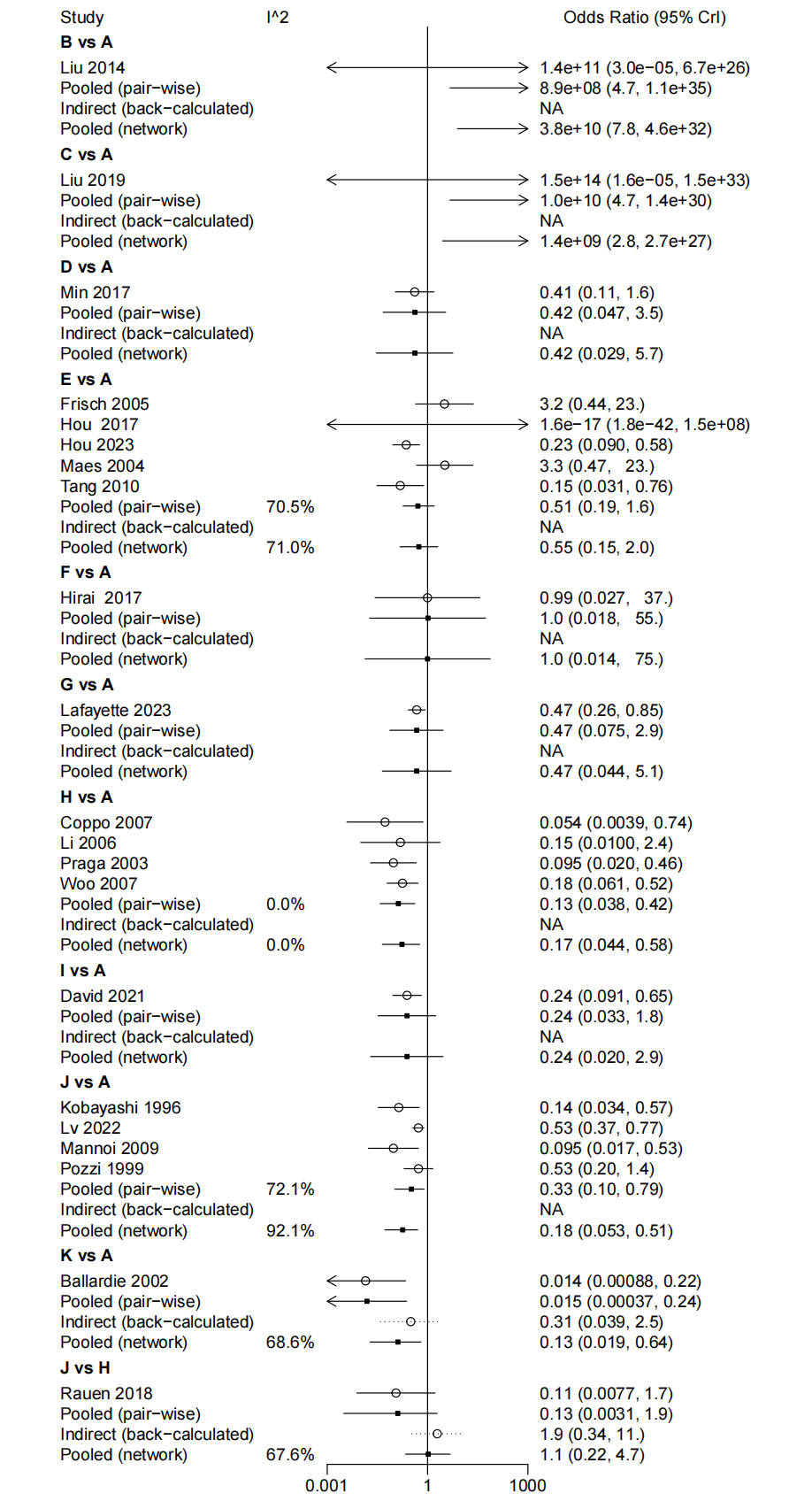


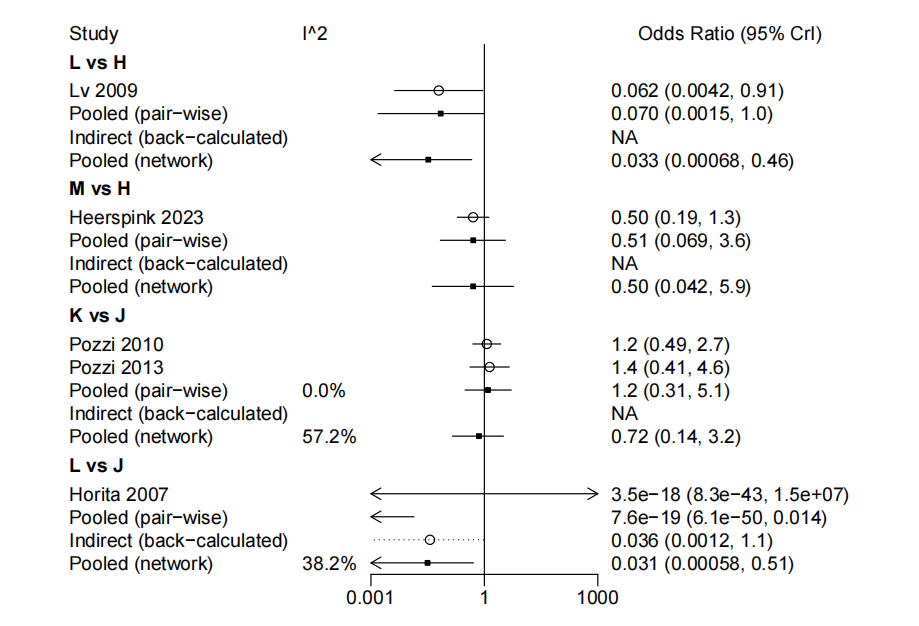


Supplementary Figure 4-C: ESRD or KD

Abbreviations: A, Placebo; B, CsA; C, HCQ; D, LEF; E, MMF; F, MZR; G, Nefecon; H, RASI; I, SGLT2I; J, STE; K, STE+AZA; L, STE+RASI; M, Sparsentan.


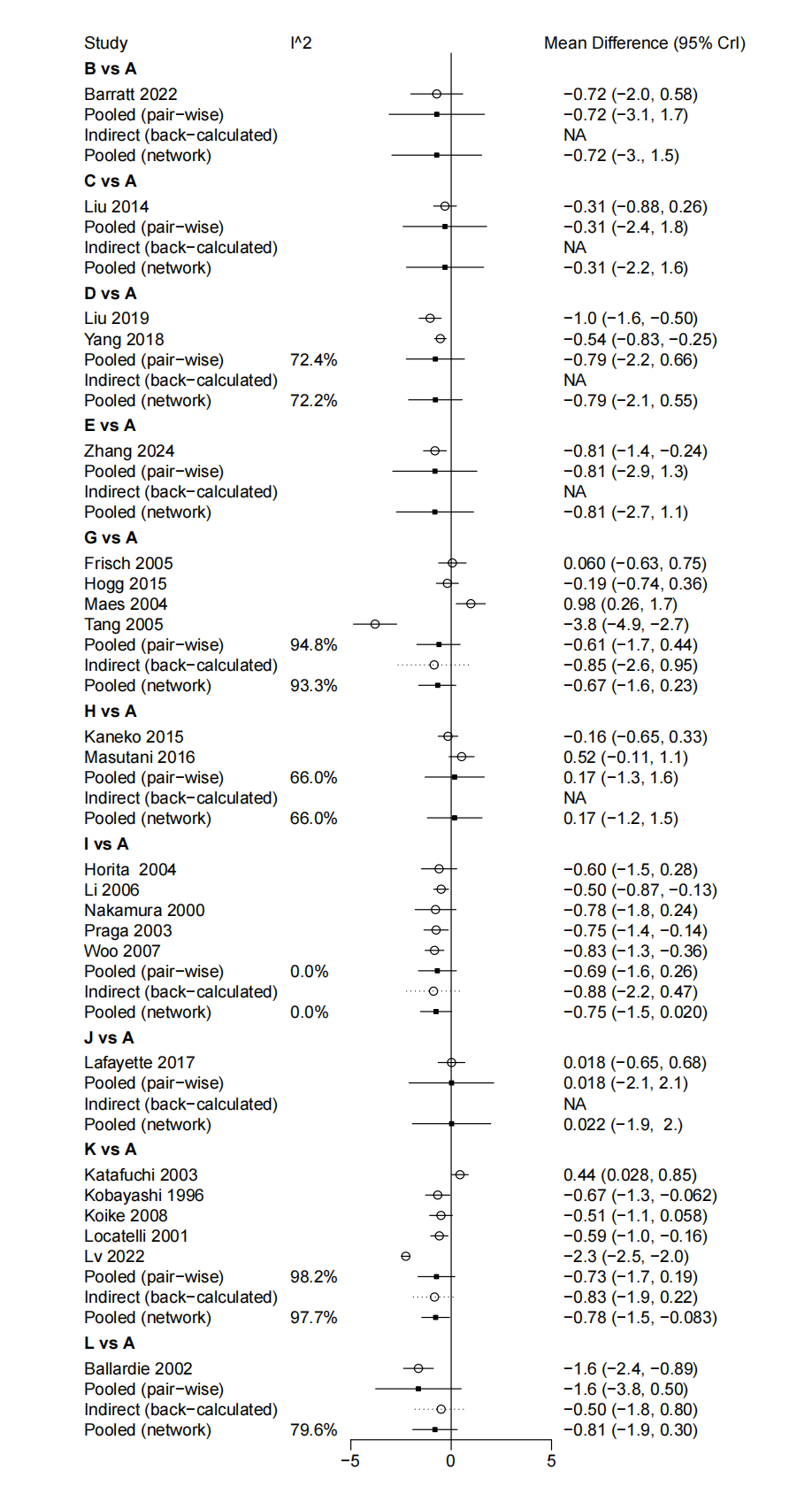


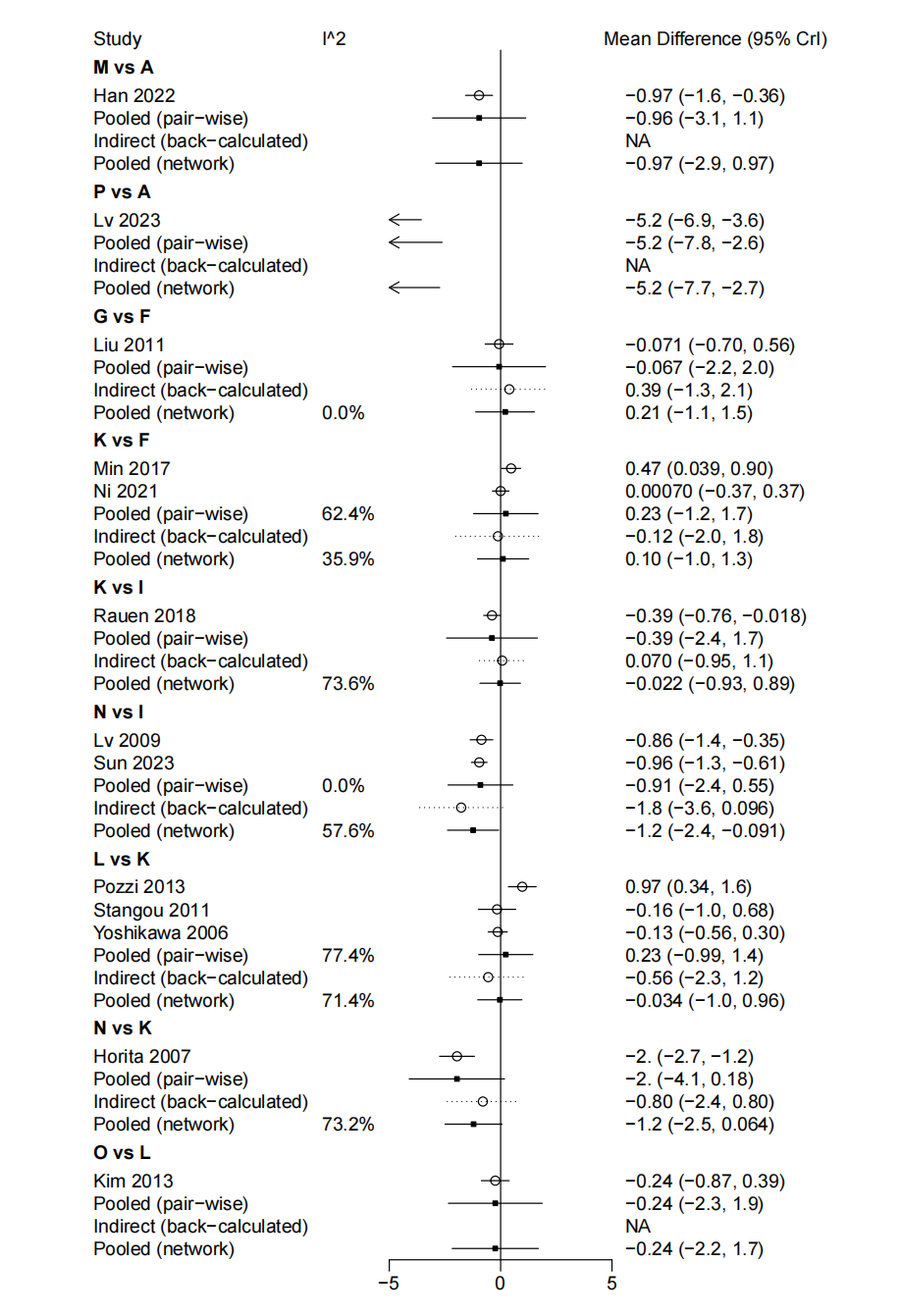


Supplementary Figure 4-D: 24h UPE

Abbreviations: A, Placebo; B, Atacicept; C, CsA; D, HCQ; E, Iptacopan; F, LEF; G, MMF; H, MZR; I, RASI; J, RIT; K, STE; L, STE+AZA; M, STE+MMF; N, STE+RASI; O, TAC; P, Telitacicept.

**Supplementary Figure 5: Inconsistency using node-splitting approach**


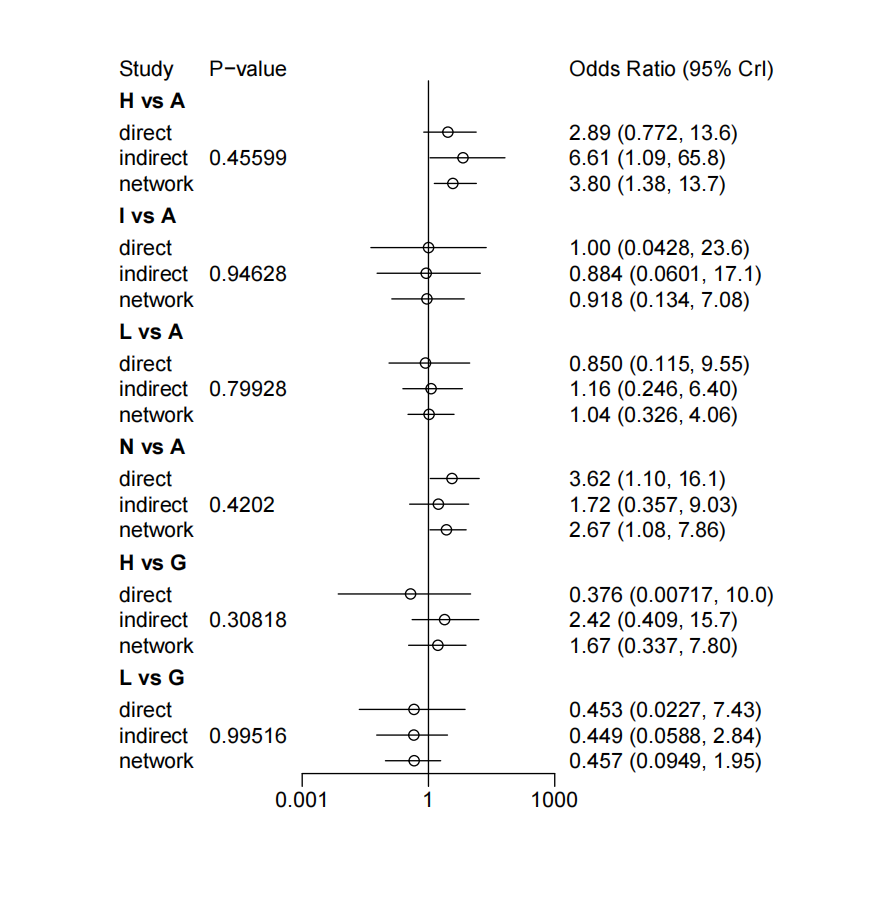


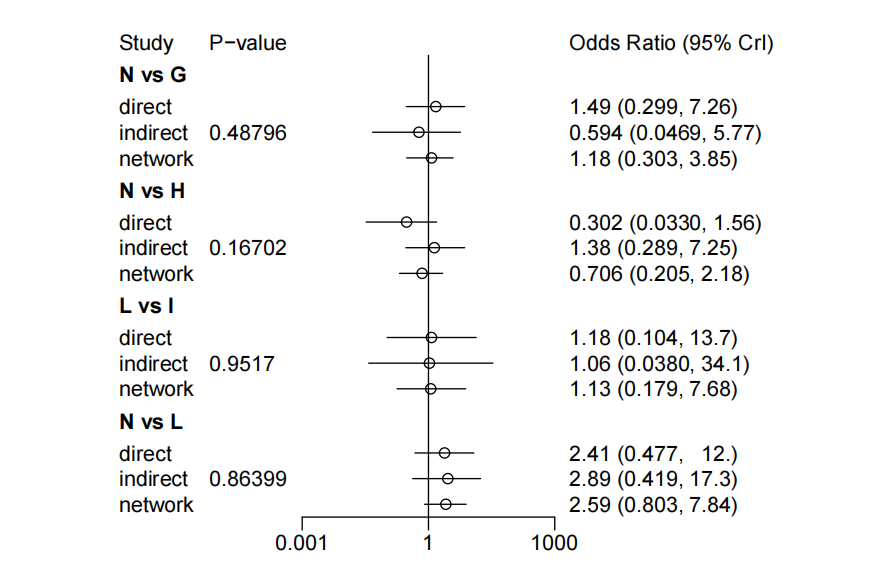


Supplementary Figure 5-A: adverse events

Abbreviations: A, placebo; B, AZA; C, Atacicept; D, CsA; E, HCQ; F, Iptacopan; G, LEF; H, MMF; I, MZR; J, MZR+RASI; K, Nefecon; L, RASI; M, SGLT2I; N, STE; O, STE+MMF; P, Sibeprenlimab; Q, Sparsentan; R, TAC; S, Telitacicept


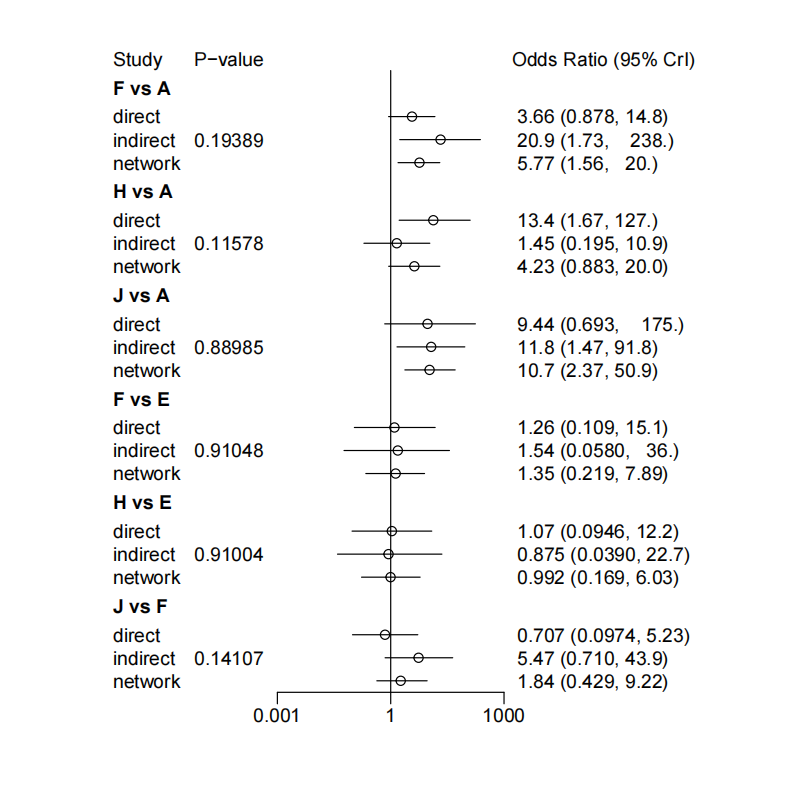


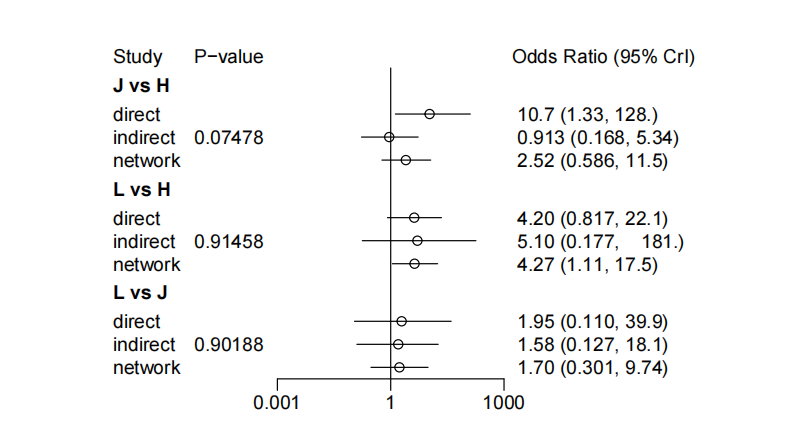


Supplementary Figure 5-B: clinical remission

Abbreviations: Abbreviations: A, Placebo; B, AZA; C, CsA; D, HCQ; E, LEF; F, MMF; G, MZR; H, RASI; I, RIT; J, STE; K, STE+MMF; L, STE+RASI; M, Sibeprenlimab; N, Sparsentan; O, TSP.


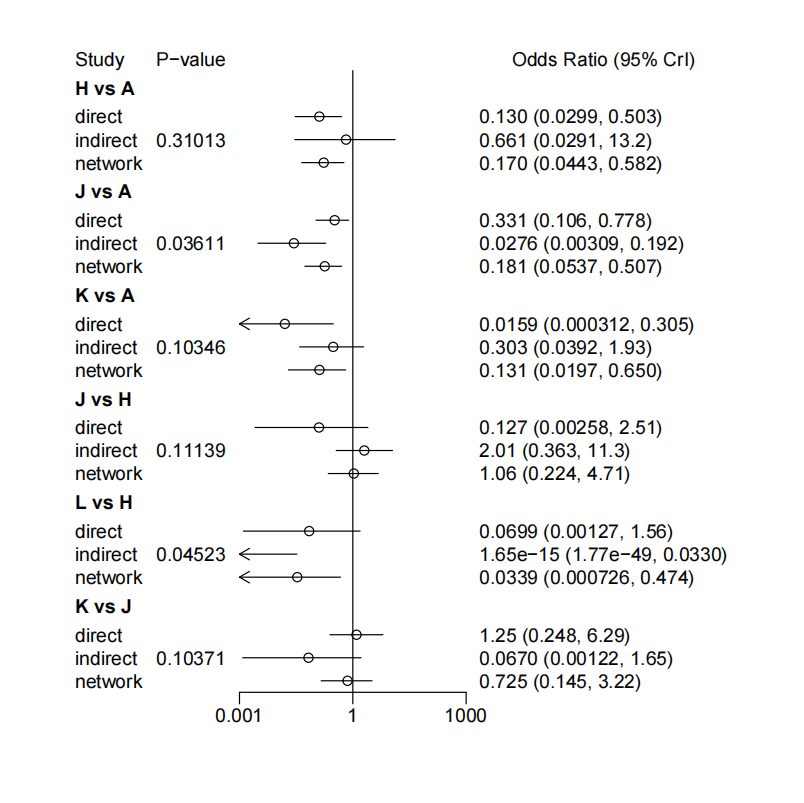


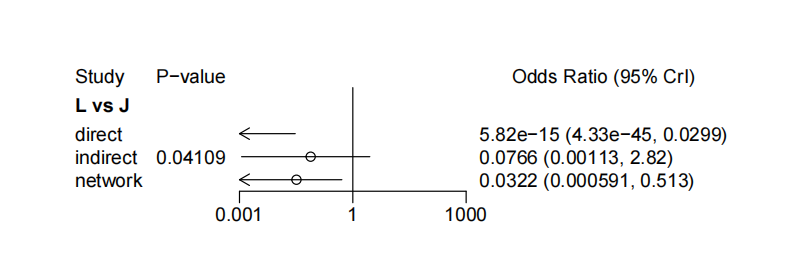


Supplementary Figure 5-C: ESRD or KD

Abbreviations: A, Placebo; B, CsA; C, HCQ; D, LEF; E, MMF; F, MZR; G, Nefecon; H, RASI; I, SGLT2I; J, STE; K, STE+AZA; L, STE+RASI; M, Sparsentan.


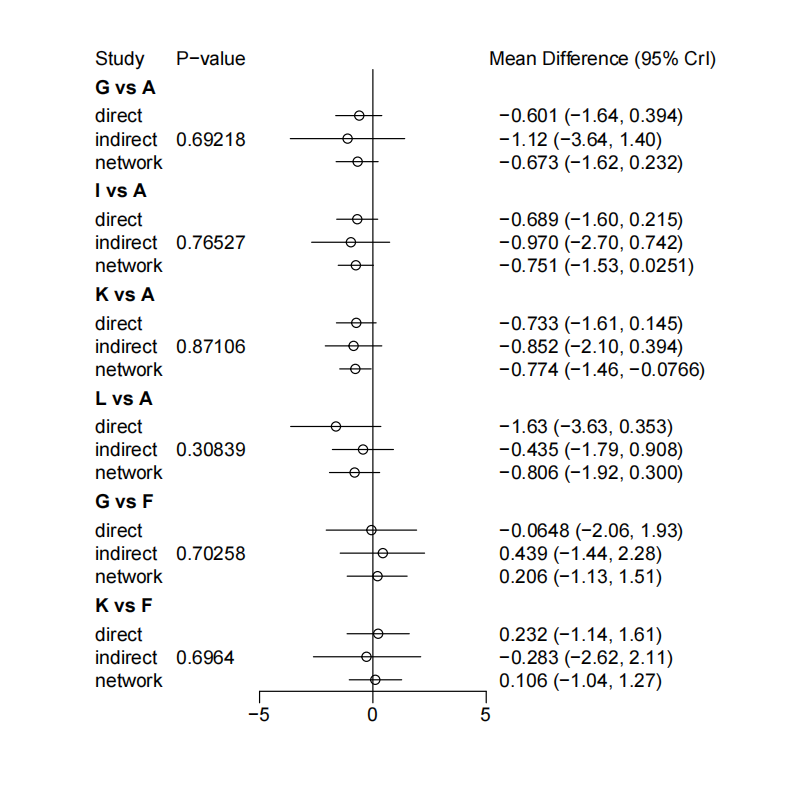


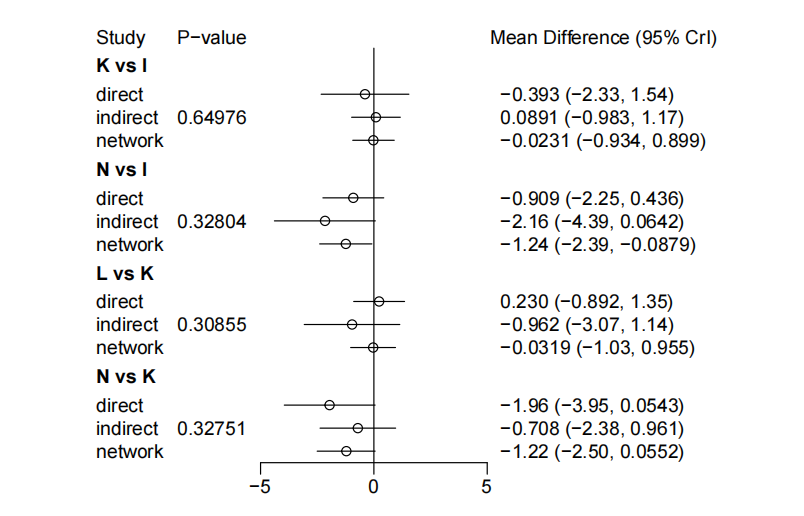


Supplementary Figure 5-D: 24h UPE

Abbreviations: A, Placebo; B, Atacicept; C, CsA; D, HCQ; E, Iptacopan; F, LEF; G, MMF; H, MZR; I, RASI; J, RIT; K, STE; L, STE+AZA; M, STE+MMF; N, STE+RASI; O, TAC; P, Telitacicept.

**Supplementary Figure 6: Publication bias of funnel plot**


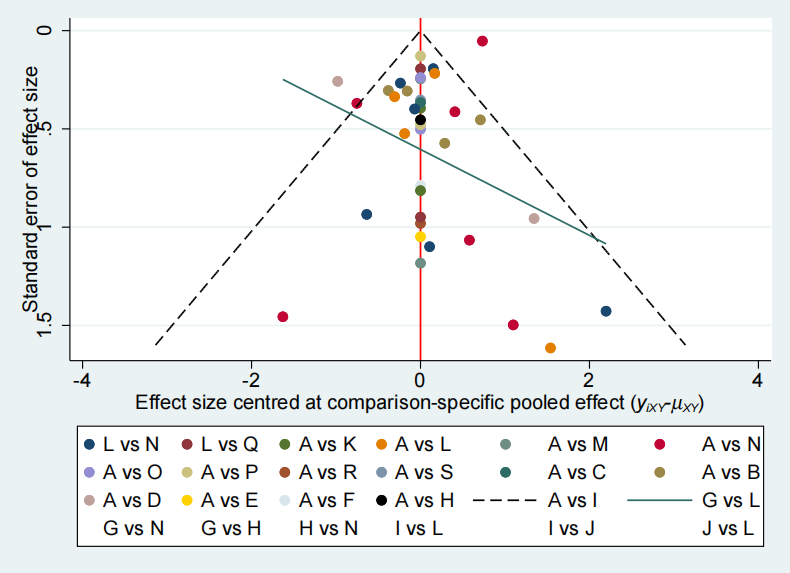


Supplementary Figure 6-A: adverse events

Abbreviations: A, placebo; B, AZA; C, Atacicept; D, CsA; E, HCQ; F, Iptacopan; G, LEF; H, MMF; I, MZR; J, MZR+RASI; K, Nefecon; L, RASI; M, SGLT2I; N, STE; O, STE+MMF; P, Sibeprenlimab; Q, Sparsentan; R, TAC; S, Telitacicept.


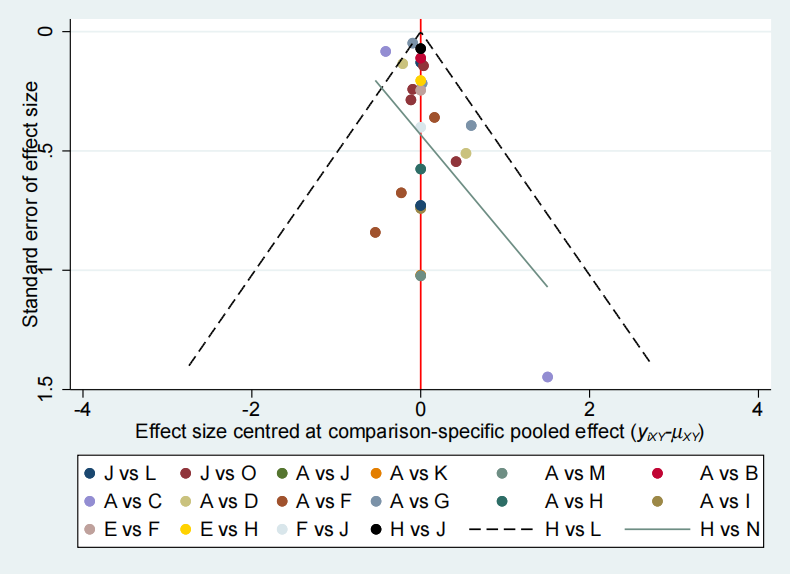


Supplementary Figure 6-B: clinical remission

Abbreviations: Abbreviations: A, Placebo; B, AZA; C, CsA; D, HCQ; E, LEF; F, MMF; G, MZR; H, RASI; I, RIT; J, STE; K, STE+MMF; L, STE+RASI; M, Sibeprenlimab; N, Sparsentan; O, TSP.


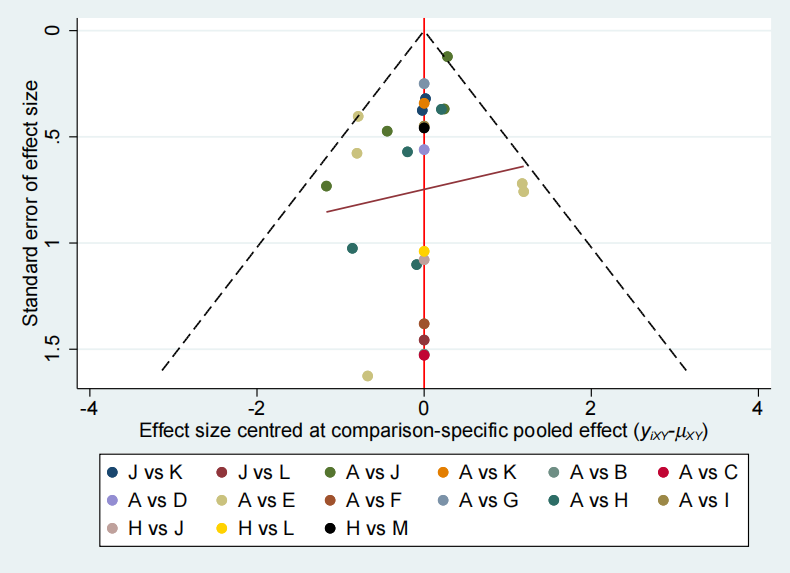


Supplementary Figure 6-C: ESRD or KD

Abbreviations: A, Placebo; B, CsA; C, HCQ; D, LEF; E, MMF; F, MZR; G, Nefecon; H, RASI;I, SGLT2I; J, STE; K, STE+AZA; L, STE+RASI; M, Sparsentan.


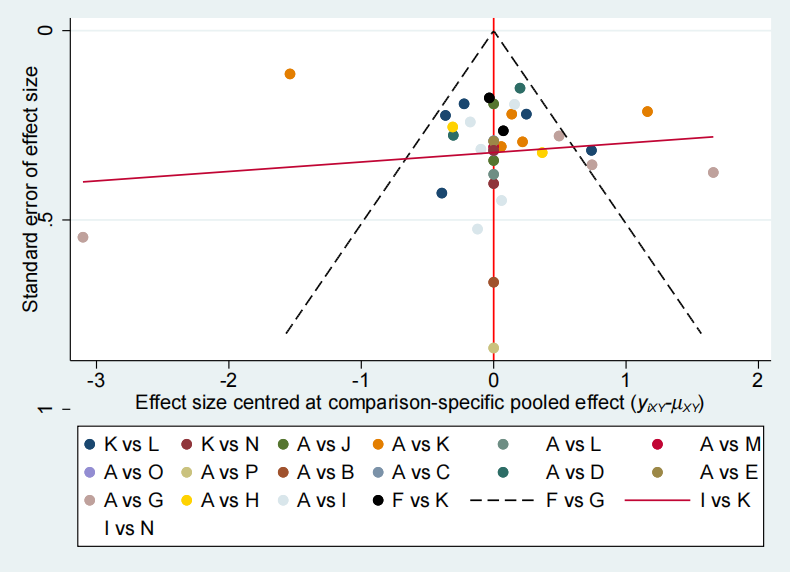


Supplementary Figure 6-D: 24-h UPE

Abbreviations: A, Placebo; B, Atacicept; C, CsA; D, HCQ; E, Iptacopan; F, LEF; G, MMF; H, MZR; I, RASI; J, RIT; K, STE; L, STE+AZA; M, STE+MMF; N, STE+RASI; O, TAC; P, Telitacicept.

**Supplementary Figure 7: Result of sensitivity analysis**

Supplementary Figure 7-A: adverse events

Supplementary Figure 7-B: clinical remission

Supplementary Figure 7-C: ESRD or KD

Supplementary Figure 7-D: 24-h UPE

**S6: Table 6 Results from pairwise meta-analyses**

S6-A adverse events

| **Contrast** | **No. of study** | **RR** | **95%CI** | **Q** | ***I*^2^** | **tau^2^** | ***P*** |
| --- | --- | --- | --- | --- | --- | --- | --- |
| Placebo vs STE | 4 | 0.40 | [0.18, 0.89] | 5.33 | 43.8% | 0.26 | 0.149 |
| STE vs RASI | 2 | 4.06 | [2.01, 8.17] | 0.56 | 0.0% | 0.00 | 0.000 |
| RASI vs LEF | 1 | 0.98 | [0.65, 1.46] | - | 0.0% | - | 0.000 |
| Placebo vs RASI | 2 | 1.42 | [0.55, 3.71] | 1.01 | 1.4 | 0.01 | 0.310 |
| MZR+RASI vs RASI | 1 | 1.33 | [0.55, 3.25] | - | 0.0% | - | 0.000 |
| MMF vs LEF | 1 | 0.92 | [0.57, 1.49] | 0 | 0.0% | - | 0.000 |
| Placebo vs AZA | 4 | 0.68 | [0.43, 1.08] | 4.44 | 32.5% | 0.70 | 0.218 |
| Placebo vs TAC | 1 | 0.06 | [0.01, 0.43] | - | 0.0% | - | 0.000 |
| Placebo vs Atacicept | 1 | 1.50 | [0.74, 3.05] | - | 0.0% | - | 0.000 |
| Placebo vs Telitacicept | 1 | 1.14 | [0.57, 2.29] | - | 0.0% | - | 0.000 |
| Placebo vs MZR | 1 | 2.00 | [0.93, 4.32] | - | 0.0% | - | 0.000 |
| Placebo vs SGLT2I | 1 | 1.59 | [0.98, 2.57] | - | 0.0% | - | 0.000 |
| Placebo vs Iptacopan | 1 | 2.60 | [0.55, 12.19] | - | 0.0% | - | 0.000 |
| Placebo vs HCQ | 1 | 3.25 | [1.20, 8.83] | - | 0.0% | - | 0.000 |
| LEF vs STE | 2 | 0.76 | [0.49, 1.19] | 1.19 | 28.9% | 0.32 | 0.236 |
| Placebo vs STE+MMF | 1 | 0.65 | [0.41, 1.04] | - | 0.0% | - | 0.000 |
| RASI vs Sparsentan | 1 | 0.95 | [0.65, 1.40] | - | 0.0% | - | 0.000 |
| CsA vs Placebo | 2 | 0.43 | [0.05, 4.09] | 5.51 | 81.9% | 2.21 | 0.019 |
| Placebo vs Sibeprenlimab | 1 | 0.87 | [0.68, 1.12] | - | 0.0% | - | 0.000 |
| Placebo vs MMF | 4 | 0.57 | [0.30, 1.09] | 2.86 | 0.00% | 0.00 | 0.414 |
| RASI vs MZR | 1 | 0.88 | [0.33, 2.36] | - | 0.0% | - | 0.000 |
| MZR+RASI vs MZR | 1 | 1.51 | [0.59, 3.86] | - | 0.0% | - | 0.000 |
| MMF vs STE | 2 | 2.15 | [0.69, 6.66] | 1.44 | 30.6% | 0.27 | 0.230 |
| Nefecon vs Placebo | 1 | 2.00 | [0.92, 4.33] | - | 0.0% | - | 0.000 |

Abbreviations: MMF, mycophenolate mofetil; STE, steroids; RASI, renin-angiotensin system inhibitors; LEF, leflunomide; CsA, Cyclosporin A; MZR, mizoribine; HCQ, hydroxychloroquine; AZA, azathioprine; TAC, Tacrolimus; SGLT2I, sodium glucose cotransporter 2 inhibitor.

S6-B clinical remission

| **Contrast** | **No. of study** | **RR** | **95%CI** | **Q** | ***I*^2^** | **tau^2^** | ***P*** |
| --- | --- | --- | --- | --- | --- | --- | --- |
| Placebo vs CsA | 2 | 0.67 | [0.23, 1,97] | 5.33 | 40.9% | 0.26 | 0.193 |
| MMF vs STE | 1 | 1.25 | [0.57, 2.74] | - | 0.0% | - | 0.000 |
| LEF vs RASI | 1 | 0.98 | [0.65, 1.46] | - | 0.0% | - | 0.000 |
| Placebo vs RASI | 1 | 0.17 | [0.05, 0.51] | - | 0.0% | - | 0.000 |
| RASI vs STE | 1 | 0.15 | [0.04, 0.64] | - | 0.0% | - | 0.000 |
| LEF vs MMF | 1 | 0.92 | [0.57, 1.49] | - | 0.0% | - | 0.000 |
| Placebo vs AZA | 1 | 0.81 | [0.65, 1.00] | - | 0.0% | - | 0.000 |
| Placebo vs STE | 1 | 0.19 | [0.06, 0.81] | - | 0.0% | - | 0.000 |
| Placebo vs RIT | 1 | 1.00 | [0.23, 4.27] | - | 0.0% | - | 0.000 |
| Placebo vs Sibeprenlimab | 1 | 0.10 | [0.01, 0.74] | - | 0.0% | - | 0.000 |
| Placebo vs STE+MMF | 1 | 0.15 | [0.02, 1.09] | - | 0.0% | - | 0.000 |
| RASI vs RASI+STE | 2 | 0.45 | [0.27, 0.73] | 0.76 | 0.0% | 0.00 | 0.385 |
| RASI vs Sparsentan | 1 | 0.57 | [0.50, 0.66] | - | 0.0% | - | 0.000 |
| Placebo vs HCQ | 2 | 0.50 | [0.37, 0.69] | 1.07 | 6.3% | 0.01 | 0.302 |
| Placebo vs MMF | 3 | 0.44 | [0.25, 0.79] | 0.73 | 0.0% | 0.00 | 0.694 |
| Placebo vs MZR | 3 | 0.91 | [0.70, 1.18] | 3.19 | 37.2% | 0.02 | 0.203 |
| STE vs RASI+STE | 1 | 0.93 | [0.72, 1.19] | - | 0.0% | - | 0.000 |
| STE vs TSP | 2 | 0.56 | [0.43, 0.70] | 0.21 | 0.0% | 0.00 | 0.648 |

Abbreviations: TSP, tonsillectomy with steroid pulse therapy; MMF, mycophenolate mofetil; STE, steroids; RASI, renin-angiotensin system inhibitors; LEF, leflunomide; CsA, Cyclosporin A; MZR, mizoribine; RIT, rituximab; HCQ, hydroxychloroquine; AZA, azathioprine.

S6-C ESRD or KD

| **Contrast** | **No. of study** | **RR** | **95%CI** | **Q** | ***I*^2^** | **tau^2^** | ***P*** |
| --- | --- | --- | --- | --- | --- | --- | --- |
| Placebo vs CsA | 1 | 0.31 | [0.03,2.78] | - | 0.0% | - | 0.000 |
| Placebo vs STE+AZA | 1 | 3.00 | [1.53, 5.86] | - | 0.0% | - | 0.000 |
| RASI vs STE | 1 | 5.09 | [0.61, 42.17] | - | 0.0% | - | 0.000 |
| Placebo vs RASI | 4 | 3.59 | [2.05, 6.30] | 1.14 | 0.0% | 0.00 | 0.767 |
| RASI vs STE+RASI | 1 | 7.70 | [1.01, 58.99] | - | 0.0% | - | 0.000 |
| Placebo vs MMF | 5 | 1.47 | [0.58, 3.76] | 10.23 | 60.9% | 0.66 | 0.037 |
| Placebo vs LEF | 1 | 2.00 | [0.67, 6.00] | - | 0.0% | - | 0.000 |
| Placebo vs STE | 4 | 1.98 | [1.21, 3.22] | 5.77 | 48.0% | 0.11 | 0.124 |
| Placebo vs HCQ | 1 | 0.33 | [0.04, 3.04] | - | 0.0% | - | 0.000 |
| Placebo vs MZR | 1 | 1.00 | [0.07, 14.95] | - | 0.0% | - | 0.000 |
| Placebo vs Nefecon | 1 | 1.86 | [1.14, 3.03] | - | 0.0% | - | 0.000 |
| STE vs RASI+STE | 1 | 6.29 | [0.82, 47.90] | - | 0.0% | - | 0.000 |
| RASI vs Sparsentan | 1 | 1.86 | [0.76, 4.56] | - | 0.0% | - | 0.000 |
| Placebo vs SGLT2I | 1 | 3.43 | [1.42, 8.28] | - | 0.0% | - | 0.000 |
| STE vs STE+AZA | 2 | 0.86 | [0.53, 1.39] | 0.01 | 0.0% | 0.00 | 0.937 |

Abbreviations: ESRD, end-stage renal disease; MMF, mycophenolate mofetil; STE, steroids; RASI, renin-angiotensin system inhibitors; LEF, leflunomide; CsA, Cyclosporin A; MZR, mizoribine; HCQ, hydroxychloroquine; AZA, azathioprine; SGLT2I, sodium glucose cotransporter 2 inhibitor.

S6-D 24-h UPE

| **Contrast** | **No. of study** | **RR** | **95%CI** | **Q** | ***I*^2^** | **tau^2^** | ***P*** |
| --- | --- | --- | --- | --- | --- | --- | --- |
| Placebo vs MMF | 4 | 0.82 | [-0.97, 2.61] | 56.34 | 94.7% | 3.13 | 0.000 |
| RASI vs STE | 1 | 0.39 | [0.01, 0.77] | - | 0.0% | - | 0.000 |
| LEF vs STE | 2 | -0.23 | [-0.69, 0.24] | 2.63 | 62.0% | 0.070 | 0.105 |
| Placebo vs RASI | 5 | 0.67 | [0.42, 0.91] | 1.44 | 0.0 | 0.00 | 0.837 |
| STE vs STE+AZA | 4 | 0.22 | [-0.77, 1.22] | 29.04 | 89.7% | 0.91 | 0.000 |
| LEF vs MMF | 1 | 0.07 | [-0.55, 0.69] | 0 | 0.0% | - | 0.000 |
| Placebo vs STE | 5 | 0.73 | [-0.43, 1.89] | 159.17 | 97.5% | 1.69 | 0.000 |
| Placebo vs TAC | 1 | 0.24 | [-0.38, 0.86] | - | 0.0% | - | 0.000 |
| Placebo vs Atacicept | 1 | 0.80 | [-0.50, 2.10] | - | 0.0% | - | 0.000 |
| Placebo vs Telitacicept | 1 | 5.37 | [3.73, 7.00] | - | 0.0% | - | 0.000 |
| Placebo vs CsA | 1 | 0.32 | [-0.25, 0.89] | - | 0.0% | - | 0.000 |
| Placebo vs MZR | 2 | -0.16 | [-0.83, 0.51] | 2.82 | 64.5 % | 0.15 | 0.093 |
| Placebo vs Iptacopan | 1 | 0.82 | [0.25, 1.40] | - | 0.0% | - | 0.000 |
| Placebo vs HCQ | 2 | 0.75 | [0.26, 1.25] | 2.67 | 62.6% | 0.08 | 0.102 |
| RASI vs STE+RASI | 2 | 0.94 | [0.65, 1.23] | 0.10 | 0.0% | 0.00 | 0.751 |
| Placebo vs STE+MMF | 1 | 0.98 | [0.38, 1.59] | - | 0.0% | - | 0.000 |
| STE vs STE+RASI | 1 | 2.00 | [1.21, 2.79] | - | 0.0% | - | 0.000 |
| Placebo vs RIT | 1 | -0.02 | [-0.69, 0.65] | - | 0.0% | - | 0.000 |

Abbreviations: 24 hours urinary protein excretion; MMF, mycophenolate mofetil; STE, steroids; RASI, renin-angiotensin system inhibitors; LEF, leflunomide; CsA, Cyclosporin A; MZR, mizoribine; RIT, rituximab; HCQ, hydroxychloroquine; AZA, azathioprine.

**S7: Table 7 Evaluation of meta-regression**

S7-A adverse events

| **Variables** | **Comparison** | **Regression coefficient** | **95%CI** |
| --- | --- | --- | --- |
| Publication  year | A vs B | 1.10856 | (-0.23098, 2.4035) |
|  | A vs C | -43.28218 | (**-118.90350, -0.9258**) |
|  | A vs D | -0.11265 | (-1.72388, 1.5997) |
|  | A vs E | 2.84156 | (-3.97948, 11.3521) |
|  | A vs F | -1.10343 | (-15.37239, 14.5876) |
|  | A vs H | 1.43015 | (**0.53968, 2.3868**) |
|  | A vs I | -0.06131 | (-1.60420, 1.5546) |
|  | A vs K | -6.54593 | (-44.47463, 15.0615) |
|  | A vs L | 0.17737 | (-0.74306, 1.2931) |
|  | A vs M | 0.04653 | (-12.31600, 17.1385) |
|  | A vs N | 1.06446 | (**0.31110, 1.9118**) |
|  | A vs O | 1.13306 | (-8.55303, 10.9249) |
|  | A vs P | -0.32437 | (-14.77850, 9.0862) |
|  | A vs R | 4.89829 | (**1.90992, 8.6151**) |
|  | A vs S | 0.99398 | (-10.79362, 24.0723) |
| Sample Size | A vs B | 0.68570 | (-0.26467, 1.732) |
|  | A vs C | -40.24459 | (**-88.07198, -2.184**) |
|  | A vs D | -5.33196 | (-16.06440, 2.836) |
|  | A vs E | 0.99351 | (-23.59845, 8.878) |
|  | A vs F | -1.13023 | (-10.36693, 6.740) |
|  | A vs H | 1.28327 | (**0.36125, 2.418**) |
|  | A vs I | -0.12796 | (-2.11771, 2.089) |
|  | A vs K | 2.98498 | (-13.57940, 20.522) |
|  | A vs L | -0.04605 | (-1.04831, 1.224) |
|  | A vs M | 8.48957 | (-8.12883, 62.982) |
|  | A vs N | 0.62257 | (-0.23628, 1.761) |
|  | A vs O | 1.53293 | (-5.28160, 11.080) |
|  | A vs P | 0.79831 | (-3.50618, 6.203) |
|  | A vs R | 6.62869 | (-2.11902, 30.657) |
|  | A vs S | 0.10411 | (-9.82236, 12.846) |

Abbreviations: A, placebo; B, AZA; C, Atacicept; D, CsA; E, HCQ; F, Iptacopan; G, LEF; H, MMF; I, MZR; J, MZR+RASI; K, Nefecon; L, RASI; M, SGLT2I; N, STE; O, STE+MMF; P, Sibeprenlimab; Q, Sparsentan; R, TAC; S, Telitacicept.

S7-B clinical remission

| **Variables** | **Comparison** | **Regression coefficient** | **95%CI** |
| --- | --- | --- | --- |
| Publication  year | A vs B | 0.96311 | (-7.952742, 7.038) |
|  | A vs C | 26.55975 | (**3.230180, 64.168**) |
|  | A vs D | 0.37028 | (-8.431438, 5.210) |
|  | A vs F | 1.38570 | (-0.006212, 2.828) |
|  | A vs G | 0.23577 | (-3.758937, 3.350) |
|  | A vs H | 2.06268 | (-0.171446, 4.498) |
|  | A vs I | 0.13125 | (-12.626998, 12.273) |
|  | A vs J | 2.88109 | (-0.166154, 5.965) |
|  | A vs K | 3.43449 | (-5.579117, 16.686) |
|  | A vs M | 3.22858 | (-13.149240, 15.468) |
| Sample Size | A vs B | 1.3087 | (-1.24026, 3.906) |
|  | A vs C | 21.7089 | (-2.08408, 52.485) |
|  | A vs D | 1.5128 | (-0.35575, 3.419) |
|  | A vs F | 2.2297 | (**0.01539, 3.157**) |
|  | A vs G | 0.4970 | (-1.82443, 2.651) |
|  | A vs H | 1.4524 | (-0.32148, 1.344) |
|  | A vs I | -0.0980 | (-9.31341, 9.312) |
|  | A vs J | 2.5376 | (0.63721, 4.384) |
|  | A vs K | 2.6056 | (-5.34035, 9.401) |
|  | A vs M | 3.1006 | (**0.16781, 6.818**) |

Abbreviations: A, Placebo; B, AZA; C, CsA; D, HCQ; E, LEF; F, MMF; G, MZR; H, RASI; I, RIT; J, STE; K, STE+MMF; L, STE+RASI; M, Sibeprenlimab; N, Sparsentan; O, TSP.

S7-C ESRD or KD

| **Variables** | **Comparison** | **Regression coefficient** | **95%CI** |
| --- | --- | --- | --- |
| Publication  year | A vs B | 30.6447 | (**1.4206, 81.6997**) |
|  | A vs C | 26.6211 | (-1.0777, 69.1591) |
|  | A vs D | -0.2501 | (-11.0606, 18.9266) |
|  | A vs E | -0.5216 | (-1.6105, 0.5293) |
|  | A vs F | -0.2637 | (-17.6600, 11.5125) |
|  | A vs G | -3.1338 | (-28.9290, 9.2073) |
|  | A vs H | -0.9993 | (-2.5039, 0.4317) |
|  | A vs I | -1.5186 | (-19.1285, 17.1925) |
|  | A vs J | -1.2490 | (**-2.4371, -0.3814**) |
|  | A vs K | -1.1505 | (-3.0655, 0.3466) |
| Sample Size | A vs B | 30.93144 | (**0.5650, 102.6810**) |
|  | A vs C | 26.13148 | (**0.8046, 63.0330**) |
|  | A vs D | -0.85364 | (-4.7929, 3.1474) |
|  | A vs E | -0.98980 | (-2.4524, 0.4270) |
|  | A vs F | -0.82987 | (-18.8053, 8.2107) |
|  | A vs G | -0.68619 | (-12.4799, 11.4377) |
|  | A vs H | -1.22462 | (-3.1142, 0.9742) |
|  | A vs I | -2.53884 | (-25.3050, 9.2273) |
|  | A vs J | -1.81767 | (**-3.0009, -0.7591**) |
|  | A vs K | -2.02639 | (**-3.8453, -0.4409**) |

Abbreviations: A, Placebo; B, CsA; C, HCQ; D, LEF; E, MMF; F, MZR; G, Nefecon; H, RASI; I, SGLT2I; J, STE; K, STE+AZA; L, STE+RASI; M, Sparsentan.

S7-D 24-h UPE

| **Variables** | **Comparison** | **Regression coefficient** | **95%CI** |
| --- | --- | --- | --- |
| Publication  year | A vs B | 0.341228 | (-18.7452, 14.7351) |
|  | A vs C | 0.212449 | (-3.6871, 5.0319) |
|  | A vs D | 0.427840 | (-4.8147, 7.3358) |
|  | A vs E | 6.405695 | (-7.0258, 44.7947) |
|  | A vs G | 0.270645 | (-15.37239, 1.1591) |
|  | A vs H | 0.091646 | (-2.8464, 3.3352) |
|  | A vs I | 0.698372 | (-0.0984, 1.5424) |
|  | A vs J | -0.497385 | (-13.9374, 9.9348) |
|  | A vs K | 0.587820 | (-0.0236, 1.2239) |
|  | A vs L | -0.077000 | (-1.2666, 1.1732) |
|  | A vs M | 3.049105 | (-1.2666, 1.1732) |
|  | A vs P | -1.291043 | (-27.9982, 45.4127) |
| Sample Size | A vs B | 0.97679 | (-10.6270, 13.356) |
|  | A vs C | 0.28737 | (-4.2583, 5.707) |
|  | A vs D | 0.90908 | (-1.1810, 2.863) |
|  | A vs E | 0.45707 | (-3.7685, 4.634) |
|  | A vs G | 0.59948 | (-1.7220, 3.445) |
|  | A vs H | -0.05493 | (-2.2735, 1.929) |
|  | A vs I | 0.65402 | (-0.5430, 1.559) |
|  | A vs J | 4.04829 | (-9.1861, 66.150) |
|  | A vs K | 0.46734 | (-2.0606, 1.366) |
|  | A vs L | 0.54665 | (-2.2719, 2.026) |
|  | A vs M | 2.00772 | (-3.1765, 20.204) |
|  | A vs P | 0.54269 | (-13.6142, 7.547) |

Abbreviations: A, Placebo; B, Atacicept; C, CsA; D, HCQ; E, Iptacopan; F, LEF; G, MMF; H, MZR; I, RASI; J, RIT; K, STE; L, STE+AZA; M, STE+MMF; N, STE+RASI; O, TAC; P, Telitacicept.

**Supplementary Figure 8-a: Plot of SUCRA values coordinates combining CR and 24-hour UPE**


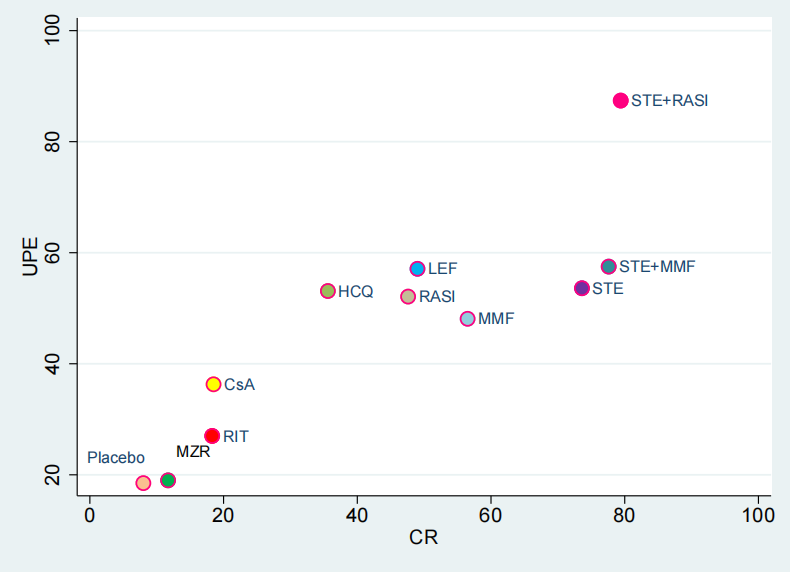


**Supplementary Figure 8-b: Plot of SUCRA values coordinates combining ESRD or KD and AEs.**


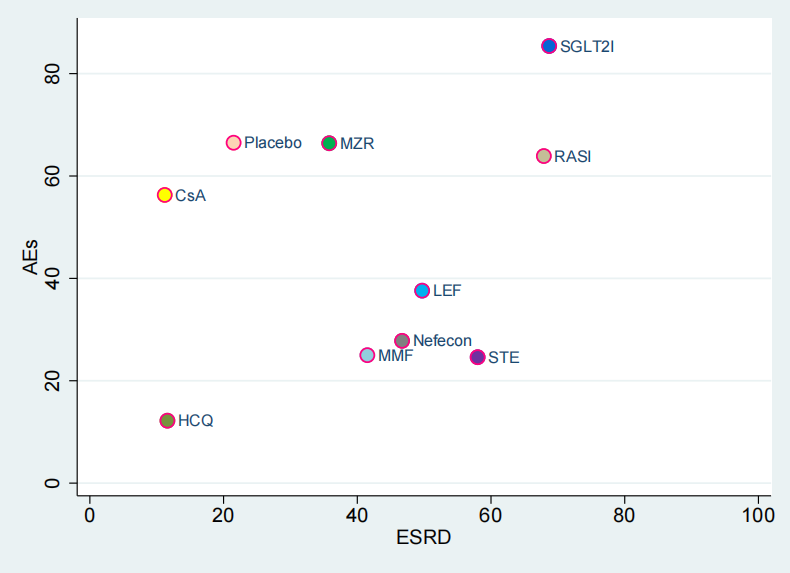


S8: Table 1 SUCRA value of adverse events, clinical remission, ESRD or KD and 24-h UPE

| **Treatment** | **Adverse events** | | | **Clinical remission** | | | **ESRD or KD** | | | **24-h UPE** | | |
| --- | --- | --- | --- | --- | --- | --- | --- | --- | --- | --- | --- | --- |
|  | **SUCRA** | **PrBest** | **MeanRank** | **SUCRA** | **PrBest** | **MeanRank** | **SUCRA** | **PrBest** | **MeanRank** | **SUCRA** | **PrBest** | **MeanRank** |
| **Iptacopan** | 88.4 | 56.1 | 3.1 | NR | NR | NR | NR | NR | NR | 53.1 | 0.1 | 8.0 |
| **SGLT2i** | 85.4 | 13.2 | 3.6 | NR | NR | NR | 68.7 | 2.1 | 4.8 | NR | NR | NR |
| **Atacicept** | 83.2 | 15.5 | 4 | NR | NR | NR | NR | NR | NR | 49.4 | 0.2 | 8.6 |
| **Telitacicept** | 70.1 | 4.8 | 6.4 | NR | NR | NR | NR | NR | NR | 99.9 | 98.7 | 1 |
| **Placebo** | 66.5 | 0.0 | 7 | 8.0 | 0.0 | 13.9 | 21.5 | 0.0 | 10.4 | 18.5 | 0.0 | 13.2 |
| **MZR** | 66.4 | 5.6 | 7 | 11.7 | 0.0 | 13.4 | 35.8 | 2,6 | 8.7 | 19.0 | 0.0 | 13.2 |
| **RASI** | 63.9 | 0.2 | 7.5 | 47.6 | 0.0 | 8.3 | 67.9 | 0 | 4.8 | 52.1 | 0.0 | 8.2 |
| **Sparsentan** | 59.9 | 1.6 | 8.2 | 72.2 | 0.1 | 4.9 | 82.6 | 7.8 | 3.1 | NR | NR | NR |
| **CsA** | 56.3 | 0.7 | 8.9 | 18.5 | 0.0 | 12.4 | 11.2 | 0.2 | 11.7 | 36.3 | 0.0 | 10.6 |
| **Sibeprenlimab** | 55.9 | 0.2 | 8.9 | 85.6 | 45.9 | 3 | NR | NR | NR | NR | NR | NR |
| **MZR+RASI** | 45.2 | 1.1 | 10.9 | NR | NR | NR | NR | NR | NR | NR | NR | NR |
| **AZA** | 40.4 | 0.0 | 11.7 | 23.6 | 0.0 | 11.7 | NR | NR | NR | NR | NR | NR |
| **STE+MMF** | 38.6 | 0.2 | 12.0 | 77.6 | 27.5 | 4.1 | NR | NR | NR | 57.5 | 0.2 | 7.4 |
| **LEF** | 37.6 | 0.1 | 12.2 | 49.0 | 0.0 | 8.1 | 49.7 | 0.8 | 7.0 | 57.1 | 0 | 7.4 |
| **Nefecon** | 27.8 | 0.1 | 14.0 | NR | NR | NR | 46.7 | 0.1 | 7.4 | NR | NR | NR |
| **MMF** | 25.0 | 0.0 | 14.5 | 56.5 | 0.0 | 7.1 | 41.5 | 0.0 | 8.0 | 48.1 | 0.0 | 8.8 |
| **STE** | 24.6 | 0.0 | 14.6 | 73.6 | 0.0 | 4.7 | 58.0 | 0.0 | 6.0 | 53.6 | 0.0 | 8.6 |
| **HCQ** | 12.2 | 0.6 | 16.8 | 35.6 | 0.0 | 10.0 | 11.6 | 0.3 | 11.6 | 53.1 | 0.0 | 8.0 |
| **TAC** | 2.5 | 0.0 | 18.6 | NR | NR | NR | NR | NR | NR | 34.2 | 0.0 | 10.9 |
| **TSP** | NR | NR | NR | 92.8 | 26.3 | 2 | NR | NR | NR | NR | NR | NR |
| **STE+RASI** | NR | NR | NR | 79.4 | 0.1 | 3.9 | 98.1 | 86 | 1.2 | 87.4 | 0.7 | 2.9 |
| **RIT** | NR | NR | NR | 18.3 | 0.0 | 12.4 | NR | NR | NR | 27.0 | 0.0 | 11.9 |
| **STE+AZA** | NR | NR | NR | NR | NR | NR | 56.5 | 0.1 | 6.2 | 53.7 | 0.0 | 7.9 |

Abbreviations: ESRD, end-stage renal disease; SUCRA, surface under the cumulative ranking curve; 24-h UPE, 24-hour urinary protein excretion; TSP, tonsillectomy with steroid pulse therapy; MMF, mycophenolate mofetil; STE, steroids; RASI, renin-angiotensin system inhibitors; LEF, leflunomide; CsA, Cyclosporin A; MZR, mizoribine; RIT, rituximab; HCQ, hydroxychloroquine; AZA, azathioprine; TAC, Tacrolimus; SGLT2i, sodium glucose cotransporter 2 inhibitor.
